# A neurobehavioral study on the efficacy of price interventions in promoting healthy food choices among low socioeconomic families

**DOI:** 10.1038/s41598-020-71082-y

**Published:** 2020-09-22

**Authors:** Tannista Banerjee, Veena Chattaraman, Hao Zou, Gopikrishna Deshpande

**Affiliations:** 1 Department of Economics, Auburn University, 140 Miller Hall, Auburn, AL 36849 USA; 2 Department of Consumer and Design Sciences, Auburn University, Auburn, AL USA; 3 Department of Electrical and Computer Engineering, AU MRI Research Center, Auburn University, 560 Devall Dr, Suite 266D, Auburn, AL 36849 USA; 4 Department of Psychological Sciences, Auburn University, Auburn, AL USA; 5 Alabama Advanced Imaging Consortium, University of Alabama, Birmingham, AL USA; 6 Center for Health Ecology and Equity Research, Auburn University, Auburn, AL USA; 7 Center for Neuroscience, Auburn University, Auburn, AL USA; 8 School of Psychology, Capital Normal University, Beijing, China; 9 Key Laboratory for Learning and Cognition, Capital Normal University, Beijing, China; 10 Department of Psychiatry, National Institute of Mental Health and Neurosciences, Bangalore, India

**Keywords:** Neuroscience, Health policy, Nutrition, Risk factors

## Abstract

Given the healthcare costs associated with obesity (especially in childhood), governments have tried several fiscal and policy interventions such as lowering tax and giving rebates to encourage parents to choose healthier food for their family. The efficacy of such fiscal policies is currently being debated. Here we address this issue by investigating how behavioral and brain-based responses in parents with low socioeconomic status change when rebates and lower taxes are offered on healthy food items. We performed behavioral and brain-based experiments, with the latter employing electroencephalography (EEG) acquired from parents while they shop in a simulated shopping market as well as follow up functional magnetic resonance imaging (fMRI) in the more restricted scanner environment. Behavioral data show that lower tax and rebate on healthy foods increase their purchase significantly compared to baseline. Rebate has a higher effect than lower tax treatment. From the EEG and fMRI experiments, we first show that healthy/unhealthy foods elicit least/maximal reward response in the brain, respectively. Further, by offering lower tax or rebate on healthy food items, the reward signal for such items in the brain is significantly enhanced. Second, we demonstrate that rebate is more effective than lower tax in encouraging consumers to purchase healthy food items, driven in part, by higher reward-related response in the brain for rebate. Third, fiscal interventions decreased the amount of frontal cognitive control required to buy healthy foods despite their lower calorific value as compared to unhealthy foods. Finally, we propose that it is possible to titrate the amount of tax reductions and rebates on healthy food items so that they consistently become more preferable than unhealthy foods.

## Introduction

Obesity is a growing problem in both developing and developed nations all over the world. According to the data provided by U.S. Department of Health and Human Services, approximately 300,0000 deaths are caused by obesity in United States alone^1^. Obesity increases the risk of heart disease, high blood pressure, diabetes, cancer, sleep apnea, arthritis, pregnancy complications, and many other disorders^2^. Also, the fact that a higher body-mass index (BMI) in developmental years increases the risk of adult obesity, makes the disease worse for children^3,4^. Childhood obesity is of particular concern, and especially a rising one among rural and low-income populations. Individuals from lower income and socioeconomic status, as well as individuals residing in rural areas are likely to have lower health literacy than their counterparts, and thus are less aware of the potential harmful effects of unhealthy foods on their health status^5^. In addition, since children rely on their parents for food, parents’ choices of the food supply for the whole family directly affect childhood obesity levels. In 2008, the aggregate national medical and insurance cost associated with overweight and obesity conditions was $113.9 billion. This is approximately 5.0% to 10% of U.S. health care spending^6–8^.

Several fiscal policies have been employed by federal and state governments to encourage individuals to purchase more healthy food^9–13^. Among these, increasing the tax on unhealthy foods and providing rebates on healthy foods are the most frequently employed strategies^14–17^. Researchers have studied the influence of higher taxes on unhealthy foods in combination with increased subsidy on healthy foods on consumers’ food choices; however, the results are not straight-forward due to the complexity of consumer behavior and underlying substitution effects^18^. For example, when higher taxes were applied to unhealthy foods such as candy and soda, consumers substituted their purchase with other higher calorie foods^19,20^. This resulted in the same or greater amounts of calories purchased when the intervention was present vs. absent. Also, several previous behavioral studies showed that unless the differential tax on unhealthy foods is abnormally and unrealistically high, their effect on reducing unhealthy food choices was small^17,21–25^. Higher taxes on unhealthy food items has also attracted some moral debate because it results in low-income and vulnerable populations spending more money on necessities, such as food. Due to the above-mentioned reasons, lowered tax on healthy foods has been proposed as an alternative fiscal policy and represents how the current study operationalizes differential tax.

A pilot study by Epstein et al.^26^ found that when tax was lowered on healthy food items, parents were more likely to purchase more healthy foods for their family; but if they were required to spend the money saved in the store immediately, then they used this money to buy more unhealthy foods. As a result, if consumers do not have full control of the saved money, the desired effect of promoting healthy food purchase may not be obtained. An alternative fiscal tool to lowering taxes on healthy foods, is offering a rebate on the same. A rebate is a certain amount of money refunded to consumers when they purchase certain goods and services. Studies have shown that rebates are not as helpful as expected in encouraging the purchase of healthy foods^9,10^. Specifically, although individuals purchase more healthy food when it is subsidized, they simultaneously spend the saved money on buying additional unhealthy alternatives^9^. These results suggest that lowered taxes and rebates on healthy foods have a similar effect of promoting greater purchase of healthy foods, but the amount saved is often spent on unhealthy items. These findings may hold true if the consumers have a fixed amount or budget to spend. However, no previous studies have compared the differential effectiveness of these two fiscal measures in a single study, while not stipulating a budgeted amount to spend.

In this paper, we combined the two fiscal interventions and investigated how parents’ behavior and brain-based responses change when rebates and lower differential taxes (henceforth referred to as low tax) are offered on healthy food items. Consumers will be more price-sensitive as their income decreases^27–29^. Therefore, we can expect that individuals with lower socioeconomic status will be more receptive to fiscal incentives as compared to individuals with higher socioeconomic status since median incomes in the former case are generally lower than in the latter case. The two price interventions of rebates and lower taxes were framed differently. While lower taxes were framed as a discount on healthy food purchases, rebate was framed as “cash back”. Consequently, even though the net amount of money saved by choosing healthy foods may be the same, we argue that the framing of the intervention as a rebate or cash back, can affect the benefit perceived from the intervention and thus affect choice behavior. Specifically, we hypothesize that framing the fiscal intervention, as a rebate/cash back rather than a discount, will be more effective in promoting healthy food choice among low socioeconomic status consumers.

The propositions of prospect theory^30^ and theory of mental accounting^31^ provide the rationale for the above hypothesis. Based on the seminal work of prospect theory, the theory of mental accounting posits that the framing of outcomes as integrated wholes (i.e., discounted price) versus segregated components (i.e., base price plus rebate or ‘cash back’) affects how decision-makers valuate the gain; such that, for small price reductions, a rebate is valued higher than a discount^32^. Thaler explains this effect based on the ‘silver lining principle’: when faced with *mixed losses* (loss with a smaller gain) decision makers prefer to separate the loss and the small gain in their mental accounting so that the small gain (i.e., rebate) can serve as a *silver lining* for the larger loss (i.e., the cost of the product). On the contrary, lower tax on an item is just perceived as a smaller loss, but a loss nevertheless. Therefore, it does not gain any perceptual advantage from the silver lining effect. Consequently, based on the ‘silver lining principle’, we expect that framing the healthy food purchase incentive as a cashback or rebate rather than a discount or lowered tax will have greater effect in promoting healthy food choice.

In order to provide mechanistic insight on this anticipated effect, we investigated the underlying neurobiology of this choice under the two fiscal interventions in addition to a control, where no fiscal intervention was employed. Two non-invasive neuroimaging methods with complementary strengths, fMRI (Functional Magnetic Resonance Imaging) and electroencephalography (EEG), were employed to address this purpose.

Functional MRI or fMRI is a non-invasive way of inferring brain activity in awake humans. It provides vastly superior spatial resolution as compared to modalities such as EEG, which coupled with its high sensitivity, has enabled researchers to probe human brain function and develop mechanistic models of behavior^33^. Previous studies have employed fMRI to identify brain regions that activate in response to food-related stimuli^34–36^. These studies are useful as they have shown how obese individuals might process food-related information differently^37–40^. Specifically, these studies have noted the hypersensitivity of the brain’s reward circuit to food stimuli in obese individuals. However, no fMRI studies have investigated the effects of rebates and lower taxes (or other differential taxes) on parents’ brain-based response and food choice behavior. Hence, brain mechanisms underlying the efficacy of public policy interventions using fiscal tools (lowered taxes and rebates) remains completely unexplored. Therefore, the aim of this work is to compare the effectiveness of lowered taxes and rebates on healthy foods in promoting consumption of healthy food among lower income populations.

Specifically, we test the following hypotheses in the neuroimaging experiment. First, we hypothesize that healthy food items will elicit least reward response and unhealthy food items will lead to highest reward response. This is because unhealthy foods have higher calorific value, which is encoded by the brain’s reward system^37,41–48^. Further, by offering lower tax or rebate on healthy food items, the reward response in the brain for such items will be significantly enhanced. This is based on the assumption that the role of fiscal interventions (rebate or lower tax) is to offset the low reward signal for healthy foods due to their lower calorific value, by enhancing perceived reward value for healthy products by associating it with a monetary reward. Many previous studies have shown that the brain’s reward network also encodes monetary reward value. For example, ventral striatum was shown to be modulated by the magnitude of monetary reward^49^ as well as food reward^35^. Another study showed that both substantial nigra and orbitofrontal cortex had a stronger activation when provided with monetary reward than verbal reward^50^. Second, we hypothesize that rebate will be more effective than lower tax in encouraging consumers to purchase healthy food items, driven in part, by higher reward-related response in the brain for rebate in comparison to lower tax. Our hypothesis aims to uncover the mechanistic basis for this observation, which is expected from prospect theory and the *silver lining effect* as discussed before. Third, we surmise that executive control processes will follow the opposite pattern, i.e. highest executive control response will be needed to purchase healthy items as opposed to unhealthy items. This follows from the fact that since healthy items have low reward (or calorific value), top-down processes must exert their influence for such items to be purchased. This is based on previous data indicating how lateral frontal cortical regions need to exert top-down executive control to override automatic responses chosen by bottom-up processes driven by sub-cortical structures^51–54^. These regions are also thought to underlie self-control involving modulation of value signals^34^. Consequently, by providing lower tax or rebate, the amount of executive control needed to purchase healthy food items with low reward value will be reduced. Finally, while both reward and executive control related responses can be measured using fMRI, we believe that EEG will be appropriate to measure only responses underlying executive control since they rely on cortical sources while reward-related responses are more likely to be involving deep brain structures such as the striatum. Consequently, we hypothesize that frontal alpha power obtained from EEG will capture the executive control responses for each condition as mentioned above. We decided to concentrate on the frontal alpha band given previous literature implicating its role in top-down inhibition and executive control^55–57^.

We concentrate on the state Alabama because Alabama is currently facing an obesity epidemic. The rural population within this state is particularly vulnerable because of poverty and lack of access to healthy food. According to the Centers for Disease Control and Prevention’s survey “Alabama Behavior Risk Factor Surveillance System (BRFSS)”^58^, 14 of Alabama’s counties have over 40% adult obesity rates. In the year of 2013, Alabama Youth Risk Behavior Survey depicted that children in Alabama are at particular risk^59^. The report showed that 17% of children in Alabama are obese whereas the national average is only 13.1%.

## Materials and methods

### Participants

This study was primarily interested in the effect of price interventions on purchase choices made by parents with low socioeconomic status. Therefore, we recruited participants from low-income families in Talladega County, Macon County, Chambers County and Lee County of Alabama.

Participants were selected in a randomized order representing each county equally. Family resource centers inside the target counties helped with participant recruitment through their organized family oriented programs for those counties. These counties were selected because their obesity prevalence rate is higher than the national average, but differ from one another. For example, Lee County had male and female obesity prevalence rate at 38 and 39 percent, respectively, whereas Talladega County had male and female obesity prevalence rate of 40 and 48 percent, respectively, in 2011^60^. Participants were between the ages of 18 and 60 and a parent of a child or children between the ages of 2 and 18 years. Participants had a household income lower than the poverty threshold as described by the U.S. Department of Health and Human Services (as shown in Table 1).

**Table 1:** Family/household income criteria used for recruiting participants.

Persons in family/household	Income limit for recruitment qualification
1	$11,770
2	$15,930
3	$20,090
4	$24,250
5	$28,410
6	$32,570
7	$36,730
8	$40,890

For families/households with more than 8 persons, $4,160 was added for each additional person.

### Recruitment procedure

Participants were first pre-screened for behavioral and MRI compatibility. Pre-screening included an exhaustive questionnaire documenting whether the participant (i) had any medical condition that prevented him/her from finishing an MRI scan before, (ii) had been injured by a metallic object or a foreign body before, (iii) had been implanted by a medical device within their body before, (iv) had any tattoo /permanent makeup that contains metal or body-piercing jewelry that cannot be removed. Participants who self-reported to be claustrophobic were also excluded from the study. In total 52 participants were selected for the behavioral-cum-EEG experiment (referred to as Experiment-1). From these 52 participants, 19 participants (13 females and 6 males, ages 37.7 ± 10.5) participated in the MRI experiment (referred to as Experiment-2).

All experimental methods and procedures were approved by the Auburn University Institutional Review Board (IRB). The experiments were performed in conformance with expected international ethical standards. All participants provided informed consent for participating in the study. After both experiments, the participants were compensated financially for their participation. Further, the individuals in any of the figures have provided specific written informed consent for their pictures to be published in an online open access publication.

### Motivation for multimodal imaging

Notwithstanding the advantages that fMRI provides for investigating brain function, it is limited by the restrictive environment and artificial constraints that it imposes on experimental design. Specifically, during an fMRI experiment, individuals will be required to lie down on the scanner in a supine position with their head encapsulated in a radiofrequency coil such that they are unable to move. Products available for purchase and their associated price promotions are displayed to the subjects using an MR-compatible projection system and their responses are collected using a button press. This is not a typical environment or setting in which an individual is likely to shop for groceries. Further, limits on the time in which an individual can be scanned as well as mathematical constraints arising from the way the fMRI signal is decoded, does not allow us to show as many product stimuli to the participants as in an unconstrained shopping environment. In order to address these concerns regarding the ecological validity of an fMRI experiment, we performed an additional experiment in a simulated shopping market wherein the subjects were allowed to freely move and shop for product offerings. In this experiment, the subjects wore an 8-channel wireless dry EEG cap with video-enabled goggles so that brain responses to fiscal interventions can be captured, via EEG, in a less restrictive and more naturalistic environment. Both experiments were designed to test the same hypotheses, and we hoped to unearth converging evidence from these different neuroimaging modalities, that offer differing methodological strengths.

#### Experiment-1 (behavioral-cum-EEG experiment)

##### Experimental design and stimuli

This experiment was setup at the store area of the University's Radio Frequency Identification (RFID) lab. The RFID lab has 13,000 square foot area, a section of which was converted into a simulated grocery store for this study (see Fig. 2A). Experiment-1 employed a 2 (Food type: healthy, unhealthy) × 3 (Fiscal intervention: lower tax, rebate, control) within-subjects experimental design. The food type was manipulated using replicas of 30 healthy and 30 unhealthy food items. Healthy and unhealthy foods were chosen based on the United States Department of Agriculture (USDA) guidelines for healthy food^61^ and the healthy snack calculator^61^. Food items were selected to represent typically available healthy and unhealthy food items in a grocery store. List of food items are provided in Table A1 of the Appendix.

**Figure 1:**
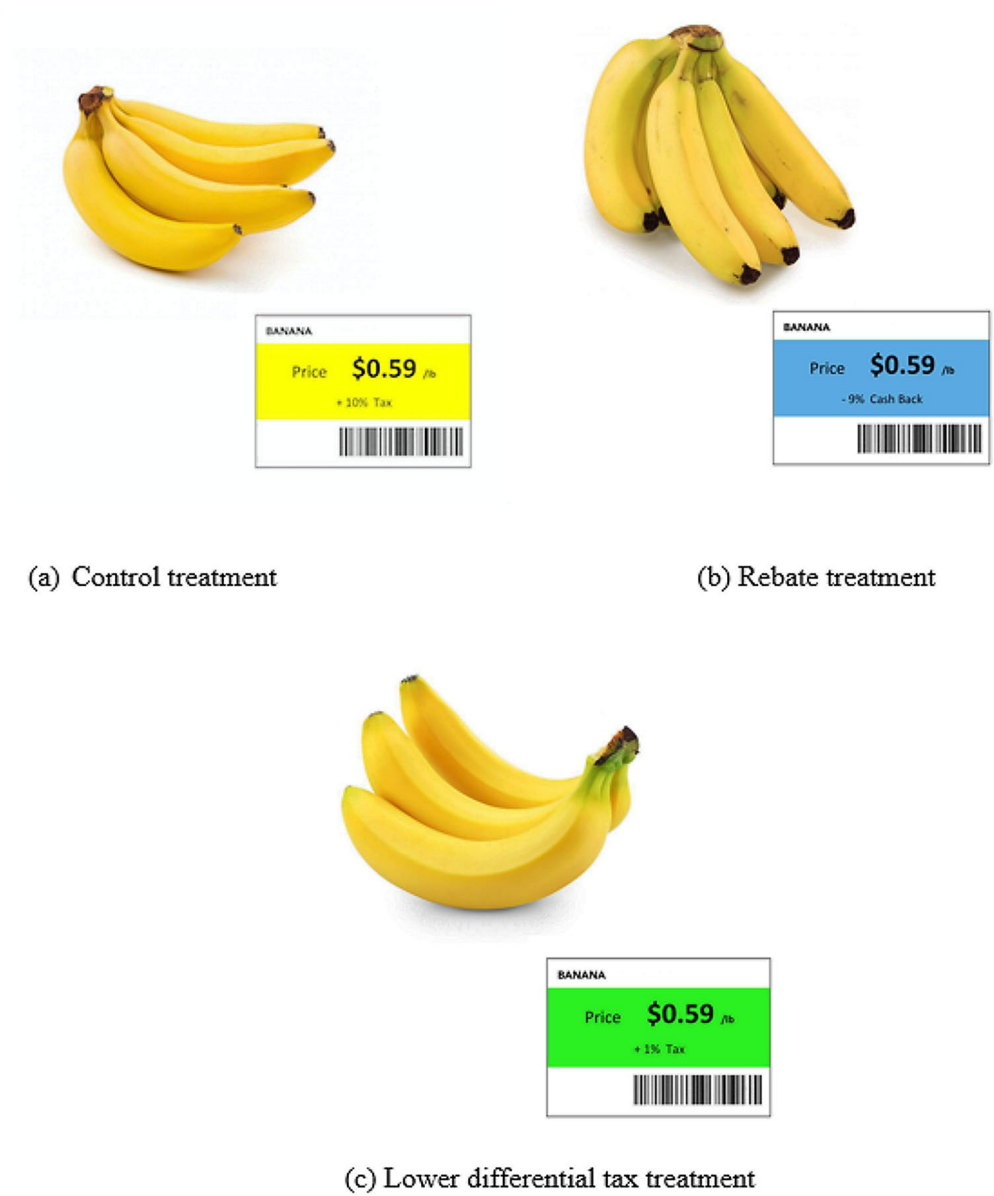
Examples of three fMRI stimulus images—control condition (**a**), rebate condition (**b**) and low differential tax condition (**c**)—corresponding to the healthy food banana. It is noteworthy that the price tags, including the color code, are the same as those used in the behavioral-EEG experiment.

Participants were assigned to the control, lower tax and rebate interventions in a randomized order to control for order effects in the within-subjects design. The fiscal interventions were created using item price labels such that: (1) for the control condition, participants were required to pay regular tax (10% in Alabama^62^) on purchased food items; (2) for the rebate condition, participants had to pay regular tax (10%) on purchased healthy and unhealthy food items, but received 9% cashback on healthy foods; and (3) for the lower tax condition, participants paid reduced tax (1%) on healthy foods and regular tax (10%) on the unhealthy foods purchased. Hence, the lower tax and rebate conditions resulted in monetarily equivalent savings. As a result, if buying a same item under both low tax and rebate condition, the net money needed is the same, which is 101% of its price. To create a realistic setting for these interventions, participants received an in-store promotional flyer (similar to a grocery store flyer, provided in Appendix) for the lower tax and rebate conditions containing images and prices of the promoted healthy food items. Along with the flyer, the details of the two in-store promotions (lower tax and rebate) were explained to create an understanding of the monetary equivalence of these two fiscal interventions before each of the three shopping sessions (control, lower tax and rebate in randomized order) began. To further differentiate the fiscal interventions, different color codes were employed for each in the flyers and price tags with blue representing a rebate promotion, green representing a lower tax promotion and yellow representing the control condition (see Fig. 1 for an example).

##### Procedure

**Figure 2:**
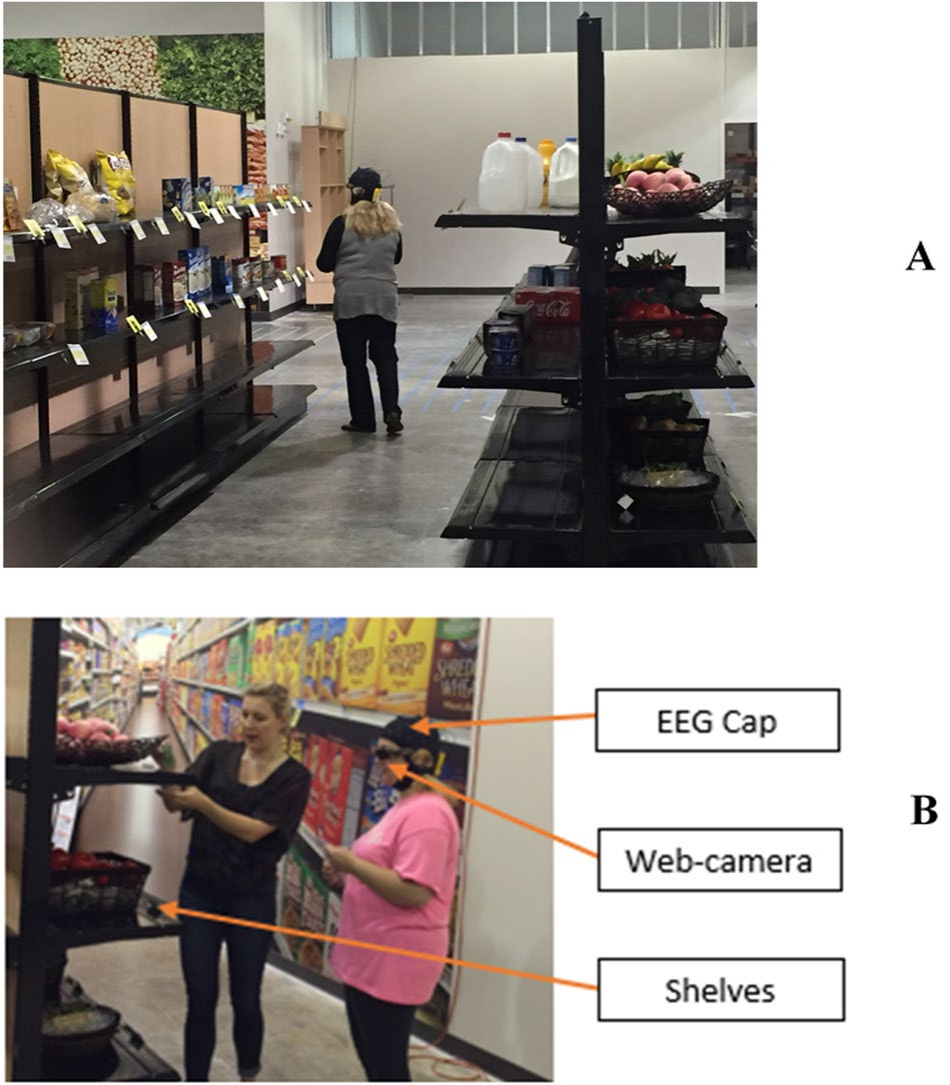
(**A**) The simulated shopping environment setup at the RFID lab in Auburn University. The shelves, products and associated price tags can be seen. (**B**) A participant wearing the EEG cap and web-camera (as noted in the figure) while the research assistant explains the experimental design to the participant. The individuals in the figure have provided written consent for their pictures to be published.

After receiving the participant’s informed consent, complete experimental instructions were provided to the participant by an investigator. Participants were requested to imagine that they have $100 and they would shop for a week’s grocery for their family. Participants were also informed that there would be three rounds of shopping for each participant, with differing in-store promotions (corresponding to each fiscal intervention). All shopping rounds had same products. But the price labels were changed in each round to reflect the color coding and the price promotion. While shopping, if the participant wished to buy a given product, he/she could pick up the label corresponding to the purchased product and place it in the shopping bag. This bag was collected from the participant after each round of shopping in order to determine their choice behavior under each fiscal intervention. Participants were not restricted to buy any specific number of items. Prior to the shopping rounds, participants were helped to don an 8-channel wireless EEG cap with dry electrodes (Enobio cap from Neuroelectrics^63^) on their head while making their shopping decision (see Fig. 2B). The impedance of the EEG electrode contacts with the scalp was less than 20 Ω. EEG data was sampled at 250 Hz. The wireless data transfer feature of the EEG cap helped participants move around freely in the store while shopping. Participants also wore a small wireless webcam while they shopped (see Fig. 2B). This was essential in order to determine the temporal segments of EEG data corresponding to the decision of whether to buy a given product or not. This determination was performed post-hoc while analyzing the data. One could argue that the webcam only tracks the position of the head and not that of the eye. In order to address this issue, products were placed only in the top shelves that can be seen at the eye level. Participants were also explicitly instructed to avoid eye saccades and make decisions regarding products only when they are directly in front of their eyes. Every participant followed the same path in the store.

After each round, the participant filled out one section of a 3-sectioned survey questionnaire (please see Appendix for the sample questionnaires; data from the questionnaires will be reported elsewhere and will not be elaborated in this report). By the end of the shopping rounds, the participants filled out all three sections of the survey. Demographic data was acquired using first section of the survey questionnaire at the beginning of the three shopping tasks. Finally, the participants were escorted to a private room of the RFID lab and their weight and height data was recorded.

##### Behavioral data analysis

The shopping data acquired from the experiment-1 was used to estimate the statistical significance of the effects of interest, i.e. rebate and lower tax (referred to as “treatments” while describing the model below) on food product purchase. Healthy and unhealthy food items purchased were obtained by summing the total number of products purchased under different food categories (denoted as “*f*”). In the behavioral data analysis, food products were categorized as follows: healthy (unhealthy) vegetables, healthy (unhealthy) fruits, healthy (unhealthy) grains, healthy (unhealthy) diary and healthy (unhealthy) proteins. The total number of food products selected under each category was taken to be the dependent variable ($\Delta {y}_{if}$ in Eq. 1 below) for data analysis. The data provides control and post-treatment observations of choice behavior on every participant for both the treatments. We estimated the following model.

1 $$\Delta y_{if} = \beta_{0} + \mathop \sum \limits_{j = 1}^{2} \beta_{1} T_{j} + \beta_{3} X_{i} + \varepsilon_{i}$$

The dependent variable $\Delta y_{if}$ presents the difference in total number of food items purchased under category *f* between control and post-treatment purchases for participant *i. Tij* represents two dummy variables (i.e. the design matrix containing binary variables). For example, the rebate dummy is 1 for rebate treatment and 0 for all other conditions. Therefore, $\beta_{1}$ captures the estimated effect of the treatment rebate holding everything else constant for lower tax and rebate treatments for participant *i* in treatment *j*. *Xi* presents demographic variables used as individual controls, described in Tables 2 and 3. A “Seemingly Unrelated Regression” (SUR^64^) approach was applied for all the dependent variables.

**Table 2:** Descriptive statistics for demographic data.

Variable	Mean	SD	Min	Max
Age	38.843	11.353	23	67
Number of people in household	3.58	1.403	1	9
Number of children in the house	2.216	1.390	1	7
Age of the oldest child	9.196	5.659	1	22
Employed	1.712	0.457	0	1
Observations	52

**Table 3:** Description of demographic variables.

Variable	Percentage
**Gender**	
Female	82.69
**Ethnicity**	
African American	80.77
Hispanic or Asian	3.85
Caucasian	15.38
**Annual Income**	
Less than $14,999	37.25
$15,000–$24,999	41.17
$25,000–$39,999	13.72
$40,000–$49,999	1.96
$49,000–$80,000	5.90
**Marital status**	
Single and never married	62
Married	22
Separated	10
Widowed	6
**BMI**	
Underweight and Normal Weight	5.60
Normal or Healthy Weight	11.10
Overweight and Obese	20.30
Obese	63.00
**Education**	
Some High School	13.0
High School Degree	46.3
Some College or Technical School	33.3
College Degree	3.7
Some Graduate School	1.9
Graduate Degree	1.9

##### EEG Data Analysis

The raw EEG data was pre-processed using standard techniques by employing “NIC software” from Neuroelectrics^65^ as well as “Brain Vision Analyzer” software^66^ from Brain Products GmbH, Germany. This involved manual inspection of data to ensure that data from all channels are usable, denoising using IIR (infinite impulse response) filters, ocular correction using independent component analysis (ICA) to remove eye blink artifacts, and temporal segmentation of the data in order to identify data corresponding to 1 s before and 2 s after the decision-making point. We were primarily interested in frontal EEG power in the alpha band (in accordance with our hypotheses described in the introduction) during decision making across different trial types. Therefore, we used the Welch method^67^ to estimate the power spectral density (PSD) from pre-processed and segmented EEG data and obtained average power across subjects in the alpha band (8–12 Hz) for each trial type, specifically in lateral frontal electrodes AF7 and AF8 (according to the 10–20 EEG electrode placement system).

#### Experiment-2 (functional MRI experiment)

##### Procedure

Upon their arrival at the MRI center and before the scan, the participants were again screened for MR-compatibility in-person and were requested to provide informed consent if they agreed to participate in the study. Subjects were informed that participation in the study was voluntary and they were free to quit the experiment at any point during the experiment without giving a reason. Additionally, they were notified that their personal information would be kept confidential in accordance with HIPPA (Health Insurance Portability and Accountability Act of 1996) regulations. A research assistant gave participants a more detailed introduction about the experiment, the task and the fiscal interventions (food item promotions), identical to the information provided for Experiment-1. To ensure comprehension of the task requirements, all participants completed a practice run prior to scanning using a laptop outside the scan room. The practice run was identical to a real run implemented during scanning, but had only five stimuli which were not used during data acquisition. Following the practice run, the participants were asked to place their head inside a 32-channel head coil (from Nova Medical, Inc.) and feel comfortable. A mirror was placed atop the coil so that the participants could view visual stimuli projected from the screen at the other end of the bore using an MR-compatible projection system (from Avotec, Inc.). Soft sponge was placed inside the coil in order to secure the head to minimize head motion. Participants were also given a squeeze ball that could be squeezed to stop the scan anytime if they wanted to exit. Participants were asked to test the MR-compatible button box (employed for indicating their purchase decision, discussed in a subsequent section) to make sure it functioned well. Participants also had some time to adjust themselves to the projector screen, as well as the scanning environment before the actual scan. The stimulus display via the projector was controlled using E-prime software (Psychology Software Tools, Inc.) on a PC connected to the scanner console, so that both stimulus presentation and data acquisition could be simultaneously triggered and temporally synchronized.

##### Functional MRI stimuli

Similar to the behavioral study, three types of fMRI stimuli were designed (rebate, lower tax, control). Each fMRI stimulus image was made up of the picture of a food item along with its price tag. The brand of the food was covered to avoid possible influence of brand preference on participants’ shopping decisions. Three different colors representing two different price interventions (rebate and lower tax) as well as the control condition were assigned to the price tags. The fMRI stimuli involved images of 36 different food items (18 healthy and 18 unhealthy foods). The number of items is lower than that in the behavioral study due to the following reasons: (a) the amount of time a participant may stay inside the scanner is limited; (b) fMRI experimental designs must include inter-trial resting periods so that the response relative to baseline can be decoded. Due to these time-related reasons, even though we employed 30 stimuli per condition in Experiment-1, we could only include 18 stimuli in the fMRI experiment. In order to prevent fatigue from seeing the same healthy food item under different fiscal interventions, three different images of the same food item were used to create stimuli corresponding to rebate, lower tax and control conditions (see Fig. 1). Since unhealthy food items were not associated with rebate or lower tax, three different images were used separately to produce three fMRI stimulus images whose price tags remained the same under the control condition. In total, there were 108 fMRI stimuli (54 belonged to unhealthy category and 54 belonged to healthy category).

##### Functional MRI task design

**Figure 3:**
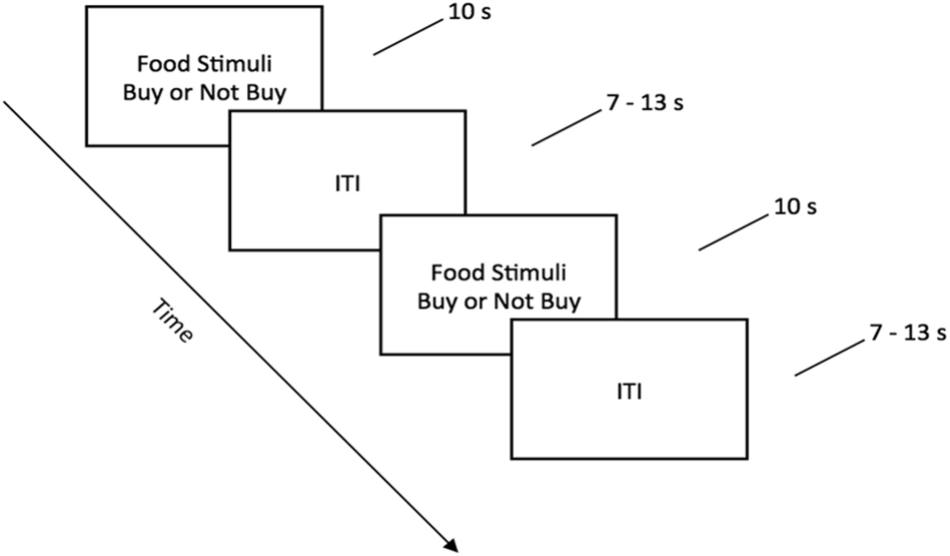
A schematic representation of the task design. fMRI stimuli are shown during the 10 s task duration and a black image with a small white cross in the middle is shown during the variable inter-trial interval.

The fMRI experimental session consisted of the presentation of the 108 stimulus images (in random order) for 10 s duration, followed by a variable inter-trial interval (ITI; range 7–13 s; mean of 10 s), which was a dark blank image containing a small white fixation cross at the center (as shown in Fig. 3) . The purpose of inserting the variable ITI was to jitter the onset of stimuli and conditions so that it improves the estimation of the hemodynamic response function (HRF)^68^. During the 10 s that the stimulus images were available to view, participants indicated their decision to either buy or not buy the product using two different buttons on a standard, 4-button, MR-compatible button box (Current Design, Philadelphia, PA). When deciding to buy the presented food item, participant pressed “1”, otherwise they pressed “2” for deciding not to buy the item.

In each experimental session, the 108 stimuli were randomly and evenly divided into three runs for each participant using E-prime software. Each run consisted of 36 images. “Optseq” software was employed to determine the ideal sequence of variable ITIs and trials in this experiment design^69,70^ The sequence generated by Optseq maximized the variance of the predicted fMRI response and minimized the overlap of the hemodynamic response function (HRFs).

##### MRI data acquisition

MRI Data was acquired on a 3T Siemens MAGNETOM scanner (Siemens Healthcare, Erlangen, Germany) using a 32-channel Nova Medical head coil at Auburn University MRI Research Center in Auburn, AL, USA. Functional brain imaging data were acquired using an echo-planner imaging sequence (EPI)^71^ with repetition time (TR) = 1000 ms, echo time (TE) = 30 ms, field of view (FOV) = 24 cm, in-plane resolution = 3 × 3 mm^2^, slice thickness = 5 mm with whole brain coverage. Also, a high-resolution 3D MPRAGE (magnetization-prepared rapid gradient echo) sequence was used to collect T1-weighted structural data for anatomical localization^72^ with the following parameters: TR = 2,300 ms, TE = 3.37 ms, flip angle = 7°, slice thickness = 1 mm, voxel dimension = 1 mm × 1 mm × 1 mm and number of slices = 160. Gradient echo field maps were also acquired for EPI distortion correction.

#### fMRI Data Analysis

##### Preprocessing

Using gradient echo field maps, EPI distortions, including susceptibility artifacts, were corrected using FSL’s FUGUE tools (https://fsl.fmrib.ox.ac.uk/fsl/fslwiki/FUGUE). Several standard image processing steps were performed using statistical parametric mapping (SPM12) software (https://www.fil.ion.ucl.ac.uk/spm/) in MATLAB (version R2016a) environment as follows: motion correction was done to detect and correct for head movements (i.e. realignment using rigid body registration of images using 3 translation and 3 rotation parameters. Default setting in SPM12 were used. In addition, correction for head motion included measurement of framewise displacement and censoring of high motion frames with framewise displacement > 0.2 mm and subsequent interpolation)^73^, normalization was preformed to transform MRI images from native subject space into Montreal Neurological Institute (MNI) standard brain template space using nonlinear warping; spatial smoothing (using a 6 × 6 × 6 Gaussian kernel) was conducted to improve image quality; and finally temporal bandpass filtering (passband: 0.01 to 0.15 Hz) was performed to remove low frequency drift and high frequency noise.

##### Statistical analysis

A general linear model (GLM) was applied to the pre-processed BOLD fMRI data in order to find brain regions activated by conditions of interest. A GLM can be described as the equation below,

2 $$Y = XB + U,$$

where *Y* is the observed fMRI time series, *X* is a design matrix consisting of explanatory variables, *B* is a matrix containing parameters that are to be estimated and *U* is a matrix containing the model error. *X* consisted of time courses expected due to each condition as well as time and dispersion derivative function allowing for variations in subject-subject and voxel-voxel response^74^. The expected time courses were modeled with a boxcar function convolved with the canonical hemodynamic response function (HRF). The boxcar function assumed a value of 1 at times when the subjects saw images corresponding to the condition of interest and a value of zero during other times. Specifically, the conditions of interest were: bought unhealthy food (BUH), bought healthy food control (BHC), bought healthy food low tax (BHT), bought healthy food rebate (BHR), not bought unhealthy food (NUH), not bought healthy food control (NHC), not bought healthy food low tax (NHT), not bought food rebate (NHS). The coefficients of the linear model *B* were then computed as beta-values. Linear contrasts were defined on the columns of the design matrixes, in order to statistically compare the fMRI response to different conditions in every voxel across the brain using t-tests. The voxels which were significantly (*p* < 0.05, FDR corrected, two-sided two-sample t-tests) different between the conditions being compared were displayed as functional activation maps overlaid on the MNI T1-weighted brain template.

For each individual subject, BHR, BHC and BUH were first compared using an ANOVA to find regions significantly different between the conditions. Next, BHT, BHC and BUH were similarly compared using an ANOVA to find regions significantly different between the conditions. Finally, BHR and BHT were directly compared using a two-sample t-test. Once contrast maps were obtained for these contrasts from individual subjects, a second GLM model was fit in order to obtain group level maps for these contrasts. For regions that were significant (p < 0.05 FDR corrected) in the ANOVA, pairwise t-tests were carried out between the three conditions to ascertain whether any two conditions were significantly different from each other. Obtaining thresholded activation maps in individual subjects allowed us to evaluate the consistency of the group activation across individuals. Once voxels consistently activated at the group level as well as individual subjects were determined, it also allowed us to extract subject-level activation estimates only from voxels actually activated in those given subjects.

## Results

### Experiment-1

#### Demographics

Tables 2 and 3 present the demographic characteristics of the participants. The average age of participants was 39 years and average family size was 4 with 2 children (see Table 2). Further, 83% of the participants were female with 80% being African American (see Table 3). Approximately 37% had less than $14,999 annual income, ~ 41% had $15,000–$24,999 annual income and a very small percentage of participants had more than $25,000 annual income. Around 62% were single parents and 22% were married, while the remaining were separated, divorced or widowed. More than 83% were overweight or obese. Most of the participants had some high school or high school degree (59.3%).

#### Choice behavior

**Figure 4:**
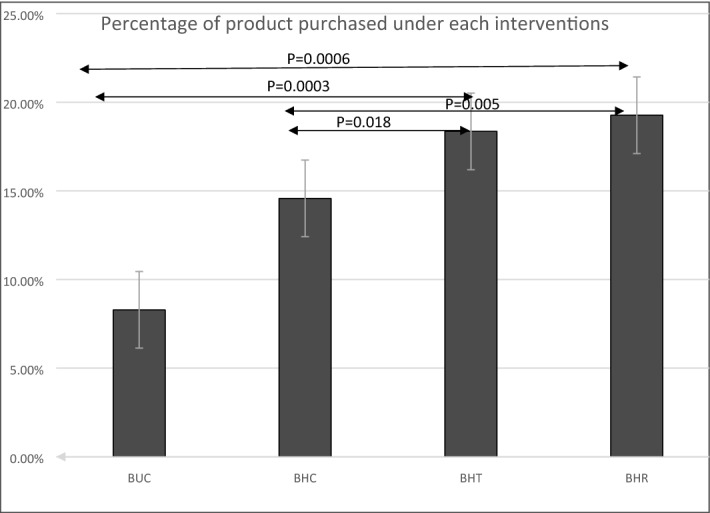
The percentage of products bought under each category across participants in Experiment-1. The percentage was calculated by comparing the number of products that belong to a particular category that were bought compared to the number of products on offer in that category. The percentage of healthy products bought were significantly higher (*p* < 0.05 two-sided two-sample t-tests) when they were associated with lower tax or rebate (BHT and BHR) compared to control conditions, unhealthy control and healthy control (BUC and BHC). BHC: Bought healthy control, BHR: Bought healthy rebate, BHT: Bought healthy lower tax, BUC: Bought unhealthy control.

We restrict our main behavioral analysis to the broad conditions of control, rebate and lower tax for healthy and unhealthy food. Figure 4 presents the overall percentage of products bought under each category across participants. The percentage of healthy products bought were significantly higher (*p* < 0.05, two-sided two-sample t-tests, individual *p*-values shown in Fig. 4) when they were associated with lower tax or rebate (HT and HR) compared to control conditions. Percentage of healthy products bought compared to unhealthy products was higher under the control condition, but the increase in healthy product purchase between the tax or rebate (HT and HR) conditions are significantly larger and greater in size than the control condition.

Table 4 shows the mean characteristics of food purchased within each intervention in terms of calories, added sugar, saturated fat dietary fiber and sodium. It can be seen that for both healthy and unhealthy food items, lower tax and rebate interventions significantly improved the diet by reducing calories (*p* = 0.003), and saturated fat (*p* = 0.000), while simultaneously increasing fiber content in the diet (*p* = 0.004). We did not find significant effects for sugar, sodium and total fat. The corresponding *p*-values obtained using two-sided two sample t-tests are presented in Table 4. Furthermore, we investigated the average change in number of food items purchased under fiscal interventions (control, rebate and lower tax) focusing on individual categories such as fruits, vegetables, grains, etc. These additional results are presented in the appendix in Table A2. Results show that under both treatments healthy food purchase increased significantly for vegetables (*p* = 0.000), grains (*p* = 0.020) and protein (*p* = 0.020), but not for fruits. On the other hand, unhealthy purchase decreased significantly in fruits (*p* = 0.020), dairy (*p* = 0.003), grain (*p* = 0.004), etc. except vegetables. These results combined with results from Table 4 imply that when healthy food items are paired with a rebate or lower tax, they were bought more than when they were offered with regular tax (control) with corresponding improvements in the healthfulness of the diet in terms of significantly decreased calorie (*p* = 0.003) and saturated fat intake (*p* = 0.000) and significantly increased fiber intake (*p* = 0.004). On average, the increase in healthy food purchase was significantly (*p* = 0.020) more for rebate treatment than lower tax treatment (except healthy vegetables). On average, decrease in unhealthy food purchase was more for rebate than lower tax condition for all categories except unhealthy protein and drink.

**Table 4:** Average food purchased within each intervention by category.

Variables	Healthy food					Unhealthy food				
	Control	Low tax	*p* value	Rebate	*p* value	Control	Low tax	*p* value	Rebate	*p*value
Total calories	16,456.1	15,253.9	0.003	8,771.9	0.00	19,182.7	13,699.3	0.001	12,388.8	0.000
Added sugar (grams)	442.2	413.0	0.150	235.8	0.100	1,003.3	689.6	0.051	611.5	0.100
Dietary fiber(grams)	186.4	322.2	0.004	344.2	0.003	128.6	93.6	0.000	83.2	0.000
Saturated fat(gram)	132.6	121.2	0.000	73.5	0.000	253.4	190.9	0.000	177.4	0.000
Sodium(mg)	31.9	29.8	0.01	11.8	0.22	42.2	28.1	0.23	30.1	0.11
Total fat(grams)	74.5	71.4	0.115	54.9	0.17	210.6	161.9	0.21	165.3	0.13

*p* values are presented in the table and we use two-sided two-sample t-tests.

Table 5 presents marginal effects obtained from a logistic regression that predicts the likelihood of healthy/unhealthy foods being purchased as a function of fiscal interventions (low tax, rebate and control) and other individual specific variables. Table 5 shows that under the lower tax treatment, it is 13 (31) times more (less) likely that the participants would purchase healthy (unhealthy) food compared to the control condition. Under the rebate treatment, the participants were 22 (34) times more (less) likely to purchase healthy (unhealthy) food compared to the control condition. All of these effects were statistically significant and corresponding *p*-values are shown in Table 5. We performed the test that the effect of the rebate treatment is significantly different from the lower tax treatment using an F-test of equality of treatments. We found that rebate was significantly different from lower tax with a p-value of 0.0285.

**Table 5:** Marginal Effects obtained from Logistic Regression. The significance of the marginal effects are indicated by corresponding p-values. The magnitude of the marginal effects indicated in the table provide the likelihood of corresponding effects while the sign indicates whether the effects are more or less likely. For example, under the lower tax treatment, it is 13 (31) times more (less) likely that the participants would purchase healthy (unhealthy) food compared to the control condition.

Variables	Healthy	*p-*value	Unhealthy	*p-value*
Lower tax treatment	0. 125**	0.035	−0.312***	0.004
	(0.059)		(0.107)	
Rebate treatment	0. 222***	0.000	−0.347***	0.001
	(0.058)		(0.105)	
Control treatment	−0.031	0.645	−0.097	0.221
	(0.068)		(0.079)	
Demographic Controls	Yes**		Yes**	
Observations	135		135	
Log likelihood	−76.704		−88.101	

***, **, and * denote 99%, 95%, and 90% levels of confidence obtained from a two-sided z-test. Standard errors are shown in the parenthesis. Demographic characteristics (shown in Tables 1, 2, 3) were used as control variables (shown as “yes”). Their effects are presented in a different report (Banerjee et al., *under preparation*).

Next, we estimated the changes in food purchase by individual categories under each fiscal interventions (lower tax and rebate) comparing to the control condition. Tables 6 and 7 present the estimation of coefficients from Eq. (1). Statistical software STATA was used for the data analysis. In Tables 6 and 7, we use ***, **, and * to denote 99%, 95%, and 90% levels of confidence. While Tables 6 and 7 present estimation of coefficients from Eq. (1) after inclusion of demographic variables as additional controls, we also estimated Eq. (1) without demographic controls and found no qualitative change in the results, and these results are thereby not reported.

**Table 6:** Changes in food purchase by individual categories for healthy foods under each fiscal interventions (lower tax and rebate) comparing to the control condition determined by estimation of Eq. 1.

Variables	Healthy	Healthy	Healthy	Healthy	Healthy
	Vegetables	Fruits	Grain	Dairy	Protein
Lower tax treatment	1.328	2.658	1.614***	0.942***	0.993**
	(1.232)	(1.452)	(0.484)	(0.262)	(0.445)
Rebate treatment	1.261	3.480**	1.903***	1.120***	1.038*
	(1.232)	(1.452)	(0.484)	(0.262)	(0.445)
Constant	−5.968	8.148	−3.850*	−2.828**	−2.283
	(4.802)	(5.660)	(1.887)	(1.023)	(1.735)
Demographic Controls	Yes**	Yes**	Yes**	Yes**	Yes**
R-squared	0.172	0.126	0.173	0.212	0.122
Observations	52	52	52	52	52

***, **, and * denote 99%, 95%, and 90% levels of confidence obtained from two sided t-tests. The Table shows mean of estimated coefficients from Eq. 1 and standard errors are shown in the parenthesis. Demographic characteristics (shown in Tables 1, 2, 3) were used as control variables (shown as “yes”). Their effects are presented in a different report (Banerjee et al., under preparation).

**Table 7:** Changes in food purchase by individual categories for unhealthy foods under each fiscal interventions (lower tax and rebate) comparing to the control condition determined by estimation of Eq. 1

Variables	Unhealthy	Unhealthy	Unhealthy	Unhealthy	Unhealthy		Unhealthy	Healthy
	Vegetables	Fruits	Grain	Dairy	Protein		readymade meal	drinks
Lower tax treatment	0.037	−0.137	−1.359**	−0.820**	−0.607		−0.861*	−0.512***
	(0.216)	(0.462)	(0.476)	(0.146)	(0.384)		(0.415)	(0.111)
Rebate treatment	−0.208	−2.448***	−2.137***	−0.064	−0.385		−1.394***	−0.379***
	(0.216)	(0.462)	(0.476)	(0.146)	(0.384)		(0.415)	(0.111)
Constant	−0.989	−2.974	−3.452	−0.401	−3.164*		−2.651	0.996*
	(0.843)	(1.800)	(1.854)	(0.569)	(1.498)		(1.619)	(0.433)
Demographic Controls	Yes**	Yes**	Yes**	Yes**	Yes**		Yes**	Yes**
R-squared	0.177	0.284	0.229	0.100	0.147		0.148	0.163
Observations	52	52	52	52	52		52	52

***, **, and * denote 99%, 95%, and 90% levels of confidence. Two sided t-tests were used. Standard errors are shown in the parenthesis. Unhealthy food was purchased under each intervention (lower tax and rebate) though regular tax rate was applied for unhealthy food under each treatments. Demographic characteristics (shown in Tables 1–3) were used as control variables (shown as “yes”). Their effects are presented in a different report (Banerjee et al., under preparation).

Table 6 presents the estimation of coefficients for healthy food categories. Results show that lower tax treatment increased healthy grain, dairy and protein purchase significantly (*p* = 0.000, *p* = 0.000 and *p* = 0.03, respectively; two sided t-tests were used) compared to the control condition (healthy food with regular tax). Rebate treatment increased fruits, healthy grain, dairy and protein purchase significantly (*p* = 0.02, *p* = 0.000, *p* = 0.000 and *p* = 0.07 respectively; two sided t-tests were used) compared to the control condition. The effect was maximum for fruits (3 units). Table 7 presents the results for unhealthy food categories. Results show that the lower tax treatment decreased the unhealthy grain, dairy, readymade meal and unhealthy drink purchase significantly (*p* = 0.02, *p* = 0.01, *p* = 0.08 and *p* = 0.000, respectively; two sided t-tests were used) compared to the control condition. Table 7 also shows that the rebate treatment decreased the unhealthy food purchase significantly (*p* = 0.000, two sided t-tests were used) in all categories except unhealthy vegetable, dairy, and protein. In addition, the rebate effect was more than the lower tax effect in all food categories except unhealthy drink purchase. For example, lower tax treatment decreased unhealthy grain purchase by 1.3 units whereas the rebate treatment decreased the unhealthy grain purchase by 2.1 units. These results indicate that the lower tax and rebate treatments are effective in changing the food purchase decisions of parents and encourage them to buy more healthy (less unhealthy) food. Further, the rebate treatment is more effective for most of the categories than lower tax treatment in increasing purchase of healthy foods and decreasing purchase of unhealthy foods. Taken together, choice behavior supports our hypotheses enumerated in the introduction and forms the basis for further testing of the brain mechanism underlying the effects of these fiscal interventions.

#### EEG Results

**Figure 5:**
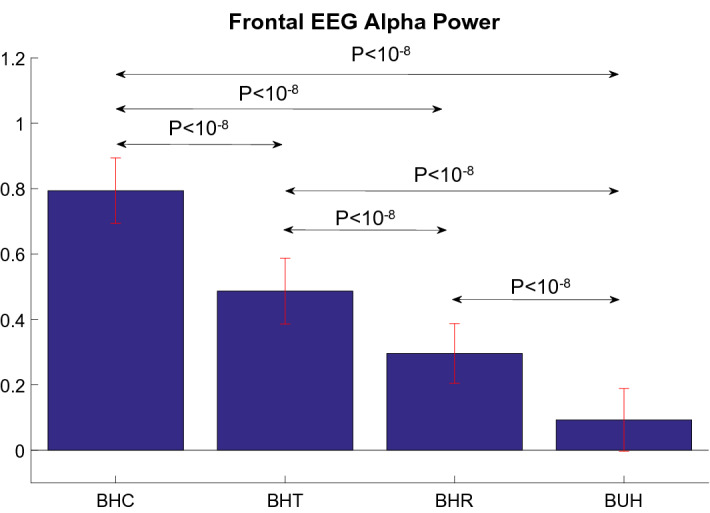
Normalized EEG alpha power estimated from lateral frontal electrodes AF7 and AF8. The bars represent the mean and errorbars represent the standard deviation. The statistical significance of the differences between conditions (obtained from two-sided two-sample t-tests) are also indicated. BHC: bought healthy control, BHR: bought healthy rebate, BHT: bought healthy differential tax, BUH: bought unhealthy.

Figure 5 shows normalized EEG power in the alpha band estimated from lateral frontal electrodes AF7 and AF8 (according to the 10–20 EEG electrode placement system). Although scalp EEG cannot be attributed to underlying cortical sources without performing source localization, we can broadly claim that signal in these electrodes have a dominant contribution from the lateral prefrontal cortices. It can be seen that highest alpha power was obtained for the BHC (bought healthy control) condition followed by BHR (bought healthy rebate) and BHT (bought healthy tax). The BUH (bought unhealthy) elicited least response. Further, the differences in mean power between all pairs of conditions were statistically significant (*p* < 10^–8^, two sample t-test). This result confirms our hypothesis and its implications will be presented in the discussion section.

### Experiment-2

#### In-scanner behavior results

**Figure 6:**
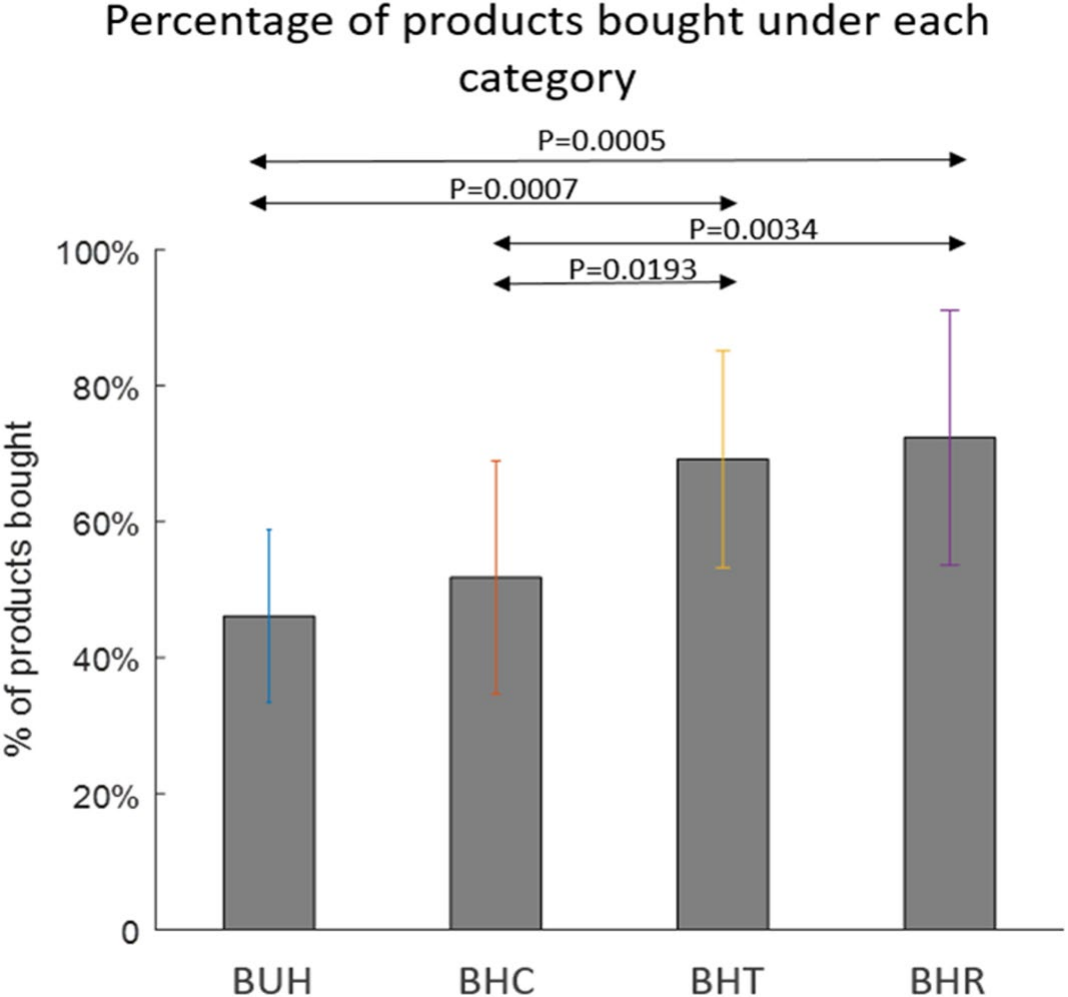
The percentage of products bought under each category across participants in Experiment-2. Since the number of products on offer under each category was different, the percentage was calculated by comparing the number of products that belong to a particular category that were bought compared to the number of products on offer in that category. The percentage of products bought were significantly higher (*p* < 0.05, as indicated) when they were associated with lower tax or rebate (BHT and BHR) compared to control conditions (BUH and BHC). Two-sided two-sample tests were used. BHC: bought healthy control, BHR: bought healthy rebate, BHT: bought healthy tax , BUH: bought unhealthy.

The percentage of products bought under each category is shown in Fig. 6. We found that the percentage of products were significantly (*p* < 0.05 corrected, two-sample t-test) higher when these products were associated with price interventions (lower tax and rebate on healthy foods). However, in terms of the percentage of items bought, the two interventions (rebate and low tax) did not differ significantly (with *p*-value = 0.648), although rebate significantly decreased unhealthy components of the diet in terms of calories, added sugar, saturated fat dietary fiber and sodium, as compared to lower tax condition (as discussed before). It is noteworthy that the absolute value of percentage items bought within each category varies between in-scanner behavior (Fig. 6) and in-store behavior (Fig. 4). This may have been caused by the fact that substantially less number of products were on offer in the fMRI experiments due to constraints of time and experimental design. In addition, contextual factors of the in-scanner and in-store environments may have played a role. Nevertheless, the relative trends obtained from both were the same.

#### Functional MRI activations

**Figure 7:**
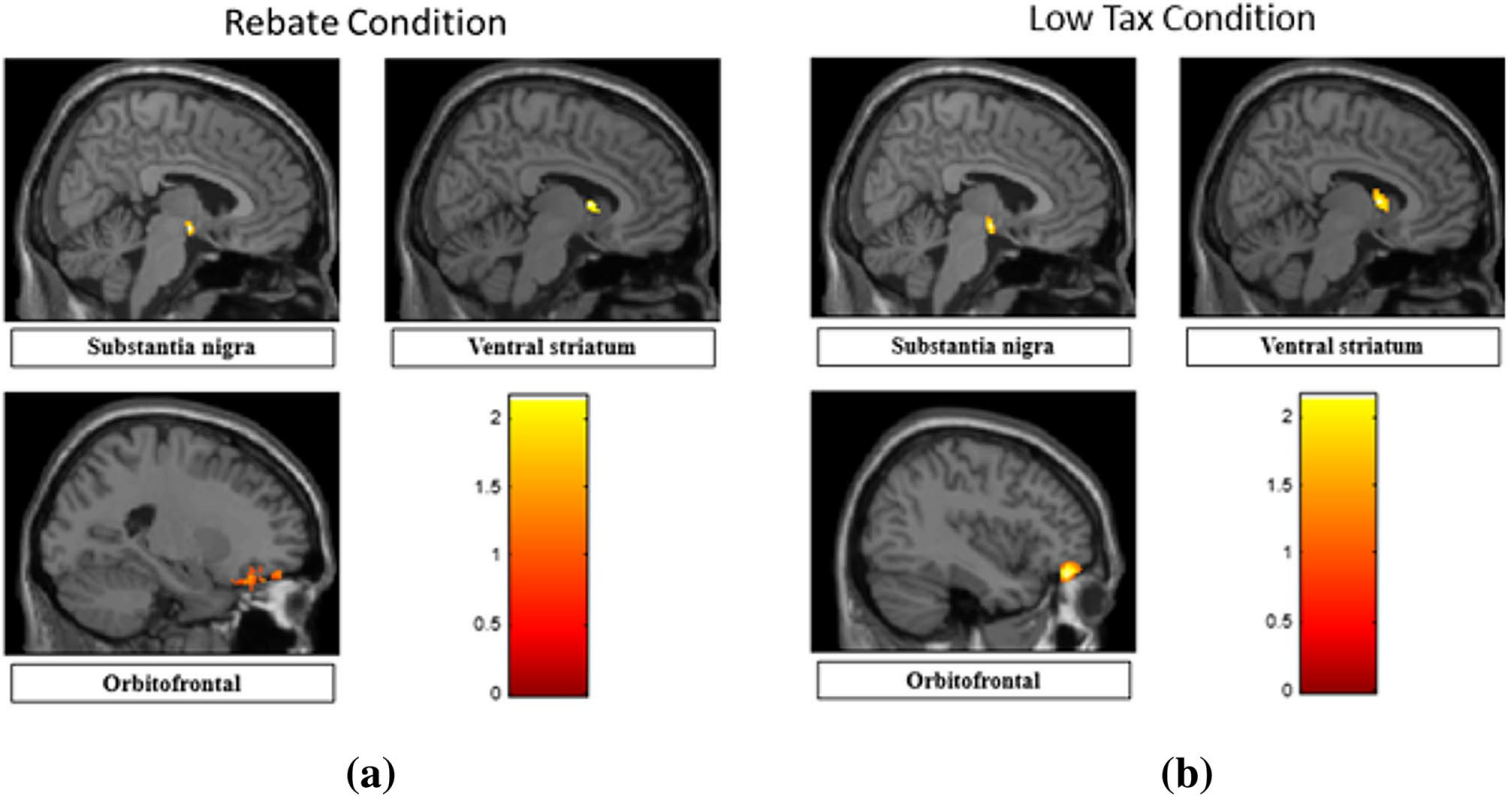
(a) Three brain regions (Substantia nigra, Ventral striatum, Orbitofrontal) which showed significant (*p* < 0.05, FDR corrected, two-sided two-sample t-tests) differences between BHC, BHR and BUH conditions, with highest response to BUH and lowest to BHC. (b) Three brain regions (Substantia nigra, Ventral striatum, Orbitofrontal) which showed significant (*p* < 0.05, FDR corrected, two-sided two-sample t-tests) differences between BHC, BHT and BUH conditions, with highest response to BUH and lowest to BHC. The MNI coordinates of the cluster centroids as well as cluster sizes are shown in Table A3 of the Appendix. BHC: Bought healthy control, BHR: Bought healthy rebate, BHT: Bought healthy lower tax, BUC: Bought unhealthy control.

**Figure 8:**
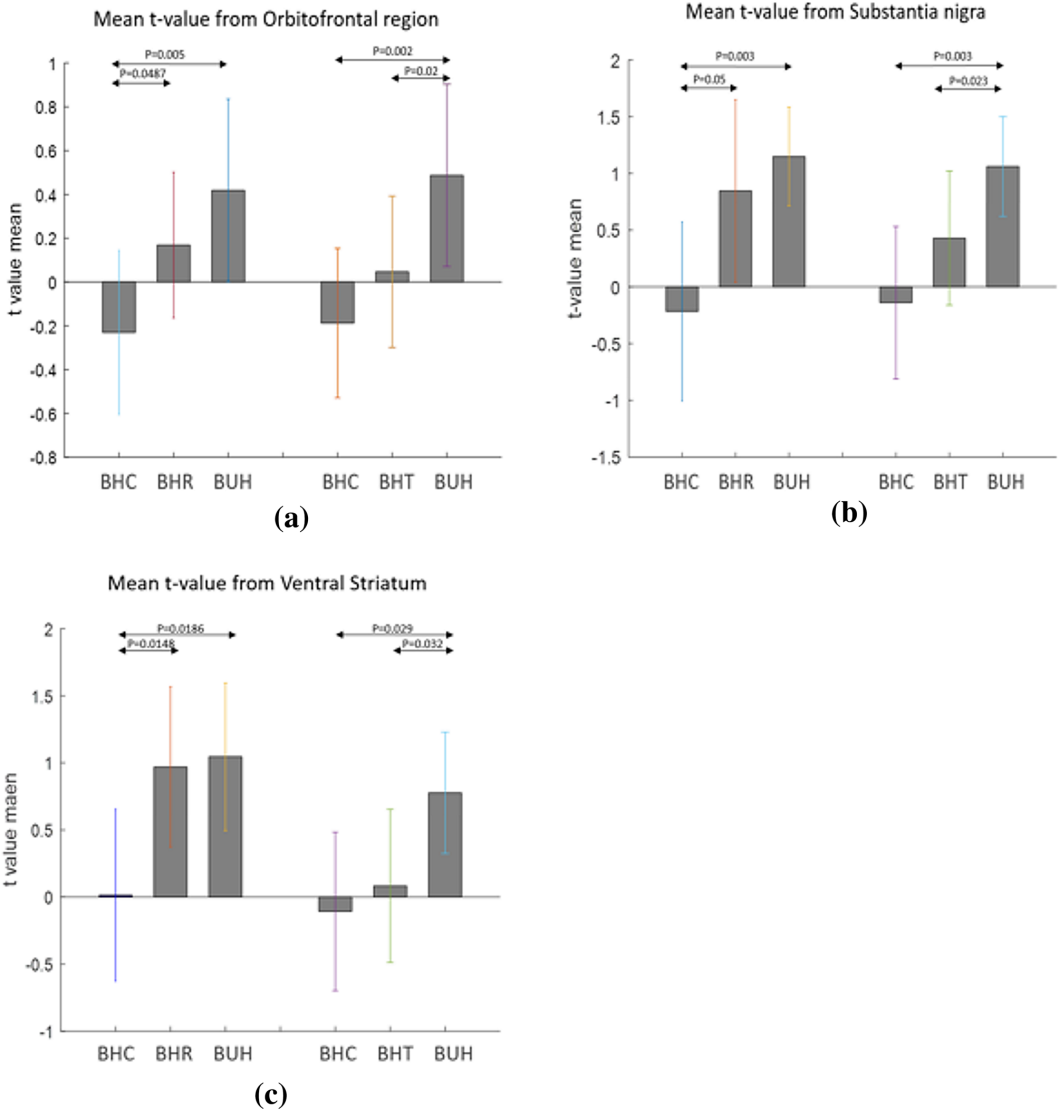
(**a**) The mean t-values extracted from activated voxels in the orbitofrontal region, substantia nigra region and ventral striatum region shown in Fig. 7. For (**a**)–(**c**) bar plots on the left correspond to activated voxels in Fig. 7(a) while those on the right correspond to activated voxels in Fig. 7(b). *P* values obtained from the two-sided two-sample tests used to generate Fig. 7 are also indicated. BHC: Bought healthy control, BHR: Bought healthy rebate, BHT: Bought healthy lower tax, BUC: Bought unhealthy control.

**Figure 9:**
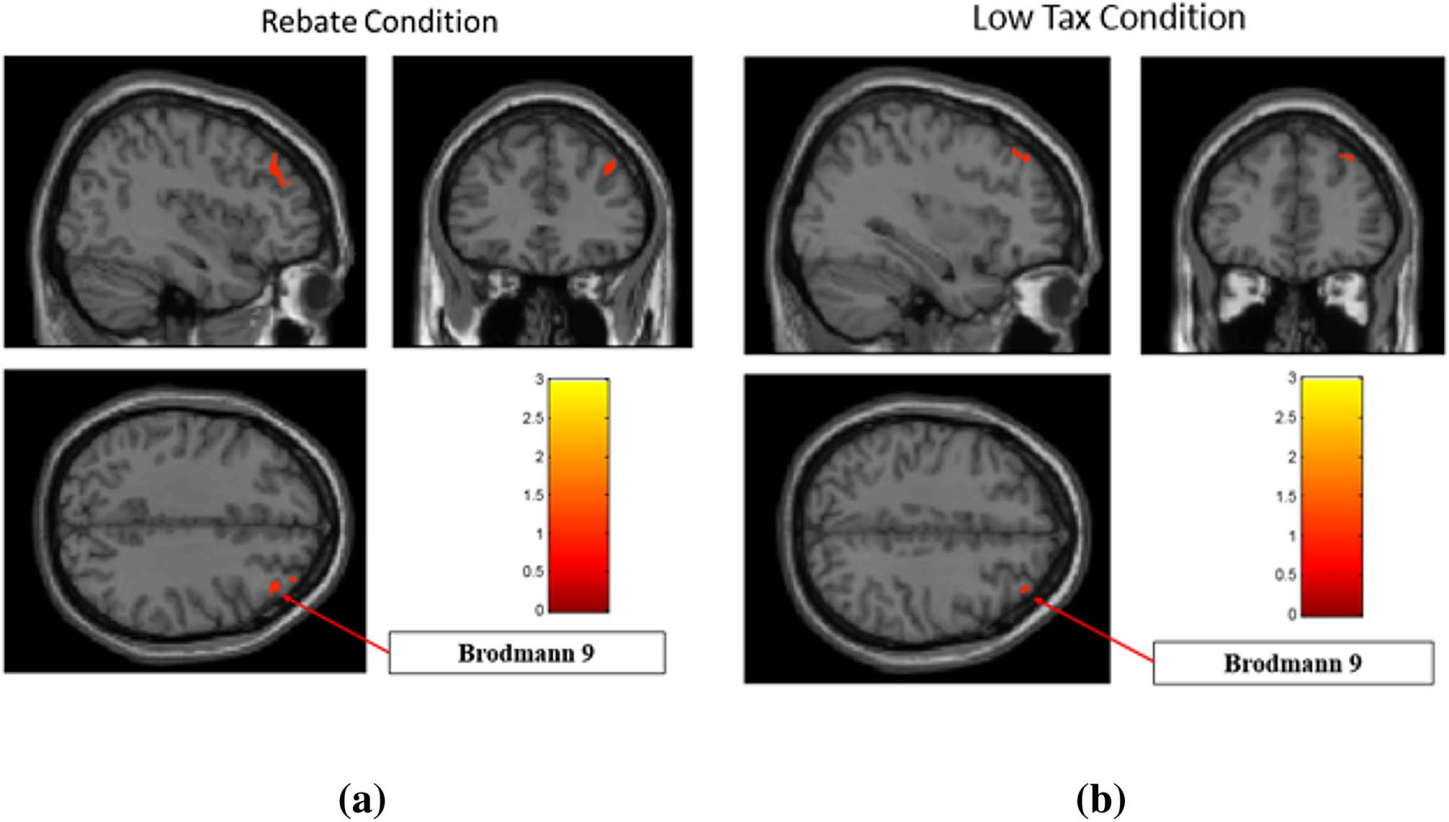
(**a**) The brain region (dorsolateral prefrontal cortex or Brodmann area 9) which showed significant (*p* < 0.05, FDR corrected, two-sided two-sample t-tests) differences between BHC, BHR and BUH conditions, with highest response to BHC and lowest to BUC. (**b**) The brain region (dorsolateral prefrontal cortex or Brodmann area 9) which showed significant (*p* < 0.05, FDR corrected, two-sided two-sample t-tests) differences between BHC, BHT and BUH conditions, with highest response to BHC and lowest to BUH. The MNI coordinates of the cluster centroids as well as cluster sizes are shown in Table A3 of the Appendix. BHC: Bought healthy control, BHR: Bought healthy rebate, BHT: Bought healthy lower tax, BUC: Bought unhealthy control.

**Figure 10:**
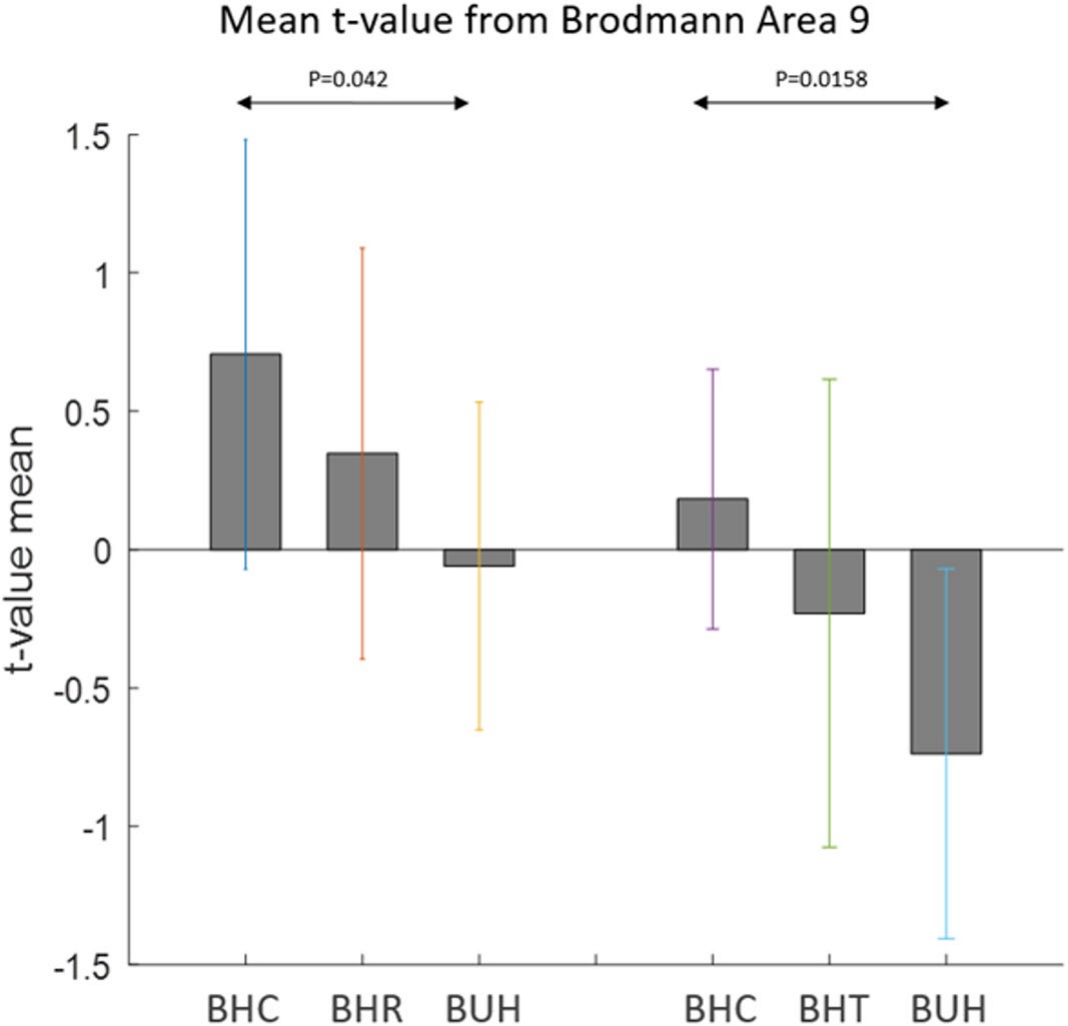
The mean t-values extracted from activated voxels in dorsolateral prefrontal cortex (DLPFC) or Brodmann area 9 shown in Figs. 9. Bar plots on the left correspond to activated voxels in Fig. 9(a) while those on the right correspond to activated voxels in Fig. 9(b). *P* values obtained from the two-sided two-sample tests used to generate Fig. 9 are also indicated. BHC: Bought healthy control, BHR: Bought healthy rebate, BHT: Bought healthy lower tax, BUC: Bought unhealthy control.

**Figure 11:**
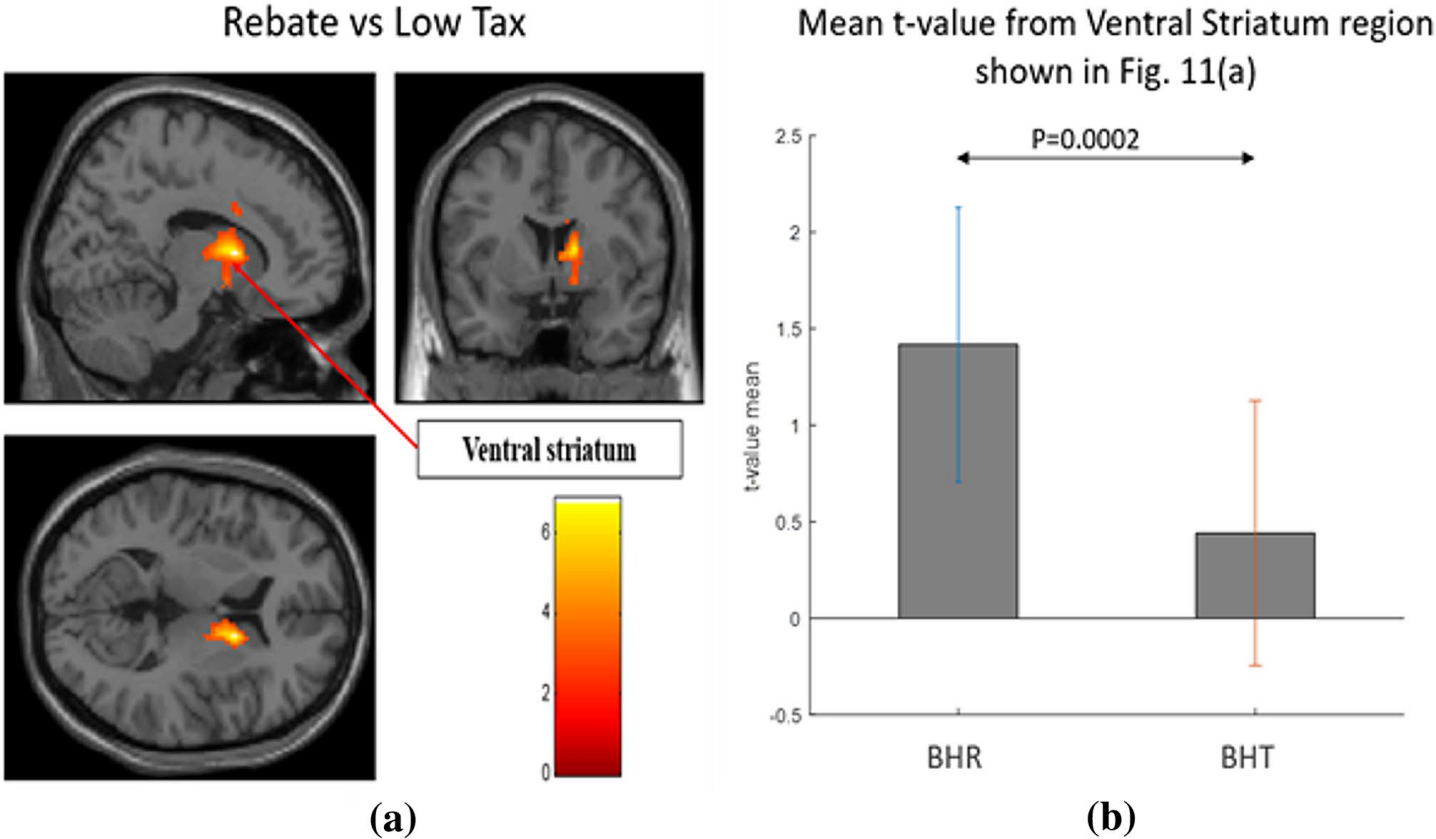
(**a**) The Ventral striatum showed significant (*p* < 0.05, FDR corrected, two-sided two-sample t-tests) differences between BHR and BHT condition. (**b**) The mean t-values extracted from activated voxels in Ventral striatum shown in (**a**). BHC: Bought healthy control, BHR: Bought healthy rebate, BHT: Bought healthy lower tax, BUC: Bought unhealthy control.

For brain regions that were significantly different (*p* < 0.05 FDR corrected) between the three conditions in an ANOVA, two-sided two-sample t-tests were run comparing BHC, BHR and BUH conditions in a pairwise manner. The results are shown separately for regions with highest response to BUH, followed by BHR and lowest to BHC (Fig. 7a), as well as for regions with highest response to BHC, followed by BHR and lowest to BUH (Fig. 9a). Likewise, two-sided two-sample t-tests were run comparing BHC, BHT and BUH conditions in a pairwise manner. The corresponding results are shown separately for regions with highest response to BUH, followed by BHT and lowest to BHC (Fig. 7b), as well as for regions with highest response to BHC, followed by BHT and lowest to BUH (Fig. 9b). Figures 8 and 10 plot the mean t-values obtained from significant regions found in Figs. 7 and 9, respectively. We found that reward-related regions such as ventral striatum, substantia nigra and orbitofrontal cortex (see Figs. 7 and 8) as well as executive control regions such as the dorsolateral prefrontal cortex or Brodmann area 9 (see Figs. 9 and 10) showed significant differences (*p* < 0.05, FDR corrected, two-sided two-sample t-tests) between the fiscal interventions (rebate or lower tax) and control conditions. Specifically, the regions of the reward network showed a pattern of increasing response from BHC to BHR/BHT to BUH conditions as shown in Fig. 8. On the other hand, the activated executive control region of dorsolateral prefrontal cortex (DLPFC) or Brodmann area 9 showed the opposite pattern of decreasing response from BHC to BHR/BHT to BUH conditions as shown in Fig. 10. Also, a direct comparison of the responses under BHR and BHT conditions showed that the ventral striatum (see Fig. 11a) showed significantly (*p* < 0.05, FDR corrected, two-sided two-sample t-tests) greater reward-related activity for rebate compared to lower tax on healthy items (see Fig. 11b).

## Discussion

Functional MRI results demonstrate that the Orbitofrontal, Ventral striatum and Substantia nigra regions have maximal response to BUH condition, followed by BHR (and BHT) and minimal response to BHC condition. In previous studies, all these three regions have been studied and usually defined as regions belonging to the “reward” network in the brain^37,41–44^. Specifically, in food and obesity related research, these regions are thought to encode the calorific reward value in the brain. For example, a study on neural correlates of restrained eaters’ high susceptibility to food cues showed that the Orbitofrontal region was activated by high-energy food cues^42^. Also, Orbitofrontal region was found to have a greater response to high-calorie food versus low-calorie food in a study on emotional eating^43^. Another study investigating the alterations in brain response to food stimuli in overweight and obese individuals, reported that obesity is associated with greater food-evoked responsivity in the ventral striatum^37^. As for the substantia nigra region, previous studies have shown it to be activated with the choice of high energy–density foods^44^. All these previous studies, as well as the extant food choice task fMRI literature^34–36^, shed light on how these regions encode “reward value” in the brain and corroborates our findings. It is noteworthy that there were no differences between conditions in the visual cortex or regions that process color and visual attributes, ruling out any confounding effects of the color coding and other visual attributes associated with fiscal interventions.

Unhealthy foods usually contain higher calories per dollar spent and are perceived to taste better than healthy foods. Hence, people tend to choose unhealthy foods over healthy foods. This is consistent with our results showing that brain regions, which encode calorific reward value showed highest response to unhealthy foods compared to healthy foods in the absence of fiscal interventions. This is reasonable because all individuals have a preference for food that contains more energy for survival, and hence is a preference that is naturally selected during the evolutionary process. In this context, the role of fiscal interventions (rebate or lower tax) is to offset the low reward signal for healthy foods due to their lower calorific value^37,42,43,45–48^, by enhancing perceived reward value for healthy products by associating it with a monetary reward. Many previous studies have shown that the three regions—ventral striatum, substantia nigra and orbitofrontal cortex—which were activated in our task also encode monetary reward value. For example, ventral striatum was showed to be modulated by the magnitude of monetary reward^48^. Another study showed that both substantial nigra and orbitofrontal cortex had a stronger activation when provided with monetary reward than verbal reward^50^. Our results indicate that the rebate or lower tax on healthy products does exactly that, by enhancing the reward value encoded by these regions for healthy food items. We provided a lower tax promotion of 1% tax and rebate of 9% cashback on healthy foods (compared to 10% tax on unhealthy foods) to offset the reward value of unhealthy foods. Therefore, one could envision a scenario where in the amount of tax break or rebate can be titrated to determine the level at which the perceived reward value of healthy food items (through lower tax or rebate) equals or exceeds that of unhealthy food items. This could potentially imply that employing optimal levels of price promotions on healthy foods can consistently lead to choice of healthy over unhealthy foods.

It is noteworthy that the above discussion centers around brain-based responses to healthy and unhealthy food items, all of which were purchased by the participants. In our analysis, we did not consider or compare responses during viewing items, which were not purchased. Therefore, naturally the question arises as to why healthy food items with and without fiscal interventions were bought in spite of lower reward value compared to unhealthy food items. The answer to this question lies in the pattern of responses we found in the dorsolateral prefrontal cortex (DLPFC), specifically in Brodmann area (BA) 9. The responses to BHC, BHR/BHT and BUC in BA 9 followed a pattern exactly opposite to that observed in reward related regions, i.e. it showed maximal response to BHC, followed by BHR/BHT and least response to BUC. BA 9 is an executive control region which exercises top-down cognitive control on decision making^51,52^. Of specific interest is the role of this region in overriding automatic responses generated by bottom-up processes^53^. In our context, this would be to override the bottom-up input from reward-related regions on the calorific reward value of unhealthy foods, and instead opt for healthy foods with lower tax or rebate. Therefore, our results indicate that in order to buy healthy food items without fiscal interventions, BA 9 had to exert maximum cognitive control due to its lowest calorific reward value. In contrast, unhealthy food items already had high calorific reward value and hence needed little cognitive control in order to make a purchase decision. The enhancement of reward value of healthy food items associated with fiscal interventions reduced the amount of cognitive control required by participants to buy them. This is critical in populations with low socioeconomic status because it has been shown that poverty impacts the brain^54^ and its development^75^ and potentially top-down cognitive control mechanisms^76^. Therefore, reducing the amount of cognitive control needed by individuals in such populations for making healthy food choices for themselves and their children is an objective that can be met by titrating the magnitude of price promotions needed.

Our EEG results reinforce the above conclusions from fMRI. This is important because fMRI data was acquired within an artificial and restricted environment whereas EEG data was obtained from the participants while they shopped for groceries in the simulated shopping environment, which is more ecologically valid. Although scalp EEG cannot be attributed to underlying cortical sources without performing source localization, we can broadly claim that signal in these electrodes have a dominant contribution from the lateral prefrontal cortices. Similar to fMRI activations in this area, we observed highest alpha power for the BHC (bought healthy control) condition followed by BHR (bought healthy rebate) and BHT (bought healthy tax) in lateral frontal electrodes AF7 and AF8 (according to the 10–20 EEG electrode placement system). The alpha power from BUH (bought unhealthy) was the least. Further, condition-specific differences were statistically significant. Various previous studies have implicated EEG alpha power for its role in top-down inhibition and cognitive control^55–57^. Therefore, these results independently validate findings from fMRI that healthy and unhealthy food items require highest and lower cognitive control to be purchased, respectively, and that fiscal incentives such as lower tax and rebate can reduce the amount of cognitive control required to purchase a healthy food item.

One important question is which of the two price interventions – rebate or lower tax – is better at achieving those objectives. In our experimental design, for buying same amount of a particular healthy food, participants paid the same net amount money under two price intervention conditions. However, people reacted differently to these two promotions even if there were no difference in the amount of money saved. As discussed in the introduction earlier, many studies in the field of behavioral economics have investigated this aspect^9,26,33^ since rebate and tax breaks are framed differently and that might influence choice behavior. Based on this prior literature, we hypothesized that rebate might be more effective than lower taxes in urging consumers to buy healthy food products. Our results from the behavioral-cum-EEG experiment in the simulated grocery store revealed that rebate was more effective than lower tax for most of the categories in increasing purchase of healthy foods and decreasing purchase of unhealthy foods. Our in-scanner behavioral results indicate that, in terms of the percentage of items bought, these two interventions did not differ significantly (with p-value = 0.648). However, the fMRI responses were significantly greater in the ventral striatum for the rebate condition compared to the lower tax condition, indicating that greater monetary reward value was perceived when the healthy food promotion was framed as a rebate as compared to lowered tax. This provides support for our hypothesis. It is noteworthy that our power analysis as presented in Table 8 indicated that choice behavior has smaller effect sizes as compared to neural responses and this may be one of the reasons why we could not detect differences between rebate and low tax with respect to choice behavior.

**Table 8:** The statistical power obtained from our sample for the observed effects from a post-hoc analysis.

Comparison	Modality	Sample size	Power
Healthy Food: Control Vs. Treatment (1% tax, 9% rebate)	Behavior	52	0.62
	EEG	52	0.99
	fMRI	19	0.80
Healthy Food: 1% tax Vs. 9% rebate	Behavior	52	0.04
	EEG	52	0.97
	fMRI	19	0.89

## Conclusion and limitations

In this paper, we investigated how lower taxes (compared to 10% regular tax) and rebates on healthy food items impact purchase of both healthy and unhealthy food items among low socioeconomic status parents. In addition to measuring choice behavior in a semi-realistic shopping environment, we also investigated corresponding neural correlates using both EEG and fMRI. The behavioral experiment results demonstrate that under the lower tax and rebate treatments, participants bought more healthy food and less unhealthy food compared to the control condition. Specifically, the study demonstrates that compared to the control condition, both fiscal interventions (lowered tax and rebates) improve dietary choices by significantly reducing the purchase of calories, sugar, added sugar, saturated fat, and sodium, while significantly increasing dietary fiber purchased. These results provide preliminary evidence that fiscal interventions tested in this study have the potential to promote healthy food choices among low socioeconomic status households and reduce obesity. However, future studies in the real world are needed to further validate these conclusions.

Some limitations of this study are noteworthy. First, we did not offer different levels of lowered taxes or rebate in order to investigate neural correlates, which parametrically modulate with the amount of these price interventions. However, we did suggest earlier that these levels could be titrated in order to consistently make healthy foods more preferable than unhealthy foods. Second, the sample size of the experiment was not that large given the exploratory nature of the study. Therefore, in order to balance Type I and Type II errors, we employed voxel-wise FDR correction^77^ for multiple comparisons while producing fMRI brain activation results. However, more conservative correction methods such as permutation testing will likely produce even lesser false positives, improving reproducibility^78^. A bigger sample size in future studies will allow for more stringent multiple comparisons correction methods. Larger samples will also allow generalizability of our findings. For example, we found that although the purchase of healthy food increased significantly for rebate and lower tax conditions as compared to the control condition, percentage of products bought was higher for healthy foods in the control condition as compared to unhealthy foods. There is scant literature on this question in the low resource population. Our findings seem to agree with the results reported by Levy et al.^79^ wherein they found that at baseline, without any intervention, low resource patrons in a cafeteria chose more healthy options than unhealthy ones. This may reflect increased awareness in the society about healthy food choices. However, the effects reported by Levy et al.^79^ were not uniform across races. Therefore, future studies with larger samples may be required to confirm the conclusions drawn from this study and test their generalizability across different sections of the society. Third, the study was not conducted in a real grocery store. Due to the logistic issues, we could not arrange a real grocery store study although we provided a semi-realistic setting for testing the hypotheses proposed with adequate controls and high internal validity. While rebates on healthy foods can be implemented in real grocery stores, state intervention is required to offer lowered tax on healthy products. As a first step, we test the proposed hypotheses in a semi-realistic environment so that the evidence-base can be created for implementing more involved changes at the state level. Hence, although the current study tests the hypotheses in a semi-realistic setting, we regard this as a necessary first step for future real-world interventions involving differential taxes for healthy and unhealthy foods. A final limitation is that we did not use real dollars as the purchase medium for the participants. Participants received their compensations at the end of the study. The reason for not using real dollars as the medium of grocery purchase is that it would encourage participants not to reveal their true purchase decision and save the dollar amount because the design of the study allows the participants to save the rebate amount.

## Supplementary information

Supplementary information

## Data Availability

Data and results reported in this work is available upon request.

## References

[1] U.S. Department of Health and Human Services. Overweight and obesity: a major public health issue. Prevention Report. 16, (2001). Accessed on Jan 2016

[2] Hubert HB, Feinleib M, Mc Namara PM, Castelli WP (1983). Obesity as an independent risk factor for cardiovascular disease : a 26-year follow-up of participants in the Framingham Heart Study. Circulation.

[3] Dietz WH (1998). Health consequences of obesity in youth: childhood predictors of adult disease. Pediatrics.

[4] Dietz WH, Robinson TN (2005). Clinical practice: overweight children and adolescents. N. Engl. J. Med..

[5] Satia A. J. Diet related disparities: understanding the problem and accelerating solutions. *J. Am Diet Assoc.* 610–615 (2009). 10.1016/j.jada.2008.12.019 PMC2729116 19328255

[6] Tsai A, Williamson D, Glick A (2011). Direct medical cost of overweight and obesity in the United States: a quantitative systematic review. Obes. Rev..

[7] Roux L, Donaldson C (2004). Economics and obesity: costing the problem or evaluating solutions?. Obes. Res..

[8] Williamson DF, Narayan KM, Teutsch SM (1998). The economic impact of obesity in the United States: whither?. Obes. Res..

[9] Epstein LH, Dearing KK, Roba LG, Finkelstein E (2010). The influence of taxes and subsidies on energy purchased in an experimental purchasing stud. Psychol. Sci..

[10] Faulkner GEJ, Grootendorst P, Nguyen VH, Andreyeva T, Nicitopoulos KA, Christopher A, Cash S, Cawley J, Donnelly P, Drewnowski A, Dubé A, Ferrence A, Janssen J, LaFrance J, Lakdawalla D, Mendelsen R, Powell L, Traill W, Windmeijer F (2011). Economic instruments for obesity prevention: results of a scoping review and modified Delphi survey. Int. J. Behav. Nutr. Phys..

[11] Finkelstein E, French S, Variyam JN, Haines PS (2004). Pros and cons of proposed interventions to promote healthy eating. Am. J. Prev. Med..

[12] Giesen JCAH, Payne CR, Havermans RC, Jansen A (2011). Exploring how calorie information and taxes on high-calorie foods influence lunch decisions. Am. J. Clin. Nutr..

[13] Faith MS, Fontaine KR, Baskin ML, Allison DB (2007). Toward the reduction of population obesity: macrolevel environmental approaches to the problems of food, eating, and obesity. Psychol. Bull..

[14] Kuchler F, Tegene A, Harris JM (2005). taxing snack foods: manipulating diet quality or financing information programs?. Rev. Agric. Econ..

[15] Small taxes on soft drinks and snack foods to promote health | Center for Science in the Public Interest. [Online]. Available: https://cspinet.org/reports/jacobson.pdf. 10.2105/ajph.90.6.854 PMC1446261 10846500

[16] Marshall T (2000). Exploring a fiscal food policy: the case of diet and ischaemic heart disease commentary: alternative nutrition outcomes using a fiscal food policy. BMJ.

[17] Cash, S. B., Sunding, D. L. and Zilberman, D. Fat taxes and thin subsidies: Prices, diet, and health outcomes, Acta Agric. Scand. Sect. C — Food Econ. 2, 3–4, 167–174 (2005).

[18] Maniadakis N, Kapaki V, Damianidi L, Kourlaba G (2013). A systematic review of the effectiveness of taxes on nonalcoholic beverages and high-in-fat foods as a means to prevent obesity trends. Clin. Outcomes Res..

[19] Finkelstein EA, Zhen C, Nonnemaker J, Todd JE (2010). Impact of targeted beverage taxes on higher- and lower-income households. Arch. Intern. Med..

[20] Schroeter C, Lusk J, Tyner W (2008). Determining the impact of food price and income changes on body weight. J. Health Econ..

[21] Pieroni L, Lanari D, Salmasi L (2013). Food prices and overweight patterns in Italy. Eur. J. Heal. Econ..

[22] Chouinard HH, Davis DE, LaFrance JT, Perloff JM (2007). Fat taxes: big money for small change. Forum Health Econ. Policy..

[23] Dharmasena S, Capps O (2012). Intended and unintended consequences of a proposed national tax on sugar-sweetened beverages to combat the US obesity problem. Health Econ..

[24] Gustavsen GW, Rickertsen K (2011). The effects of taxes on purchases of sugar-sweetened carbonated soft drinks: a quantile regression approach. Appl. Econ..

[25] Dharmasena S, Davis GC, Capps O (2014). Partial versus general equilibrium calorie and revenue effects associated with a sugar-sweetened beverage tax. J. Agric. Resour. Econ..

[26] Epstein LH, Dearing KK, Handley EA, Roemmich JN, Paluch RA (2006). Relationship of mother and child food purchases as a function of price: a pilot study. Appetite.

[27] Powell LM, Zhao Z, Wang Y (2009). Food prices and fruit and vegetable consumption among young American adults. Health Place..

[28] Claro RM, Carmo HCE, Machado FMS, Monteiro CA (2007). Income, food prices, and participation of fruit and vegetables in the diet. Rev. Saude Publica..

[29] Powell LM, Chaloupka FJ (2009). Food prices and obesity: evidence and policy implications for taxes and subsidies. Milbank Q..

[30] Kahneman D, Amos T (1979). Prospect theory: an analysis of decision under risk. Econometrica.

[31] Thaler RH (1985). Mental accounting and consumer choice. Mark. Sci..

[32] Jarnebrant, P., Johnson, E., Toubia, O. Small gains or smaller losses: optimal price promotions and the silver lining effect. In *NA—Advances in Consumer Research 34*, Eds. Gavan Fitzsimons and Vicki Morwitz. Duluth, MN : Association for Consumer Research, 18 (2007).

[33] Huettel, S. A., Song, A. W. and McCarthy, G. Functional Magnetic Resonance Imaging. 2008.

[34] Hare TA, Camerer CF, Rangel A (2009). Self-control in decision-making involves modulation of the vmPFC valuation system. Science.

[35] Levy DJ, Glimcher PW (2011). Comparing apples and oranges: using reward-specific and reward-general subjective value representation in the brain. J. Neurosci..

[36] Rangel, A. Regulation of dietary choice by the decision-making circuitry. *Nat. Neurosci.* (2013). 10.1038/nn.3561 PMC4053793 24270272

[37] Verdejo-Román, J., Vilar-López, R., Navas, J. F., Soriano-Mas, C. and Verdejo-García, A. Brain reward system’s alterations in response to food and monetary stimuli in overweight and obese individuals. *Hum. Brain Mapp*. (2016). 10.1002/hbm.23407 PMC6867019 27659185

[38] Eldeghaidy S (2016). Prior consumption of a fat meal in healthy adults modulates the brain’s response to fat. J. Nutr..

[39] Drew Sayer R (2016). Reproducibility assessment of brain responses to visual food stimuli in adults with overweight and obesity. Obesity.

[40] Allen HA (2016). Relationship between parental feeding practices and neural responses to food cues in adolescents. PLoS ONE.

[41] Wise RA (1998). Drug-activation of brain reward pathways”. Drug Alcohol. Depend..

[42] Wang Y (2016). Neural correlates of restrained eaters? High susceptibility to food cues: an fMRI study. Neurosci. Lett..

[43] Wood SMW, Schembre SM, He Q, Engelmann JM, Ames SL, Bechara A (2016). Emotional eating and routine restraint scores are associated with activity in brain regions involved in urge and self-control. Physiol. Behav..

[44] Fearnbach SN (2016). Brain response to images of food varying in energy density is associated with body composition in 7- to 10-year-old children: results of an exploratory study. Physiol. Behav..

[45] Murdaugh LD, James EC, Edwin WC, Rosalyn EW (2012). fMRI reactivity to high-calorie food pictures predicts short- and long-term outcome in a weight-loss program. NeuroImage.

[46] Stoeckel LE, Weller RE, Cook EW, Twieg DB, Knowlton RC, Cox JE (2008). Widespread reward-system activation in obese women in response to pictures of high-calorie foods. NeuroImage..

[47] Yvonne R, Claudia P, Georg B, Hans-Christian B, Randolf K, Herta F, Burghard FK (2007). Differential activation of the dorsal striatum by high-calorie visual food stimuli in obese individuals. NeuroImage..

[48] Anastasia D, Jean T, Alan H, James K (2012). Greater corticolimbic activation to high-calorie food cues after eating in obese vs. normal-weight adults. Appetite..

[49] Rosell-Negre, P., Bustamante, J. C., Fuentes-Claramonte, P., Costumero, V., Benabarre, S. and Barrós-Loscertales, A. Monetary reward magnitude effects on behavior and brain function during goal-directed behavior. *Brain Imaging Behav*. 1–13 (2016). 10.1007/s11682-016-9577-7 27473167

[50] Kirsch P (2003). Anticipation of reward in a nonaversive differential conditioning paradigm and the brain reward system: an event-related fMRI study. Neuroimage..

[51] Kuss K, Falk A, Trautner P, Montag C, Weber B, Fliessbach K (2015). Neuronal correlates of social decision making are influenced by social value orientationâ€”an fMRI study. Front. Behav. Neurosci..

[52] Deppe M, Schwindt W, Kugel H, Plassmann H, Kenning P (2005). Nonlinear responses within the medial prefrontal cortex reveal when specific implicit information influences economic decision making. J. Neuroimaging.

[53] Kübler A, Dixon V, Garavan H (2006). Automaticity and reestablishment of executive control-an fMRI study. J. Cognit. Neurosci..

[54] Noble KG (2015). Family income, parental education and brain structure in children and adolescents. Nat Neurosci..

[55] Sauseng P, Klimesch W, Doppelmayr M, Pecherstorfer T, Freunberger R, Hanslmayr S (2005). EEG alpha synchronization and functional coupling during top-down processing in a working memory task. Hum. Brain Mapp..

[56] Cooper NR, Croft RJ, Dominey SJJ, Burgess AP, Gruzelier JH (2003). Paradox lost? Exploring the role of alpha oscillations during externally vs. internally directed attention and the implications for idling and inhibition hypotheses. Int. J. Psychophysiol..

[57] Klimesch W, Sauseng P, Hanslmayr S (2007). EEG alpha oscillations: the inhibition-timing hypothesis. Brain Res. Rev..

[58] National Center for Chronic Disease Prevention and Health Promotion | Division of Population Health, “CDC - BRFSS,” Centers for Disease Control and Prevention, 2015. [Online]. Available: https://www.cdc.gov/brfss/.

[59] Report MW (2014). Youth risk behavior surveillance—United States. Surveill. Summ..

[60] According to the data presented by University of Washington’s Institute for Health Metrics and Evaluation. https://www.healthdata.org/research-article/prevalence-physical-activity-and-obesity-us-counties-2001–2011-road-map-action.

[61] https://www.cnpp.usda.gov/DietaryGuidelines. Accessed on Jan 2016.

[62] https://www.sale-tax.com/. Accessed on Jan 2016.

[63] https://www.neuroelectrics.com/. Accessed on Jan 2016.

[64] Greene, W. H. Econometric Analysis (Seventh ed.). Upper Saddle River: Pearson Prentice-Hall. pp. 332–344. ISBN 978-0-273-75356-8. (2012).

[65] Data Analysis with NIC Offline - Neuroelectric’s Wiki.” [Online]. Available: https://wiki.neuroelectrics.com/index.php/Data_Analysis_with_NIC_Offline. Accessed: 24 Mar 2017.

[66] Brain Products GmbH / Downloads / Tutorials &amp; SetUp Poster. [Online]. Available: https://www.brainproducts.com/downloads.php?kid=21&tab=1. Accessed: 24 Mar 2017.

[67] Brain Products GmbH / Downloads / Tutorials &amp; SetUp Poster.” [Online]. Available: https://www.brainproducts.com/downloads.php?kid=21&tab=1. Accessed: 24 Mar 2017.

[68] Casanova R (2009). Hemodynamic response function : a comparative analysis. Sci. York.

[69] Optseq Home Page.[Online]. Available: https://surfer.nmr.mgh.harvard.edu/optseq/.

[70] Neely MN, Walter EJ, Black M, Reiss AL (2012). Neural correlates of humor detection and appreciation in children. J. Neurosci..

[71] Poustchi-Amin M, Mirowitz SA, Brown JJ, McKinstry RC, Li T, Technology T (2001). Principles and applications of echo-planar imaging: a review for the general radiologist. Radiographics.

[72] Wang J, Zheng L, He H, Lu ZL (2014). Optimizing the magnetization-prepared rapid gradient-echo (MP-RAGE) sequence. PLoS ONE.

[73] Roalf DR, Quarmley M, Elliott MA, Satterthwaite TD, Vandekar SN, Ruparel K, Gennatas ED, Calkins ME, Moore TM, Hopson R, Prabhakaran K, Jackson CT, Verma R, Hakonarson H, Gur RC, Gur RE (2016). The impact of quality assurance assessment on diffusion tensor imaging outcomes in a large-scale population-based cohort. NeuroImage..

[74] Gitelman DR (2015). Convolution models for FMRI. Brain Mapp.: Encycl. Ref..

[75] Hair NL, Hanson JLB, Wolfe L, Pollak SD (2015). Association of child poverty, brain development, and academic achievement. JAMA Pediatr..

[76] Angiulli AD, Lipina SJ, Olesinska A (2012). Explicit and implicit issues in the developmental cognitive neuroscience of social inequality. Front. Hum. Neurosci..

[77] Han H, Glenn AL (2018). Evaluating methods of correcting for multiple comparisons implemented in SPM12 in social neuroscience fMRI studies: an example from moral psychology. Soc. Neurosci..

[78] Eklund A, Nichols TE, Knuttson H (2016). Cluster failure: Why fMRI inferences for spatial extent have inflated false-positive rates. Proc. Natl. Acad. Sci. USA.

[79] Levy DE, Riis J, Sonnenberg LM, Barraclough SJ, Thorndike AN (2012). Food choices of minority and low-income employees: a cafeteria intervention. Am. J. Prev. Med..

