# Supplementary material for: A neurobehavioral study on the efficacy of price interventions in promoting healthy food choices among low socioeconomic families
Source: Sci Rep. 2020 Sep 22;10:15435. doi: 10.1038/s41598-020-71082-y (PMC7508865; doi:10.1038/s41598-020-71082-y)
Supplement: Supplementary file 1 — Supplementary information [file 41598_2020_71082_MOESM1_ESM.pdf]

A Neurobehavioral Study on the Efficacy of Price Interventions in Promoting Healthy Food Choices among Low Socioeconomic Families

Tannista Banerjee[1+], Veena Chattaraman[2,6], Hao Zou[3] and Gopikrishna Deshpande[3,4,5,6,7,8,9,10]*

[1]*Department of Economics, Auburn University, Auburn, AL, USA*

[2]*Department of Consumer and Design Sciences, Auburn University, Auburn, AL, USA*

[3]*AU MRI Research Center, Department of Electrical and Computer Engineering, Auburn University, Auburn, AL, USA*

[4]*Department of Psychology, Auburn University, Auburn, AL, USA*

[5]*Alabama Advanced Imaging Consortium, Auburn University and University of Alabama Birmingham, AL, USA*

[6]*Center for Health Ecology and Equity Research, Auburn University, Auburn, AL, USA*

[7]*Center for Neuroscience, Auburn University, Auburn, AL, USA*

[8] *School of Psychology, Capital Normal University, Beijing, China,*

[9] *Key Laboratory for Learning and Cognition, Capital Normal University, Beijing, China*

[10] *Department of Psychiatry, National Institute of Mental Health and Neurosciences, Bangalore, India*

Appendix

Table A1: *List of food items used in the behavioral study*

**Healthy food Items**
Carrots, raw
Broccoli, raw
Asparagus, raw
Bell pepper, raw
Beans, raw
Lettuce, raw
White potato, raw
Orange, raw
Peach, raw

Pear, raw
Pineapple, raw
Orange juice, 100% pure, canned, bottled, or in a carton
Apple, raw
Banana, raw
Oatmeal, cooked, regular, no fat added (100% natural whole grain, no sugar)
Cheerios (Whole grain)
Bread, multigrain
Brown rice
Wheat crackers
Popcorn, butterless
baked chips, low fat
Milk, cow’s, fluid, 2% fat
Yogurt, w/fruit, nonfat milk, low-cal sweetener
Eggs
Dark Chocolate
Beef, roast, roasted, lean only
Pork chop, broiled or baked, lean only
Chicken, breast, roasted/broiled/baked, w/o skin
Peanut butter, low fat
Water Bottles

**Unhealthy Food Items**
Pinto, calico/red/Mex. beans; dry, cooked, fat added
Pinto, calico/red/Mex. beans; dry, cooked, fat added
White potato, french fries, (frozen), deep-fried
Canned oranges in syrup
Peach, cooked or canned, in light or medium syrup
Pear, canned, in syrup
Pineapple, canned, in syrup
Orange juice from concentrate, sugar added
White bread
White rice
Oatmeal, with added sugar
Chocolate Cheerios with added sugar
Ice cream sandwich
Roll, sweet, cinnamon bun, frosted
Cookie, chocolate chip
Popcorn, popped in oil, buttered
Potato chips
Frosted flakes, Kellogg
Milk, cow’s, fluid, full fat
Yogurt, fruit variety
Milk chocolate
Fried steak

Chicken, breaded and fried
Chicken patty/fillet/tenders, breaded, cooked
Pizza w/meat, thin crust
Spaghetti w/tomato sauce & meat sauce
Macaroni or noodles w/cheese
Pork & beans
Ice cream, regular, not chocolate
Soft drink, cola-type

Table A2: *The average change in number of food items purchased for rebate and low tax treatments with respect to the control condition, i.e. healthy foods with regular tax, for individual categories. The p-values obtained from two-sided t-tests are also shown for each comparison. The presented numbers are coefficients of the model in Eq.1 where positive/negative coefficients indicate increase/decrease in number of corresponding category of food items purchased, respectively.*

Treatment	Rebate	*P* value	Low tax	*P* value
Change in healthy vegetables	0.865***	0.000	1.712***	0.000
Change in healthy fruit	3.481	0.802	1.962	0.200
Change in healthy grain	2.019**	0.020	1.635**	0.019
Change in healthy dairy	1.112	0.278	1.019***	0.000
Change in healthy protein	1.327**	0.020	1.173**	0.029
Change in unhealthy vegetables	-0.288	0.300	-0.135	0.139
Change in unhealthy fruit	-2.769	0.051	-0.404**	0.030
Change in unhealthy grain	-2.153	0.100	-1.519***	0.001
Change in unhealthy dairy	-0.115**	0.020	-0.076**	0.016
Change in unhealthy protein	-0.653***	0.003	-0.807***	0.004
Change in unhealthy readymade meal	-1.557**	0.040	-0.980***	0.004
Change in total unhealthy drinks	-0.153**	0.029	-0.211***	0.002
Observations	52		52	

***, **, and * denote 99%, 95%, and 90% levels of confidence.

Table A3: *The MNI coordinates of the cluster centroids as well as cluster sizes of the regions shown in Figs 7, 9 and 11*

Comparison	Region	MNI coordinates (x,y,z) of cluster centroids	Number of voxels in cluster
BHC, BHR and BUH	Substantia Nigra	6.00, -5.60, -9.80	31
	Ventral Striatum	8.00, 6.00, 4.00	20
	Left Orbitofrontal	-22.00, 50.00, -18.00	487
	BA 9	34.00, 45.70, 34.30	103
BHC, BHT and BUH	Substantia Nigra	6.00, -6.00, -10.00	31
	Ventral Striatum	8.00, 6.00, 6.00	58
	Left Orbitofrontal	-38.90, 44.80, -18.00	66
	BA 9	32.00, 38.00, 46.00	41
BHT and BHR	Ventral Striatum	14.00, 4.50, 8.50	458

BHC: Bought healthy control, BHR: Bought healthy rebate, BHT: Bought healthy lower tax, BUC: Bought unhealthy control.

Appendix 1

PAY ONLY 1% TAX ON SELECTED ITEMS!

*APPLY TO ALL THE ITEMS WITH GREEN PRICE LABELS!

PAY ONLY 1% TAX

CARROTS
$0.88 /LB

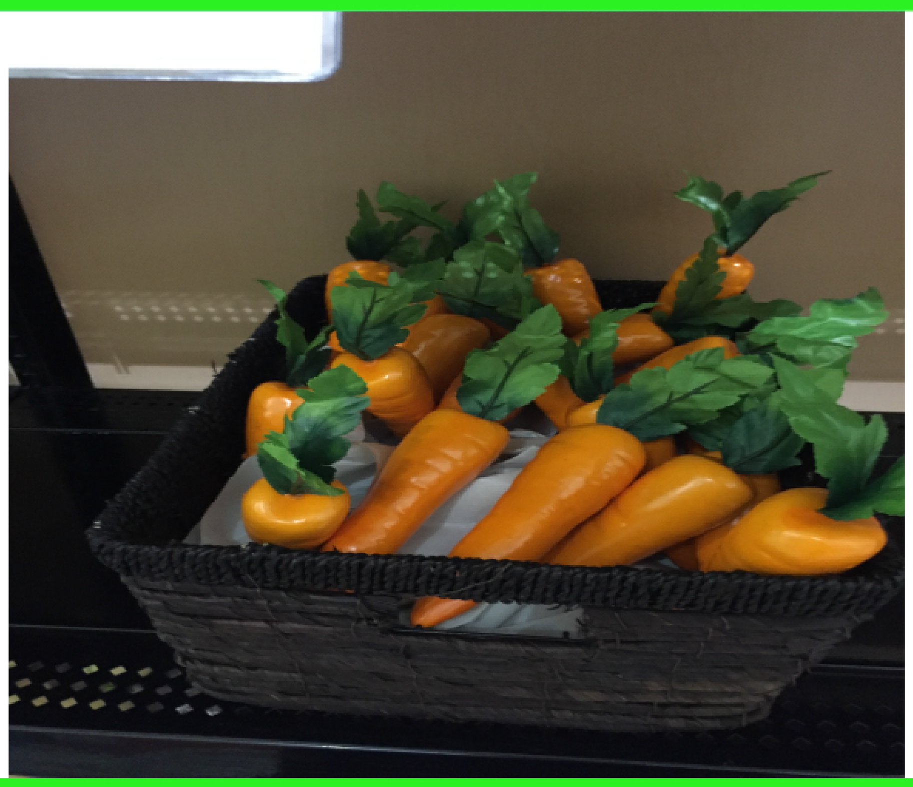

PAY ONLY 1% TAX

BROCCOLI
$2.37 EA

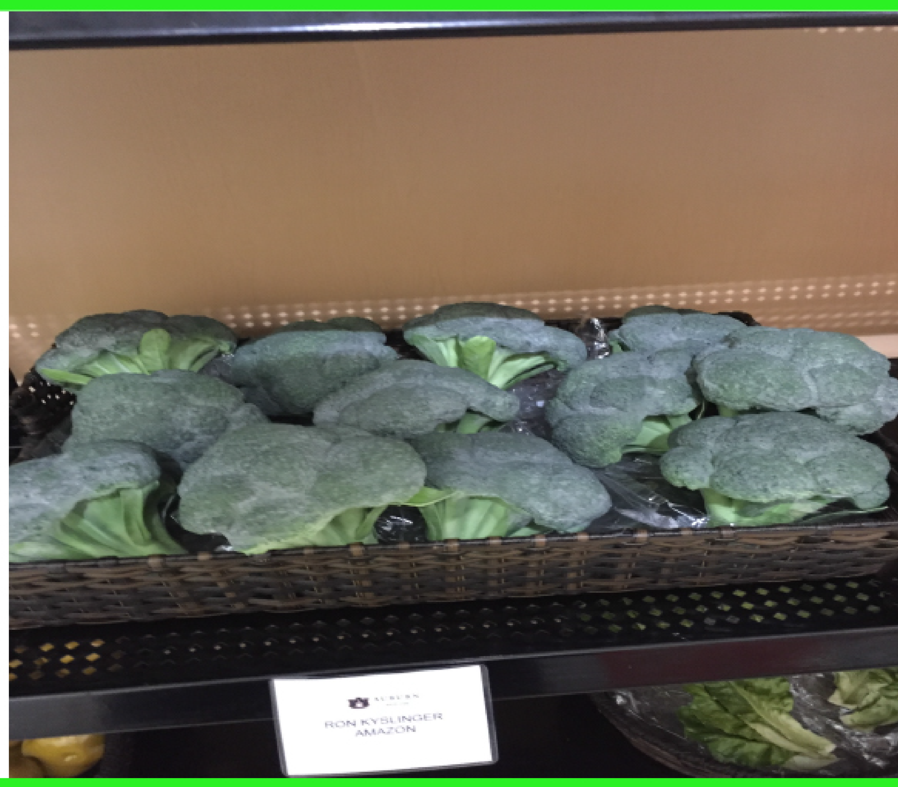

PAY ONLY 1% TAX

ASPATAGUS
$4.63 /lb

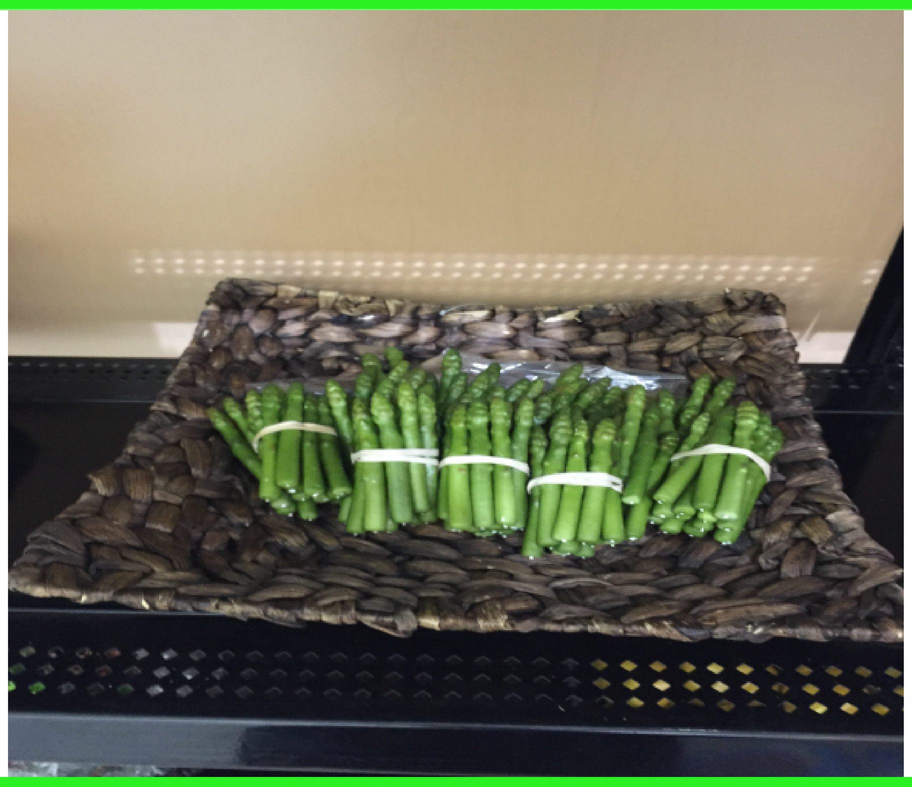

PAY ONLY 1% TAX

BELL PEPPER
$1.65 /lb

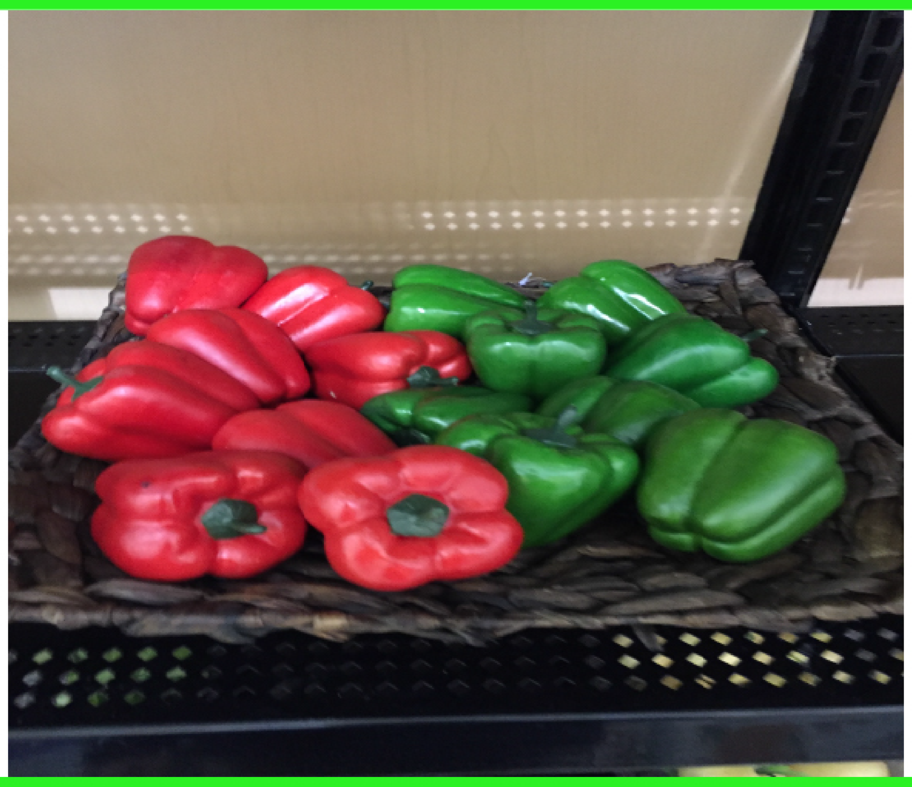

PAY ONLY 1% TAX

GREEN BEANS
$1.45 /lb

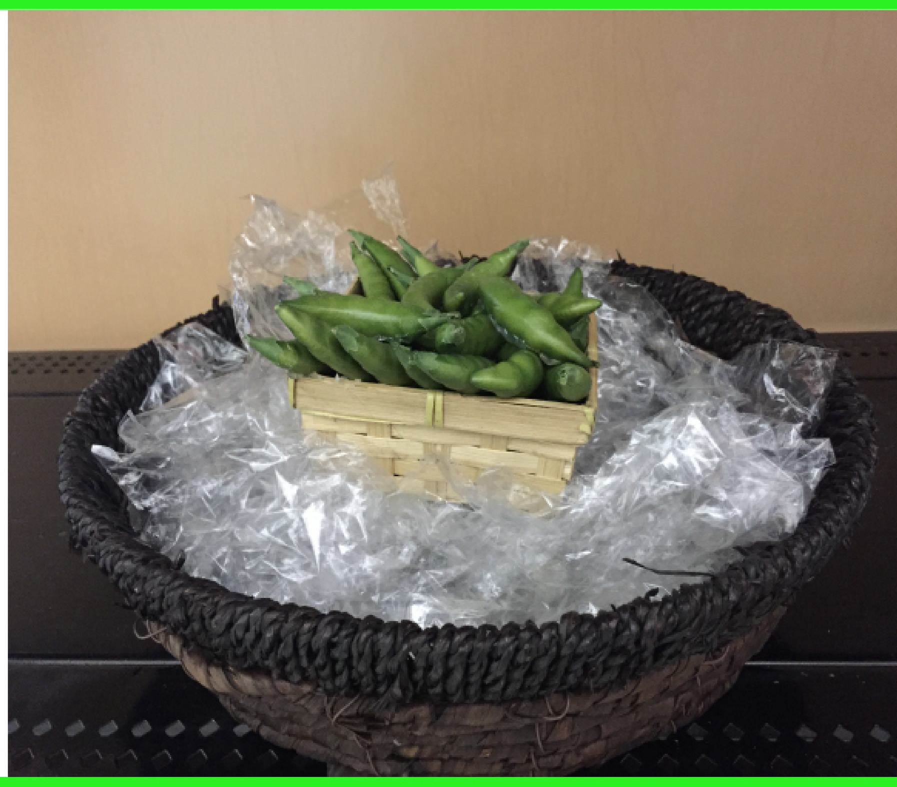

PAY ONLY 1% TAX

LETTUCE
$1.25 /LB

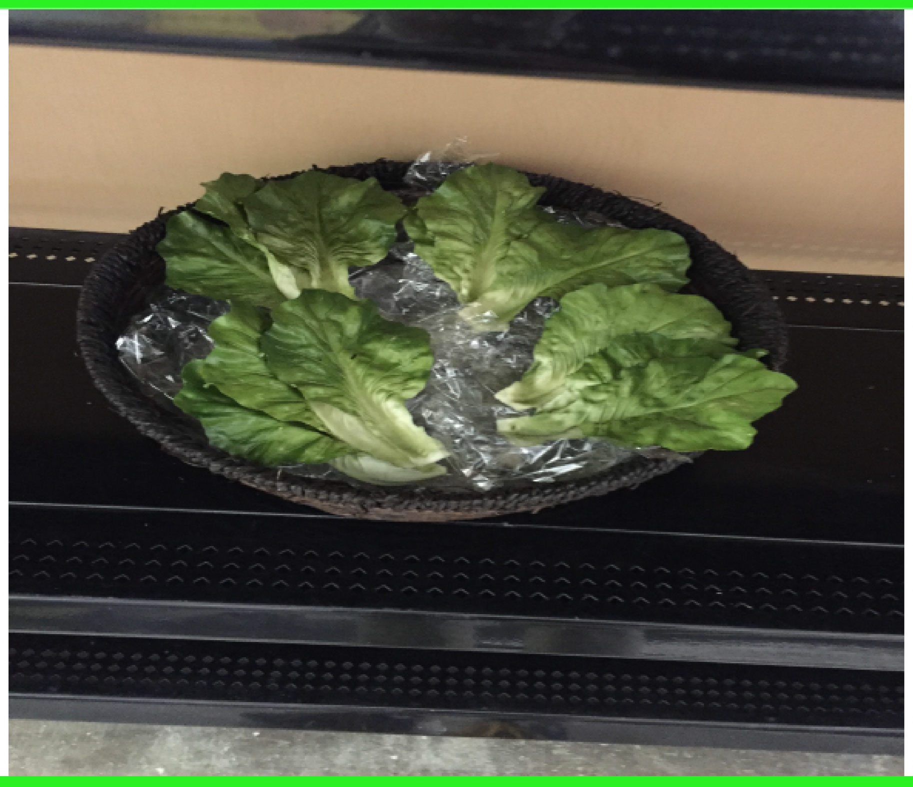

PAY ONLY 1% TAX

WHITE POTATO
$1.10 /LB

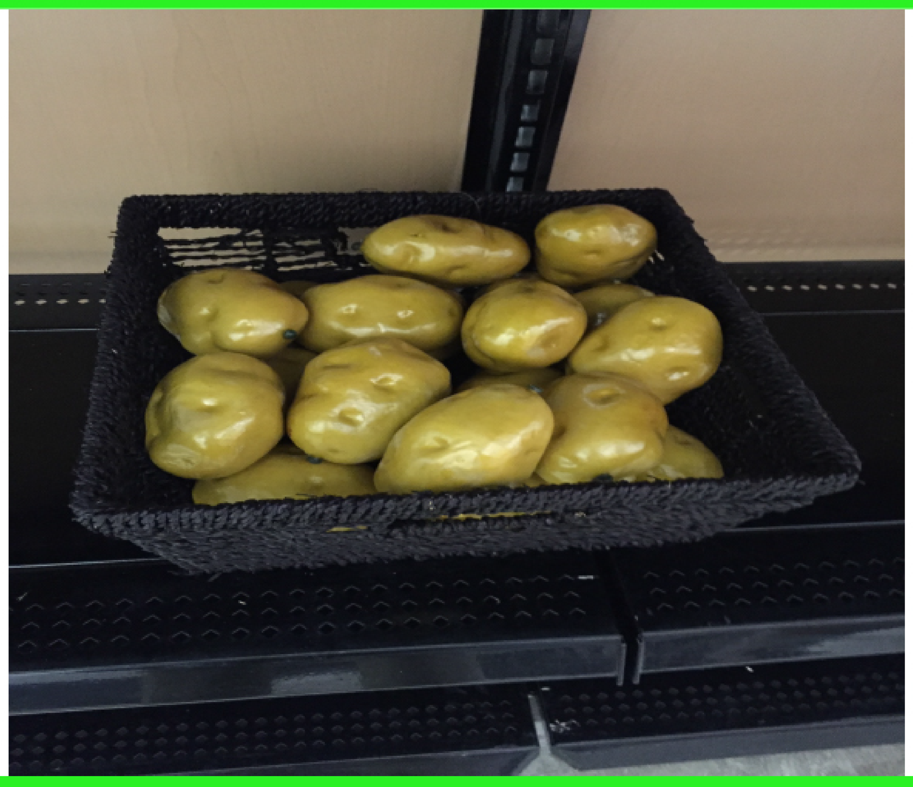

PAY ONLY 1% TAX

PEACH
$2.49 /LB

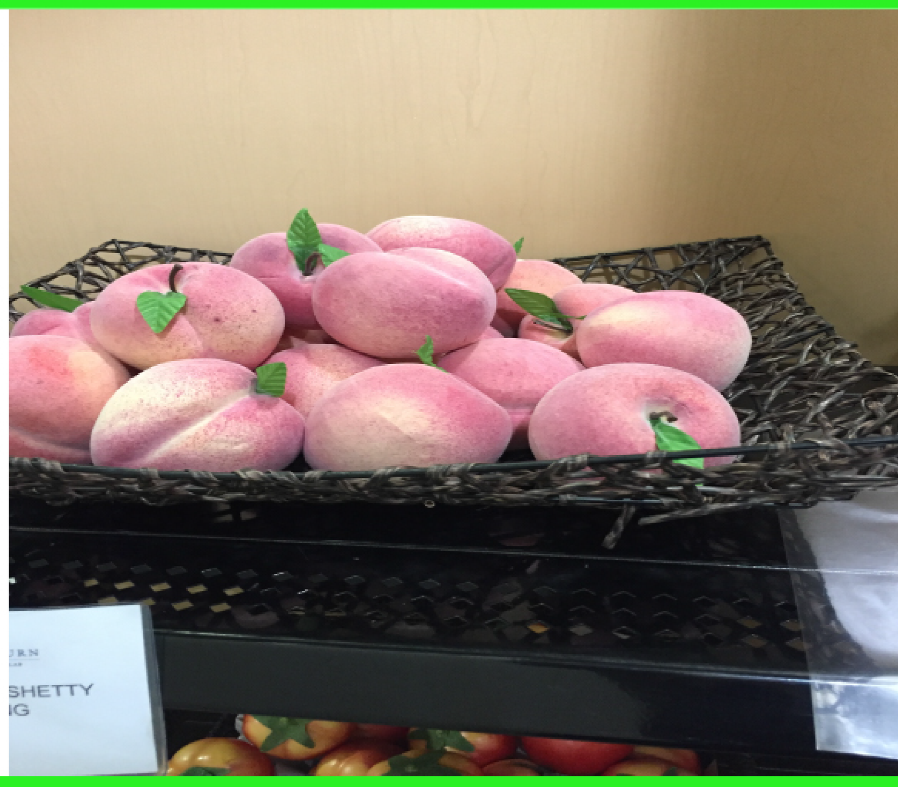

PAY ONLY 1% TAX

ORANGE
$0.80 EA

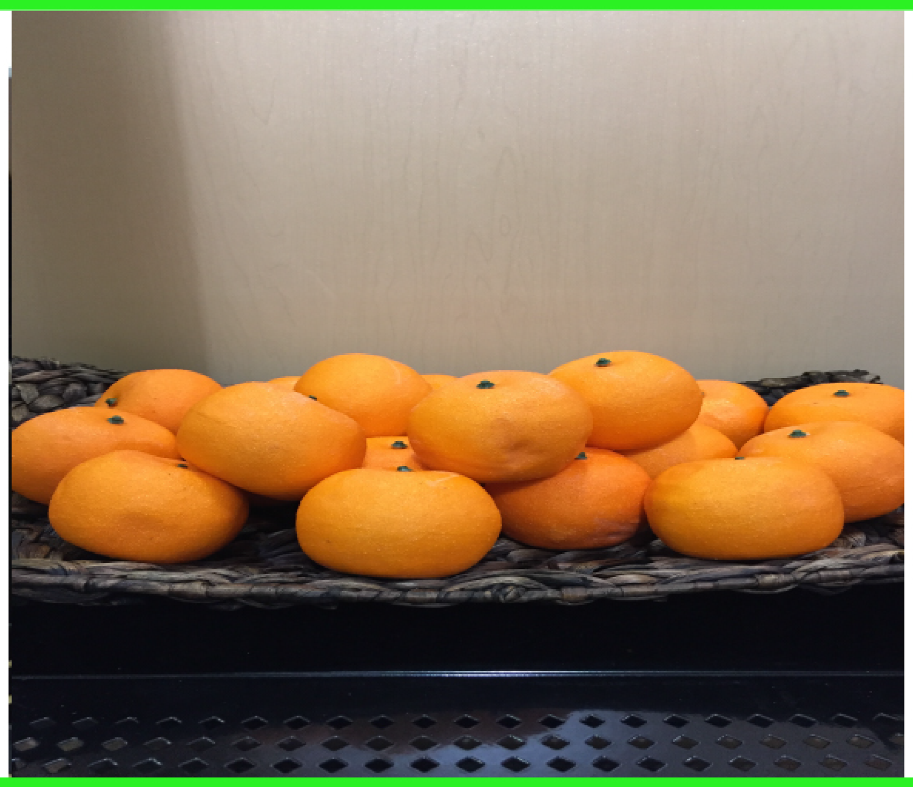

PAY ONLY 1% TAX

PEAR
$2.40 /LB

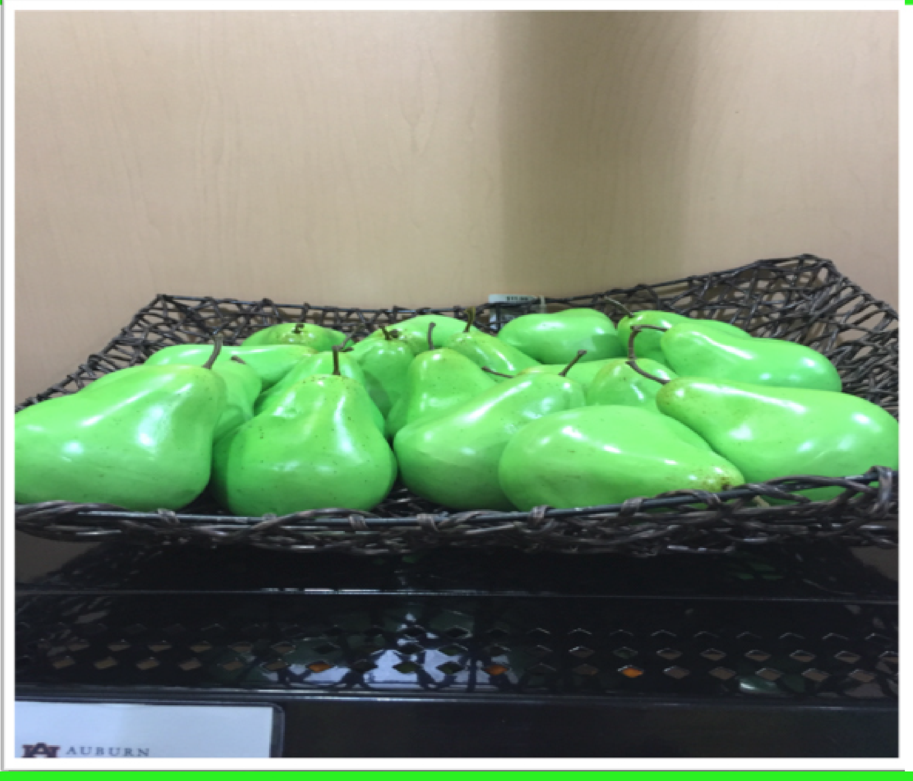

PAY ONLY 1% TAX

PINEAPPLE
$2.80 /EA

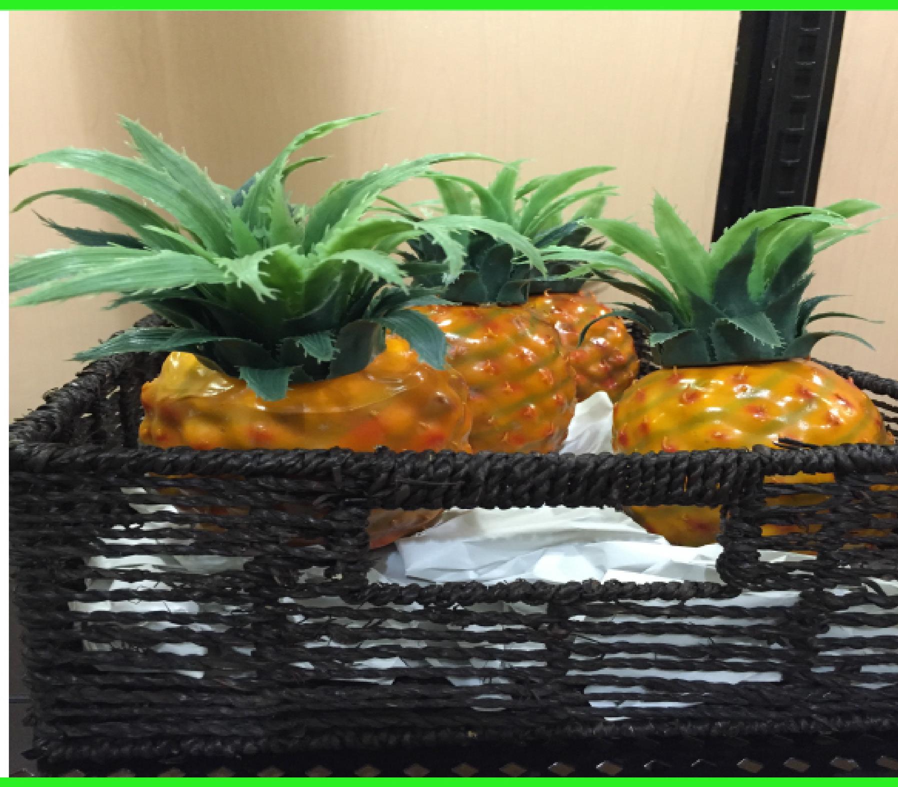

PAY ONLY 1% TAX

APPLE
$2.00 /LB

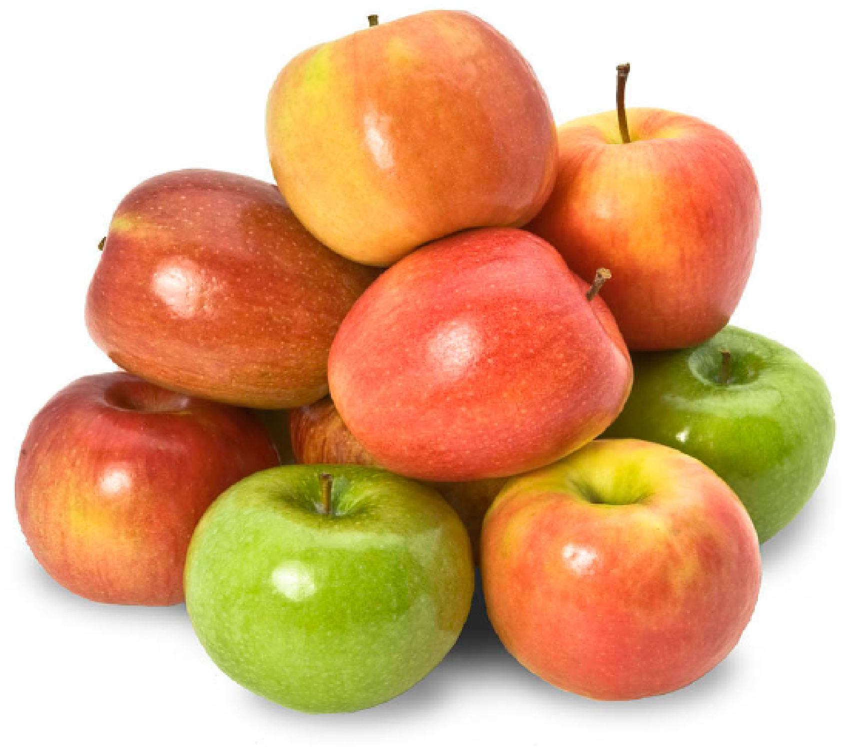

PAY ONLY 1% TAX

BANANA
$0.59 /LB

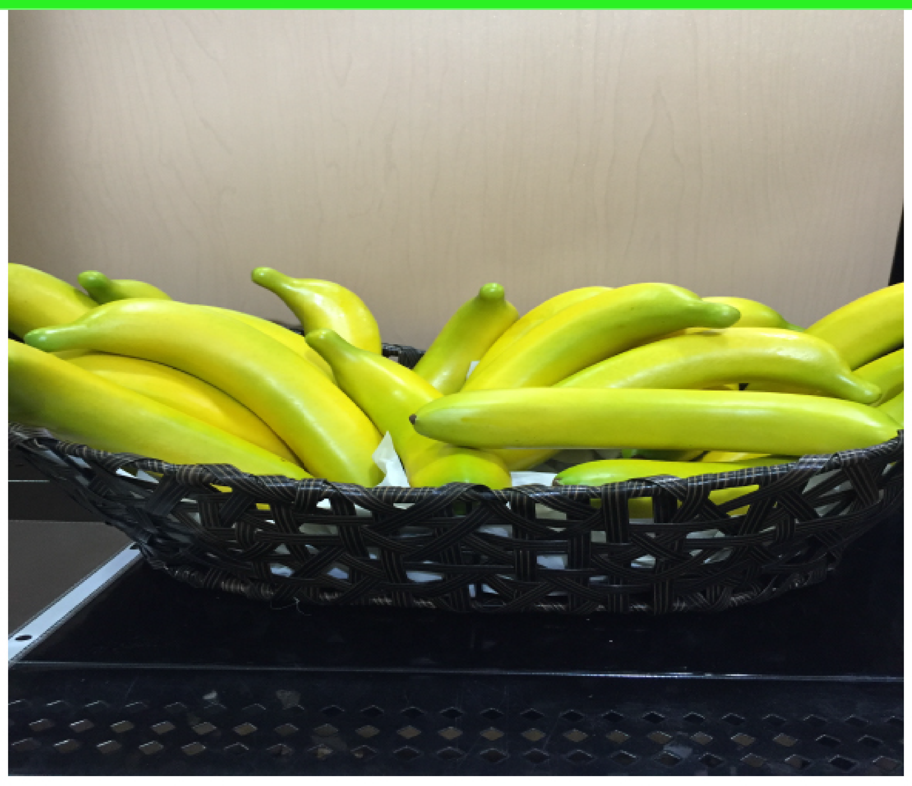

PAY ONLY 1% TAX

ORANGE JUICE 100% PURE
$2.99

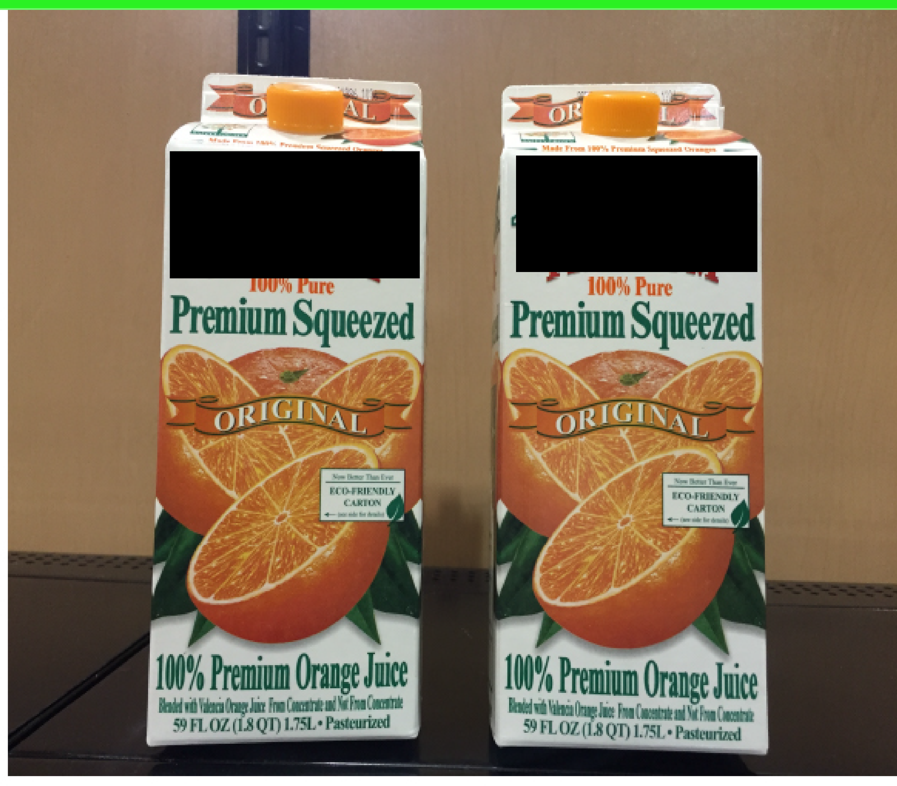

Appendix 1

PAY ONLY 1% TAX ON SELECTED ITEMS!

*APPLY TO ALL THE ITEMS WITH GREEN PRICE LABELS!

PAY ONLY 1% TAX

MULTIGRA IN BREAD $2.29

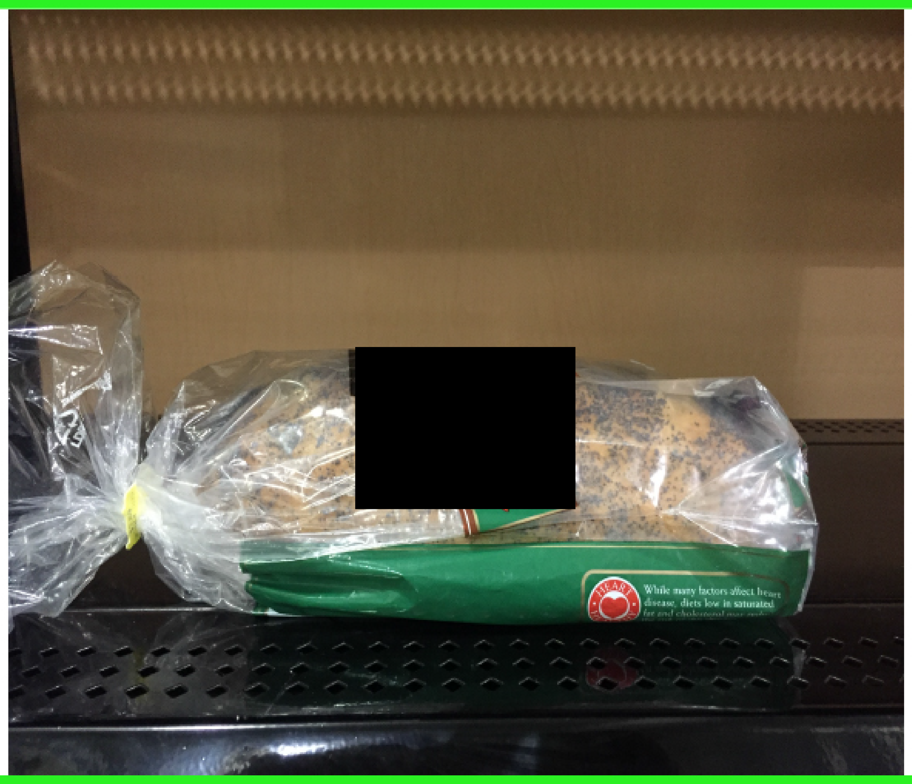

PAY ONLY 1% TAX

BROWN RICE $1.99

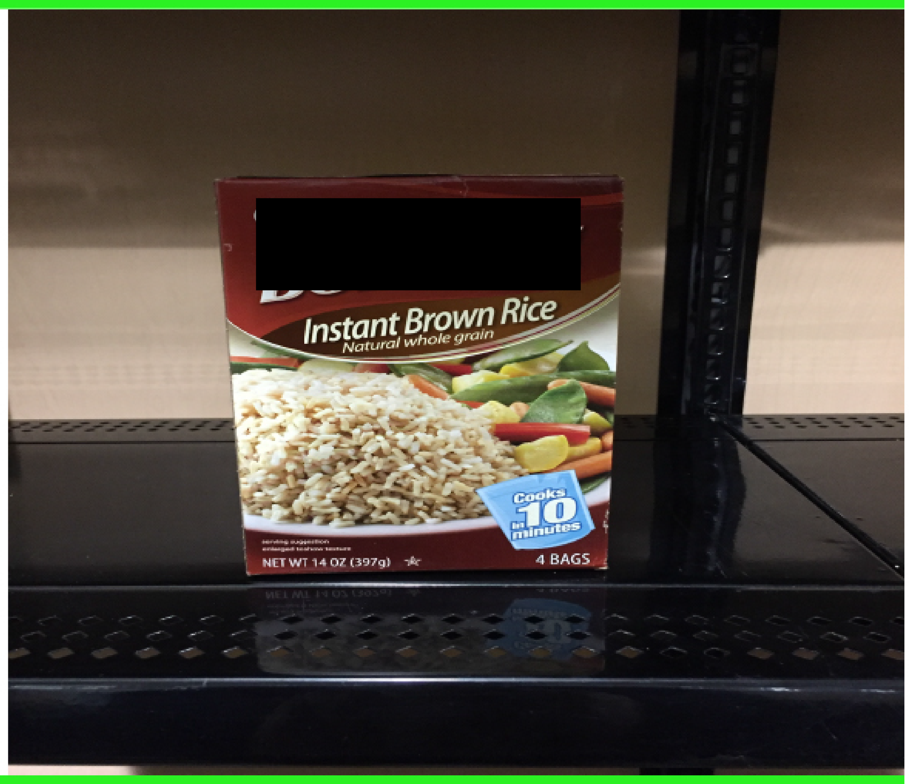

PAY ONLY 1% TAX

WHOLE GRAIN OATMEAL $3.25

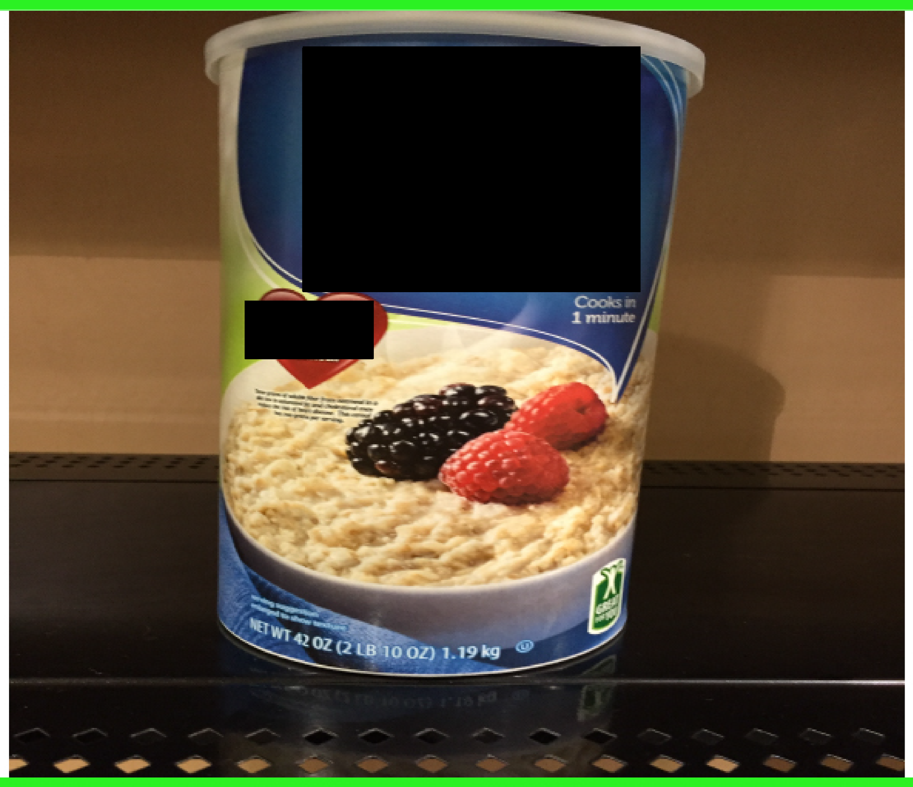

PAY ONLY 1% TAX

WHOLE GRAIN CHEERIOS $4.70

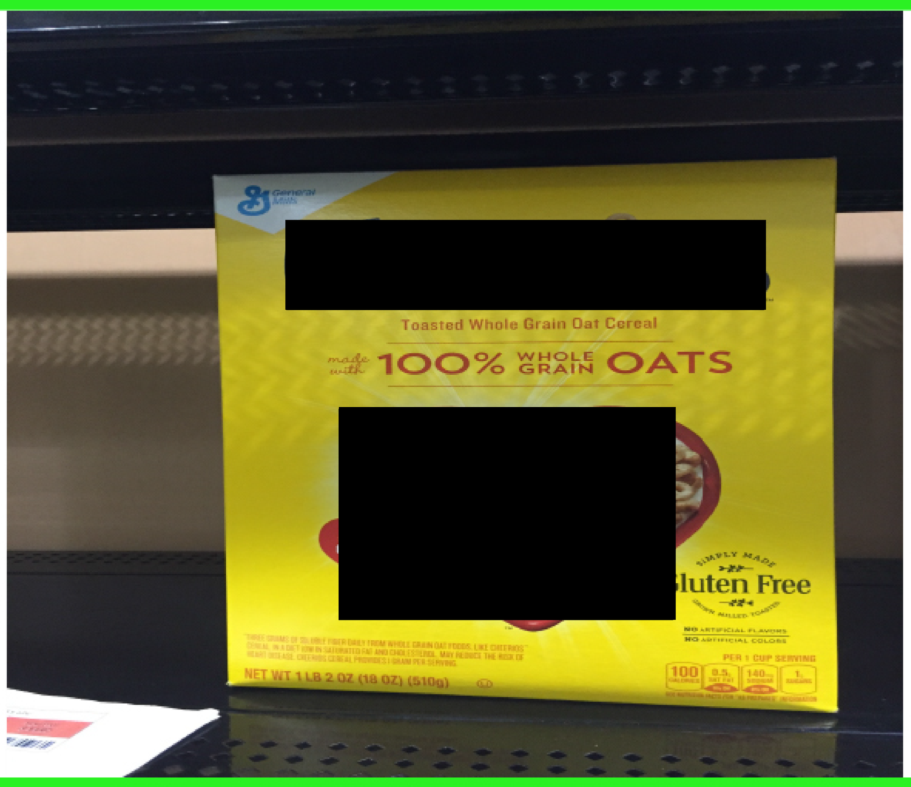

PAY ONLY 1% TAX

WHEAT CRAKERS $2.60

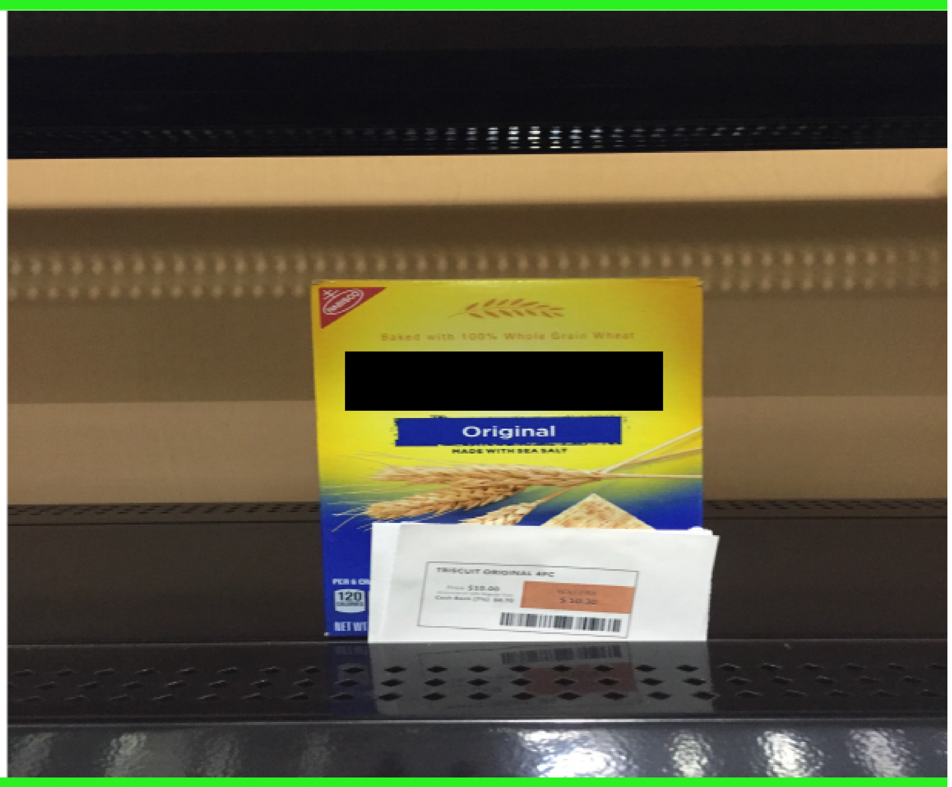

PAY ONLY 1% TAX

BUTTERLE SS POPCORN $5.60

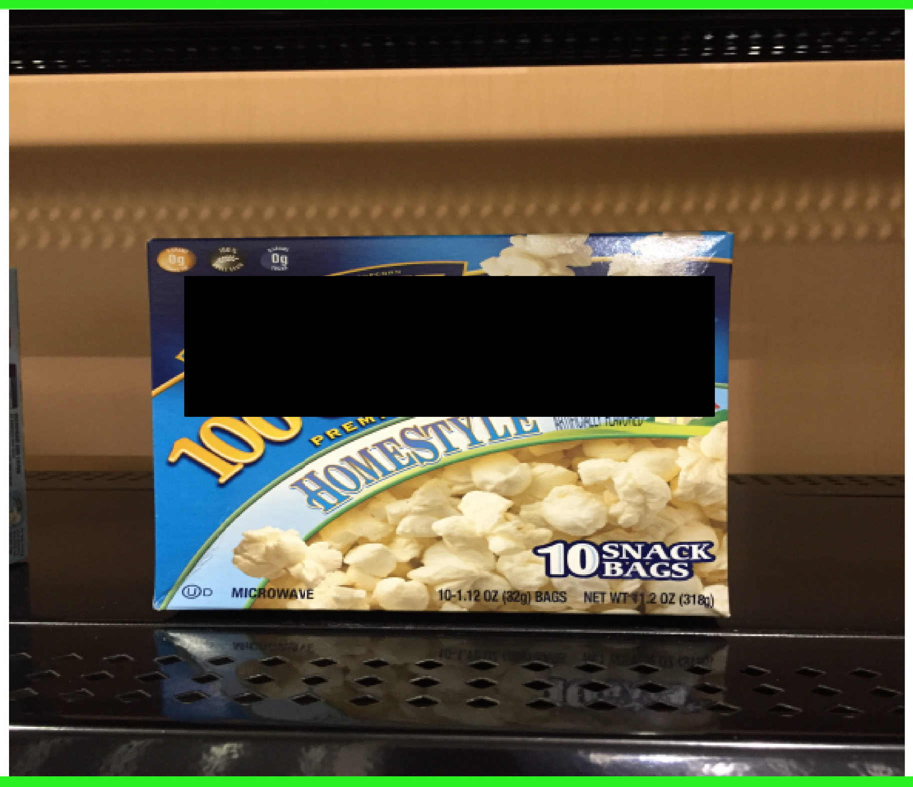

PAY ONLY 1% TAX

LOW FAT BAKED CHIPS $2.98

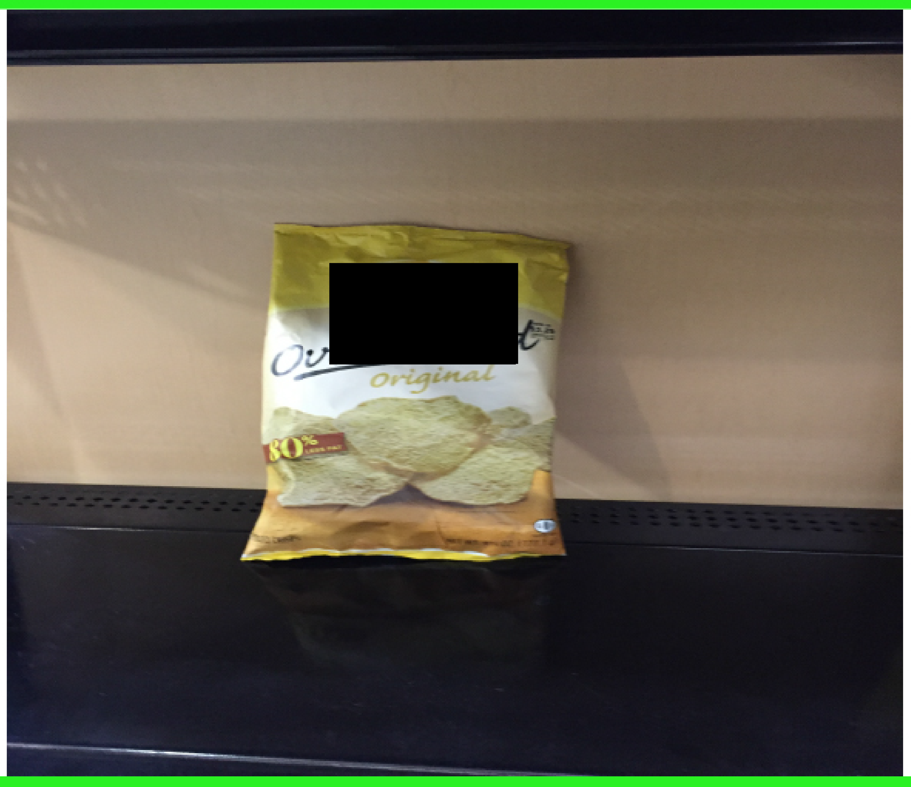

PAY ONLY 1% TAX

MILK 2% FAT $3.69

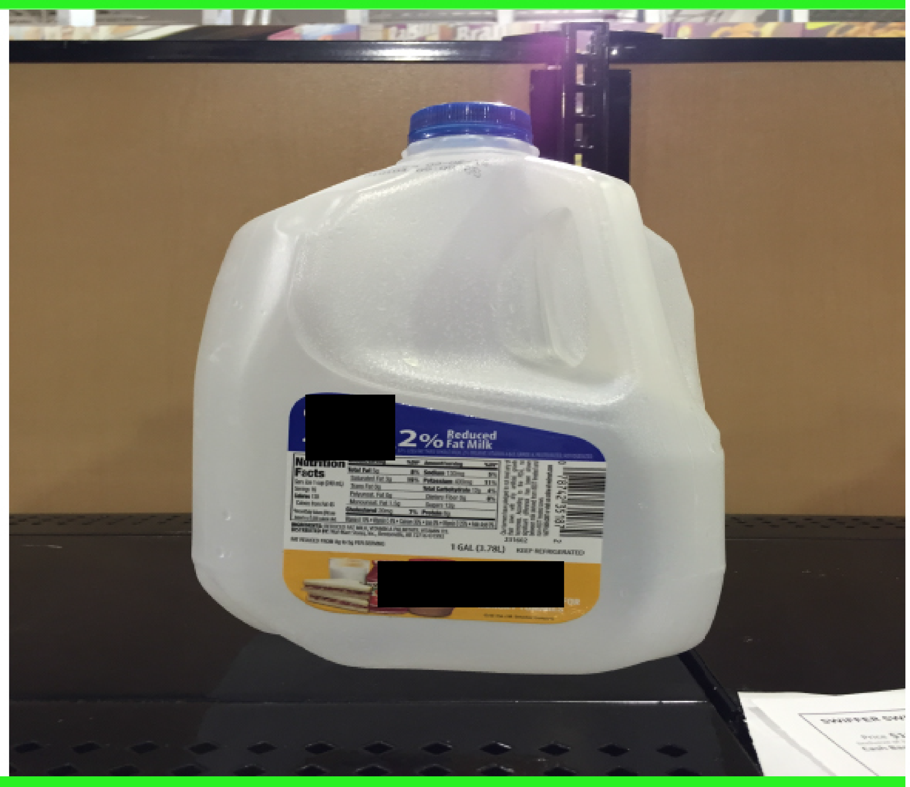

PAY ONLY 1% TAX

0 FAT YOGURT $2.99

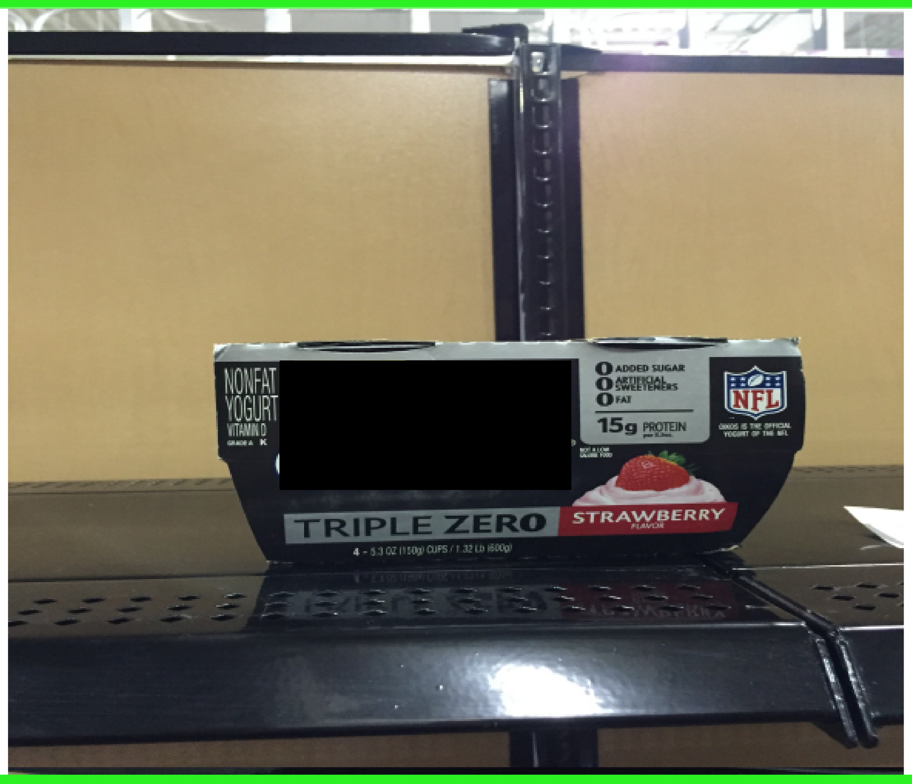

PAY ONLY 1% TAX

EGGS $3.29

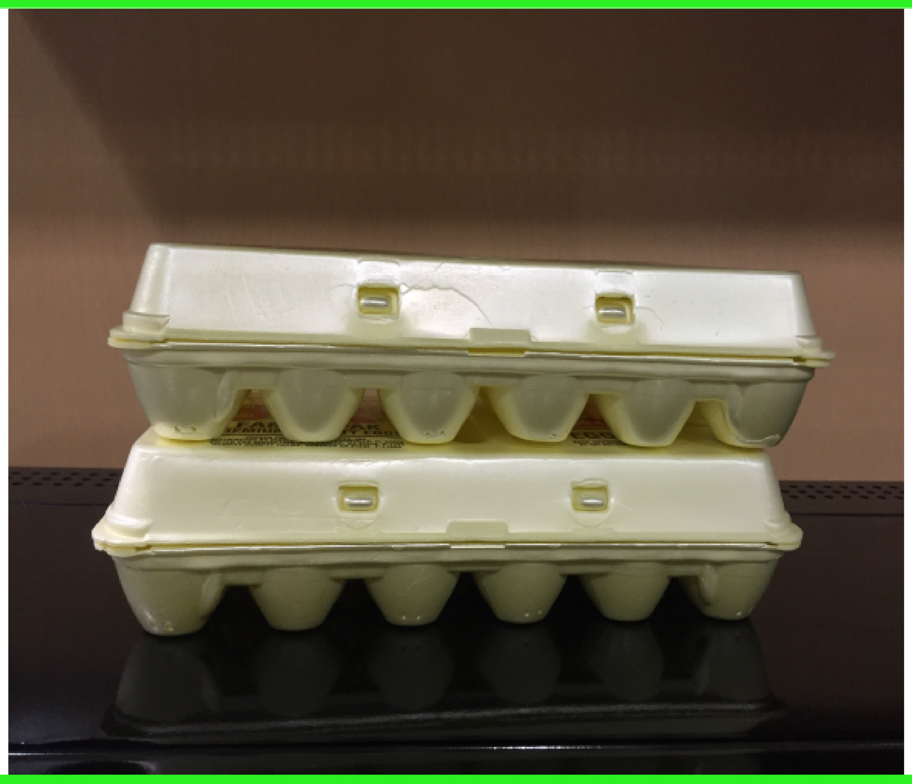

PAY ONLY 1% TAX

DARK CHOCOLATE $3.50

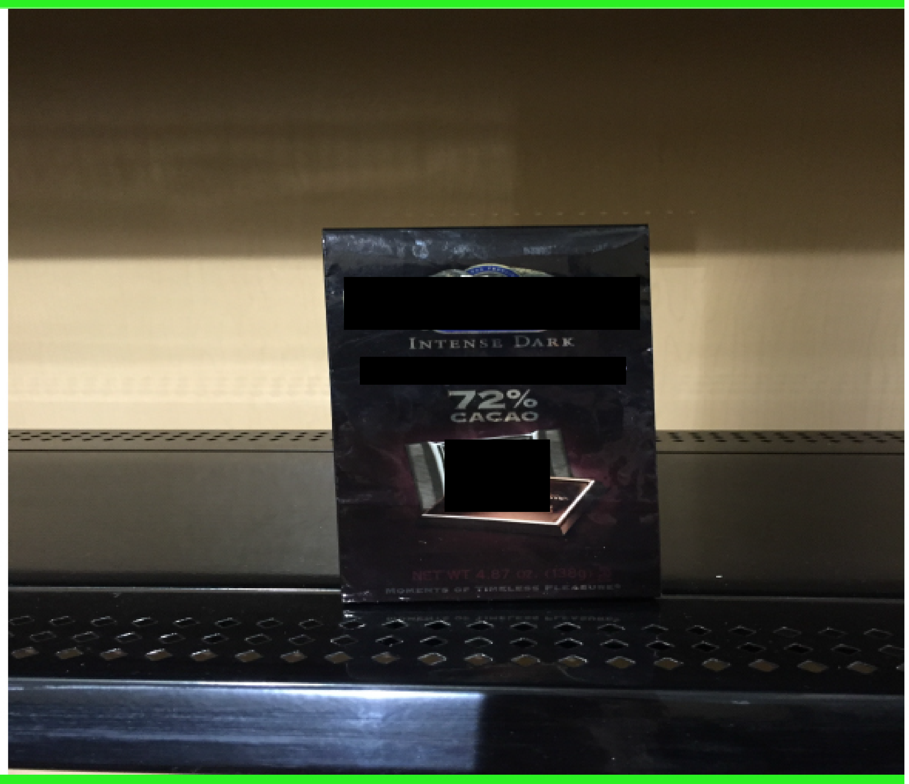

PAY ONLY 1% TAX

LEAN BEEF $3.88 /LB

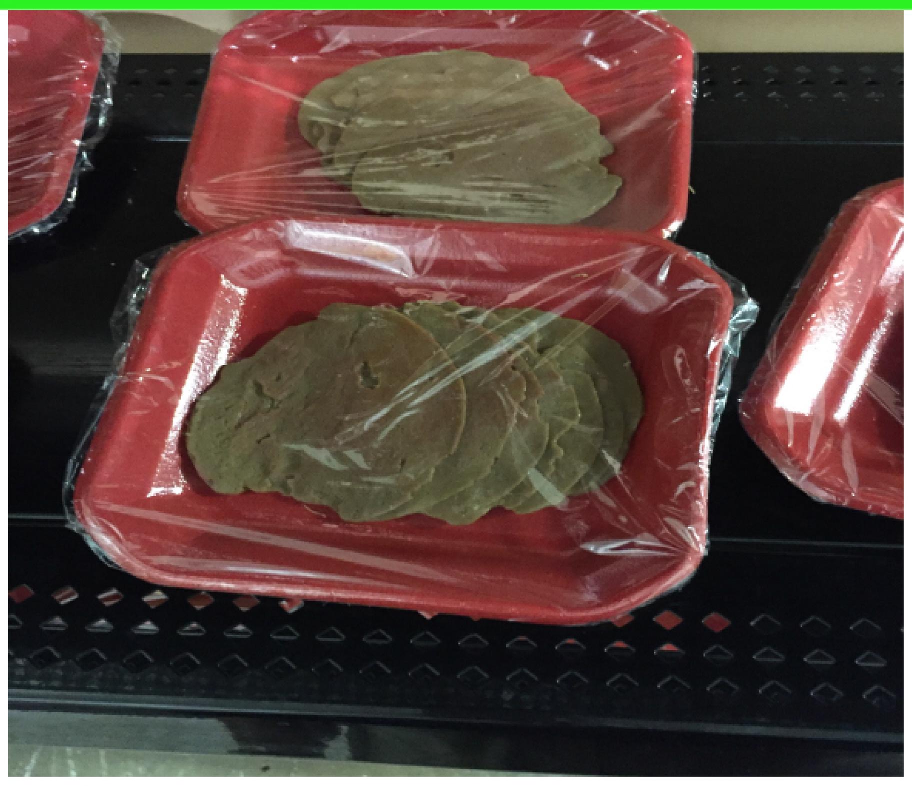

PAY ONLY 1% TAX

CHICKEN BREAST $3.24 /LB

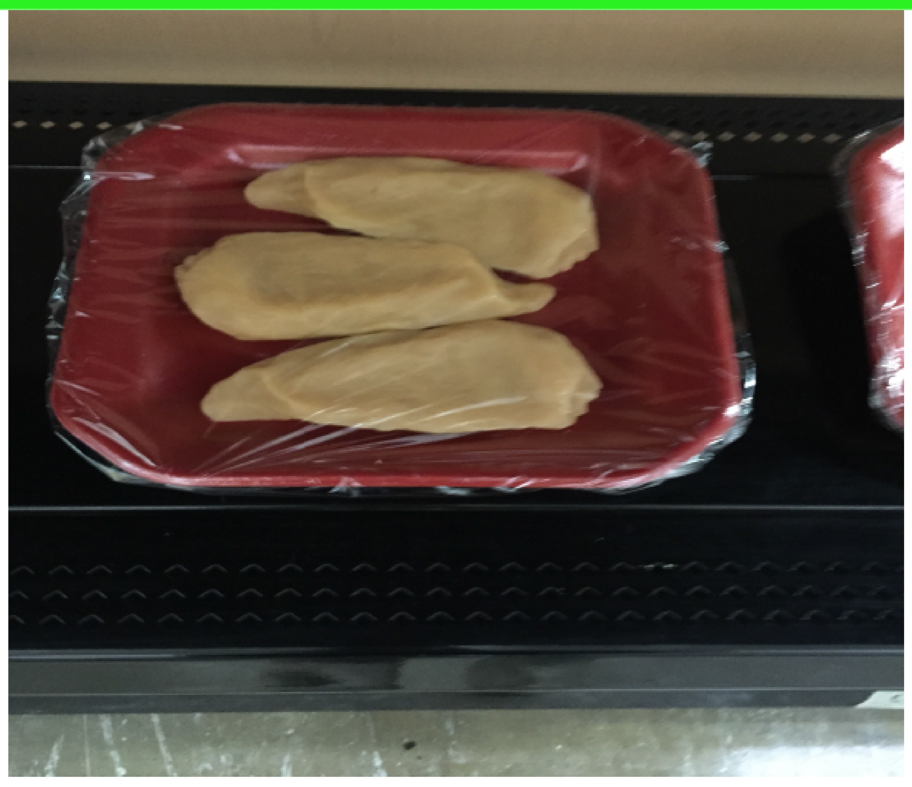

PAY ONLY 1% TAX

LOW FAT PEANUT BUTTER $2.73

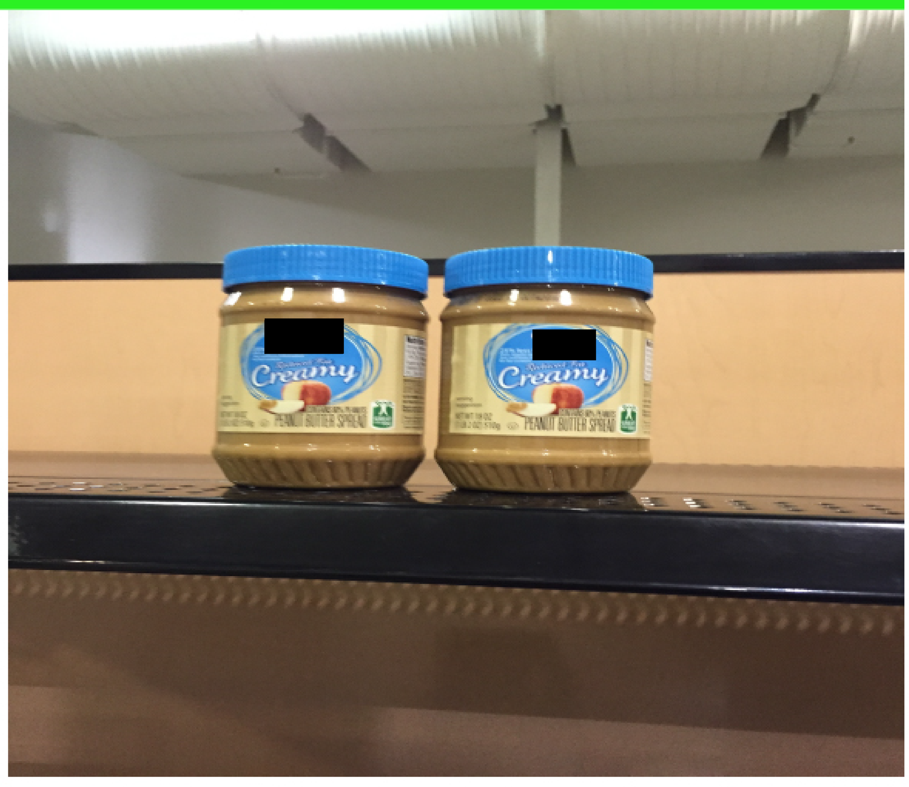

Appendix 1

EARN 9% CASHBACK ON SELECTED ITEMS!

*APPLY TO ALL THE ITEMS WITH BLUE PRICE LABELS!

EARN 9% CASHBACK

CARROTS
$0.88 /LB

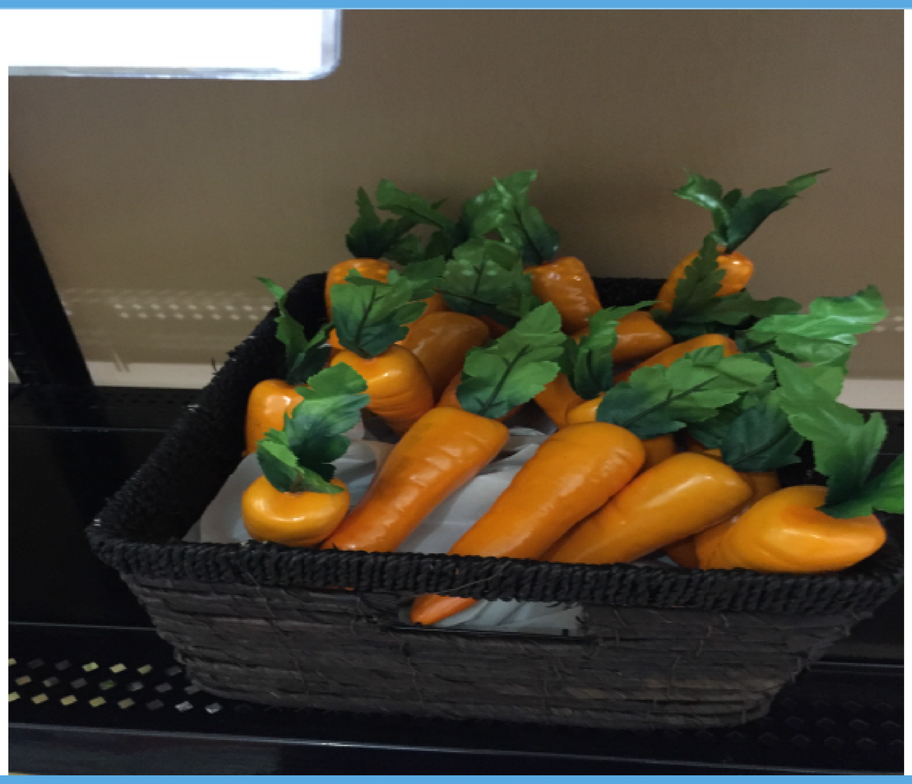

EARN 9% CASHBACK

BROCCOLI
$2.37 EA

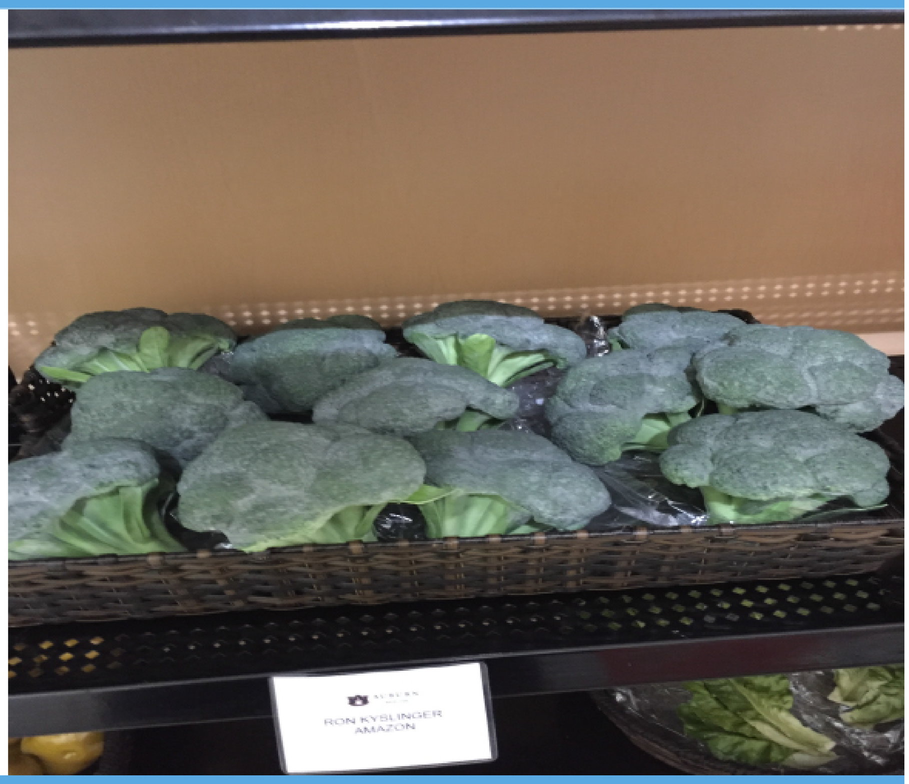

EARN 9% CASHBACK

ASPATAGUS
$4.63 /lb

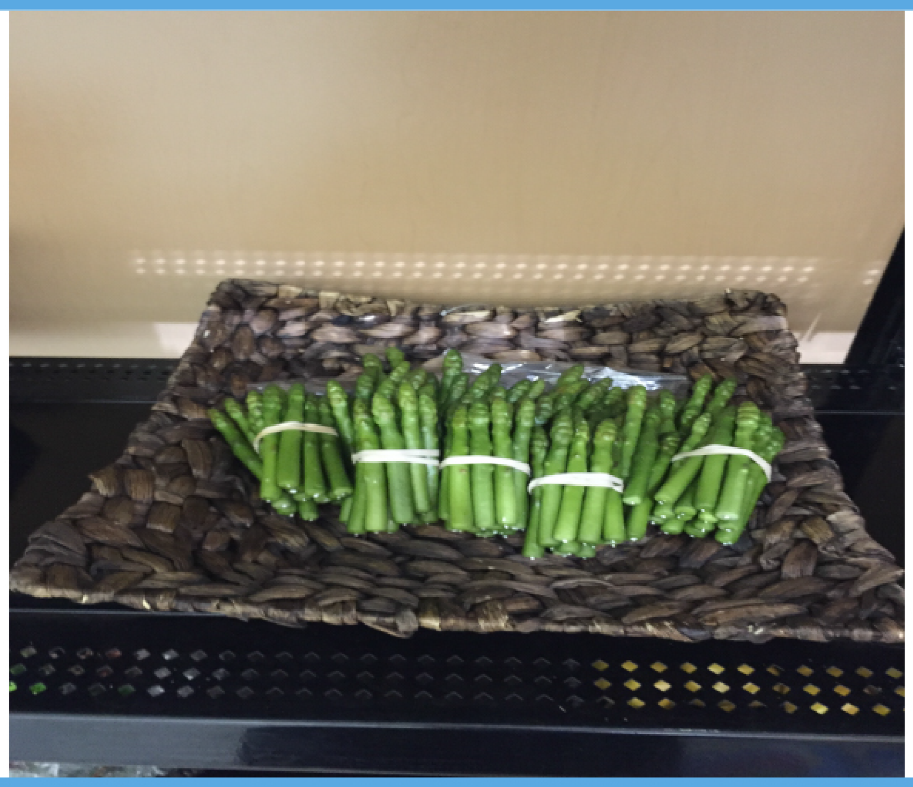

EARN 9% CASHBACK

BELL PEPPER
$1.65 /lb

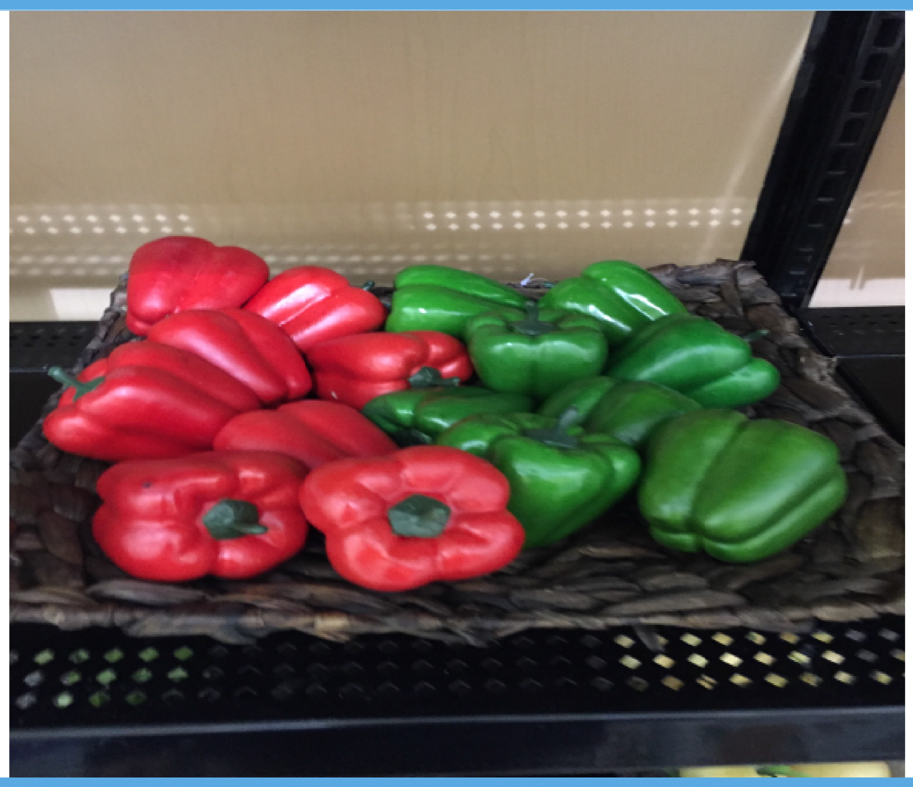

EARN 9% CASHBACK

GREEN BEANS
$1.45 /lb

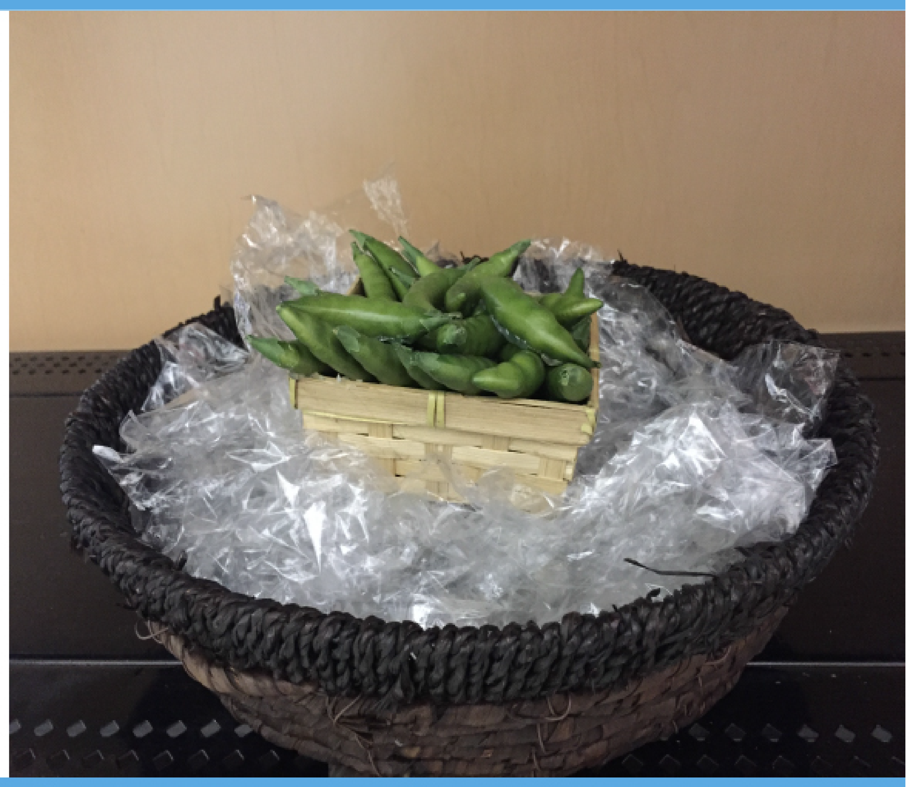

EARN 9% CASHBACK

LETTUCE
$1.25 /LB

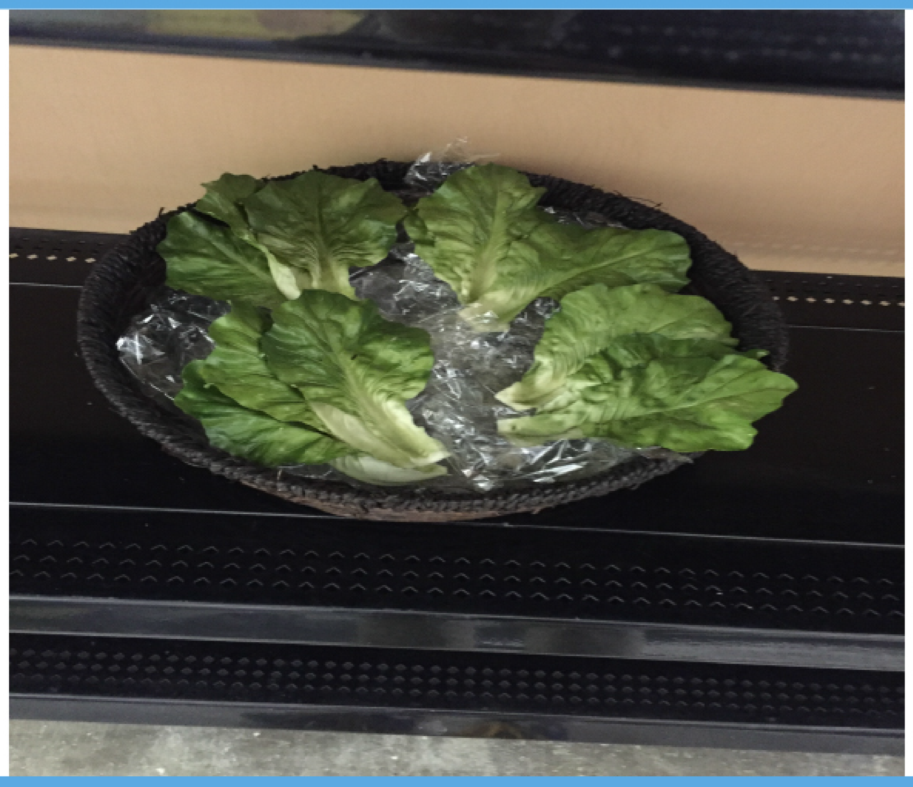

EARN 9% CASHBACK

WHITE POTATO
$1.10 /LB

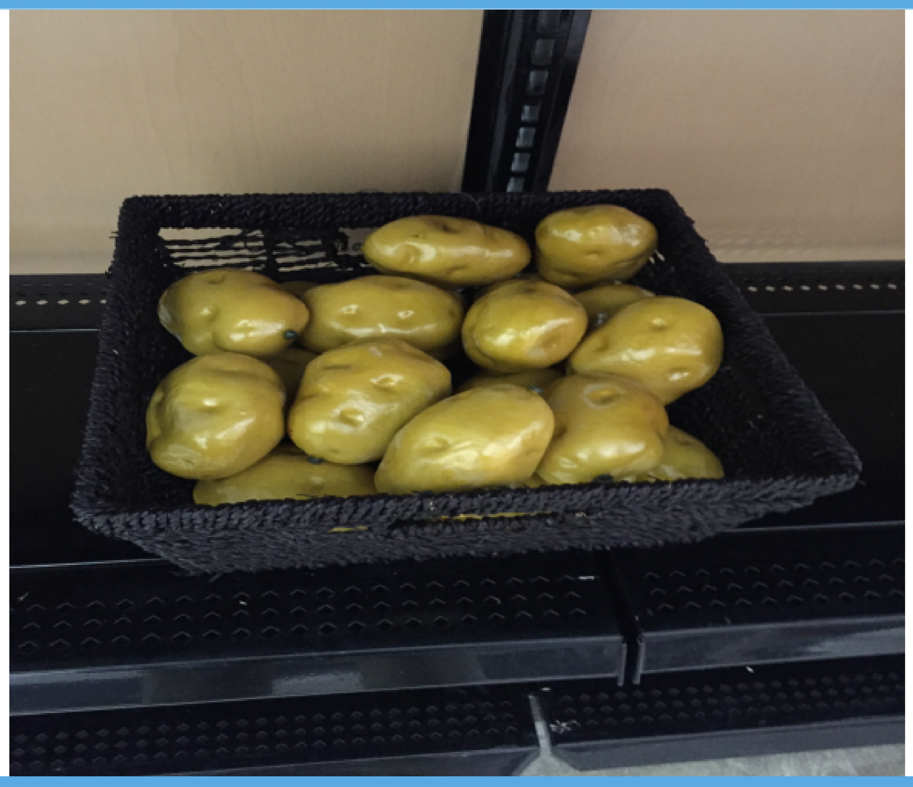

EARN 9% CASHBACK

PEACH
$2.49 /LB

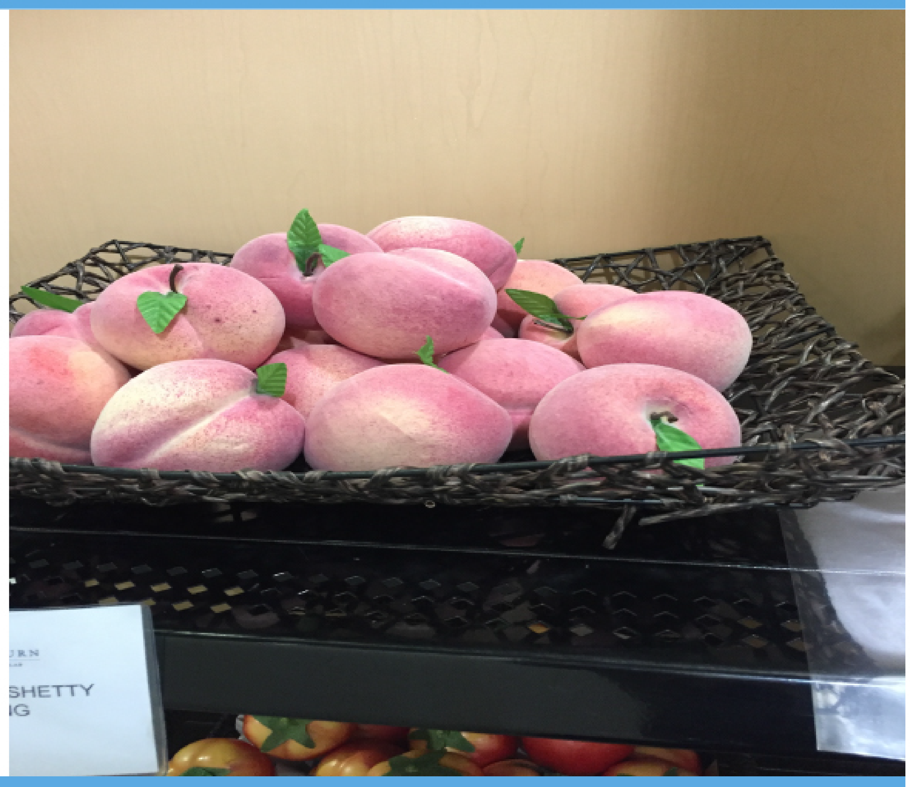

EARN 9% CASHBACK

ORANGE
$0.80 EA

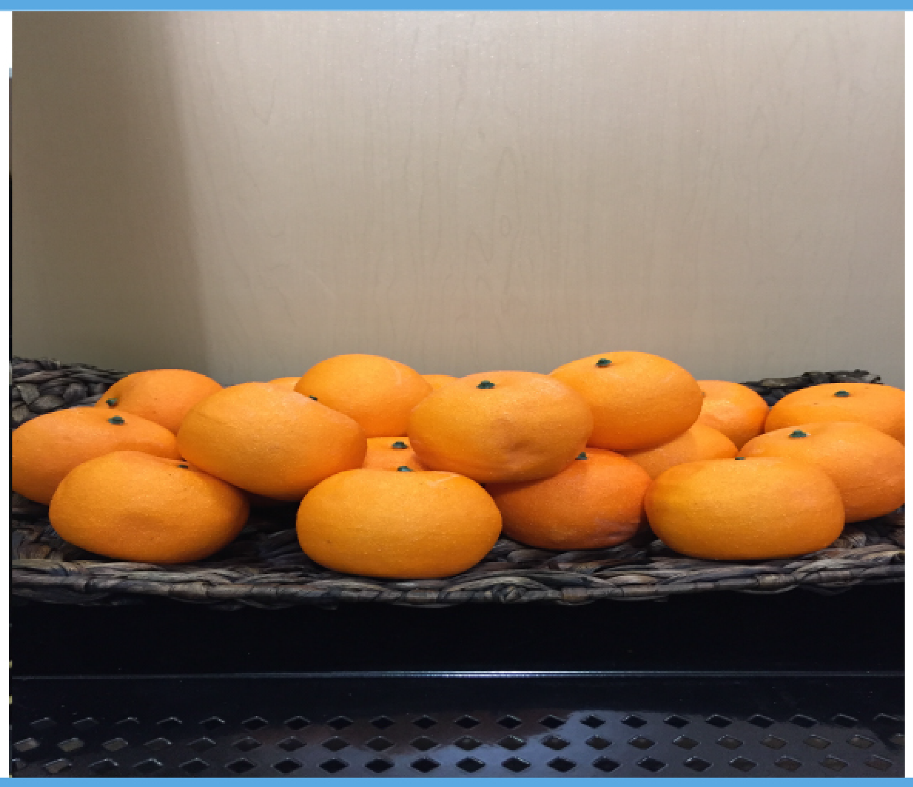

EARN 9% CASHBACK

PEAR
$2.40 /LB

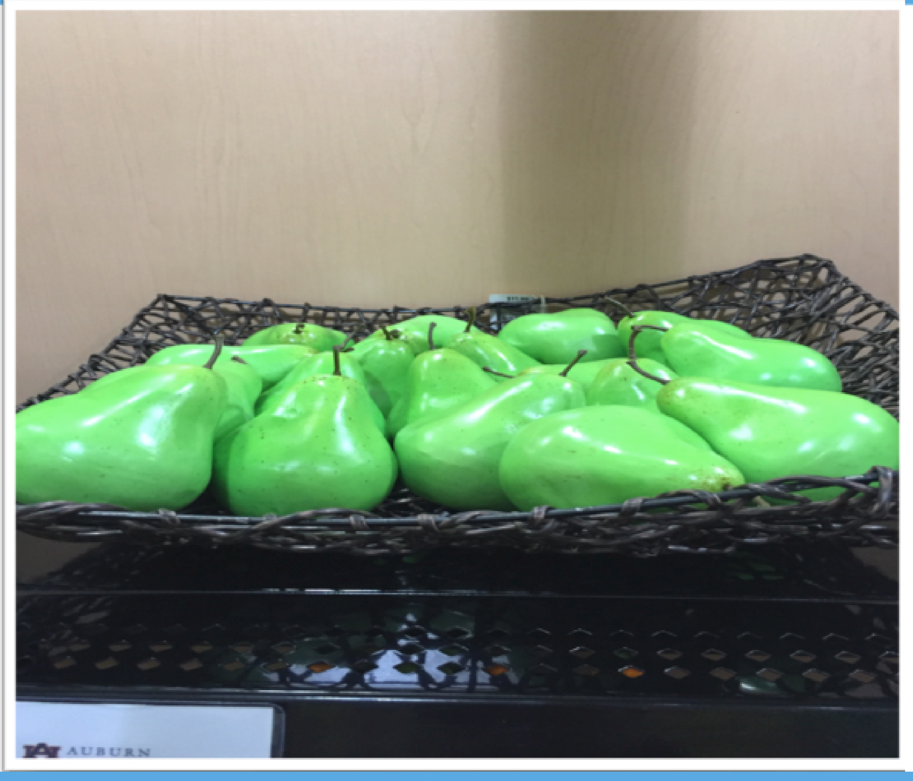

EARN 9% CASHBACK

PINEAPPLE
$2.80 /EA

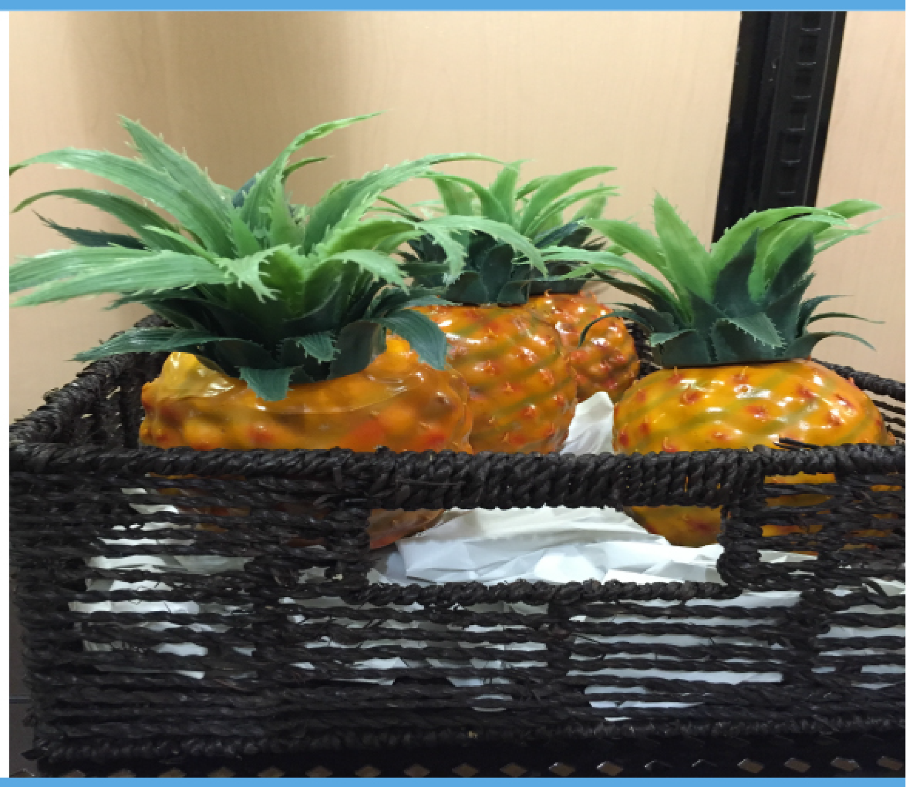

EARN 9% CASHBACK

APPLE
$2.00 /LB

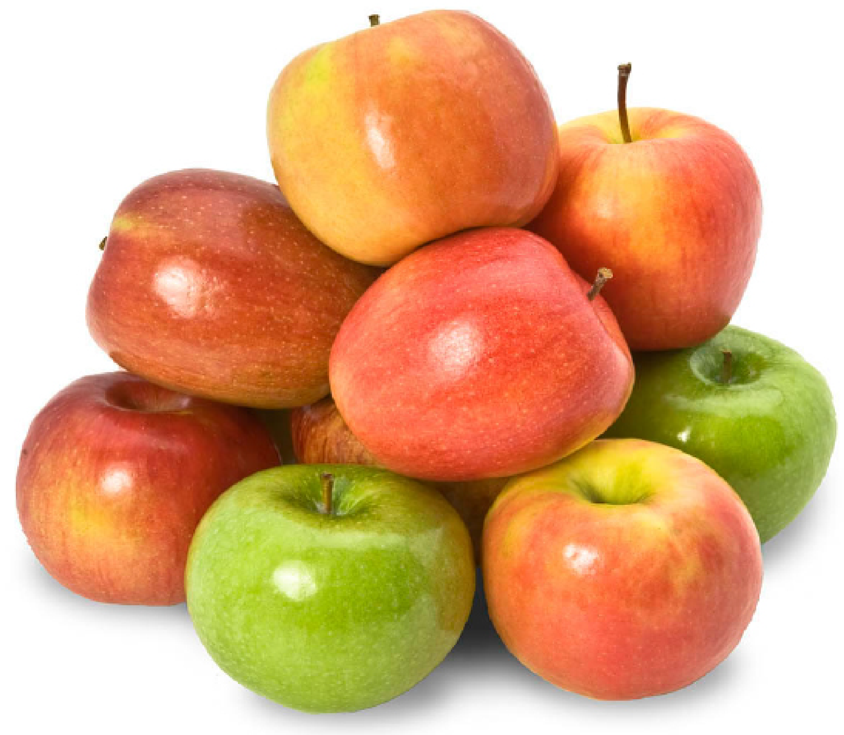

EARN 9% CASHBACK

BANANA
$0.59 /LB

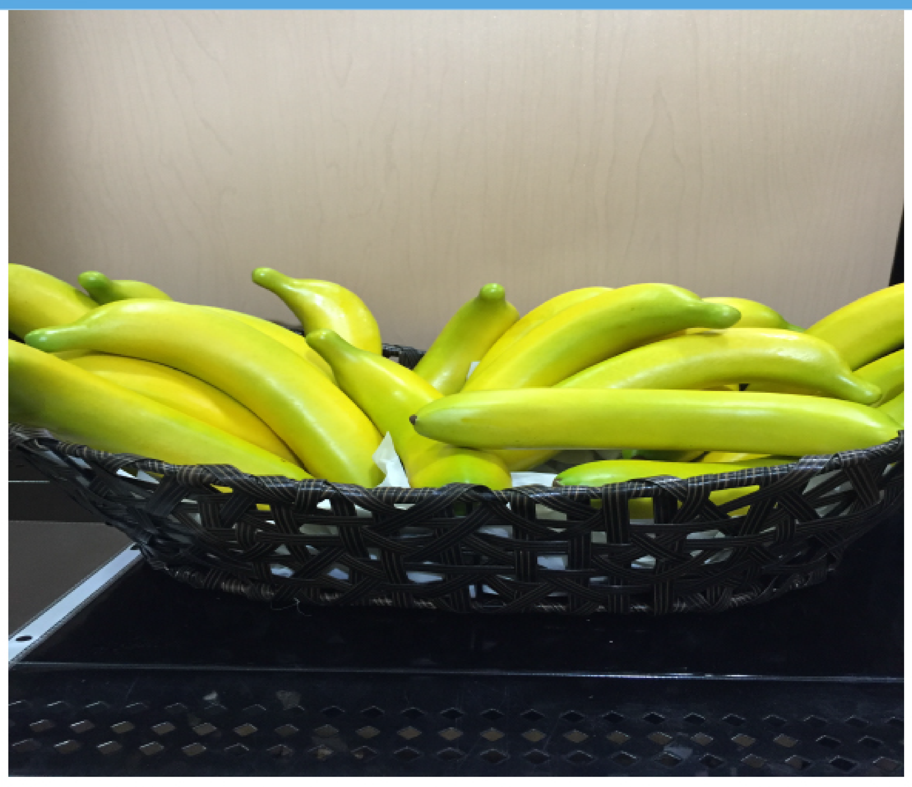

EARN 9% CASHBACK

ORANGE JUICE 100% PURE
$2.99

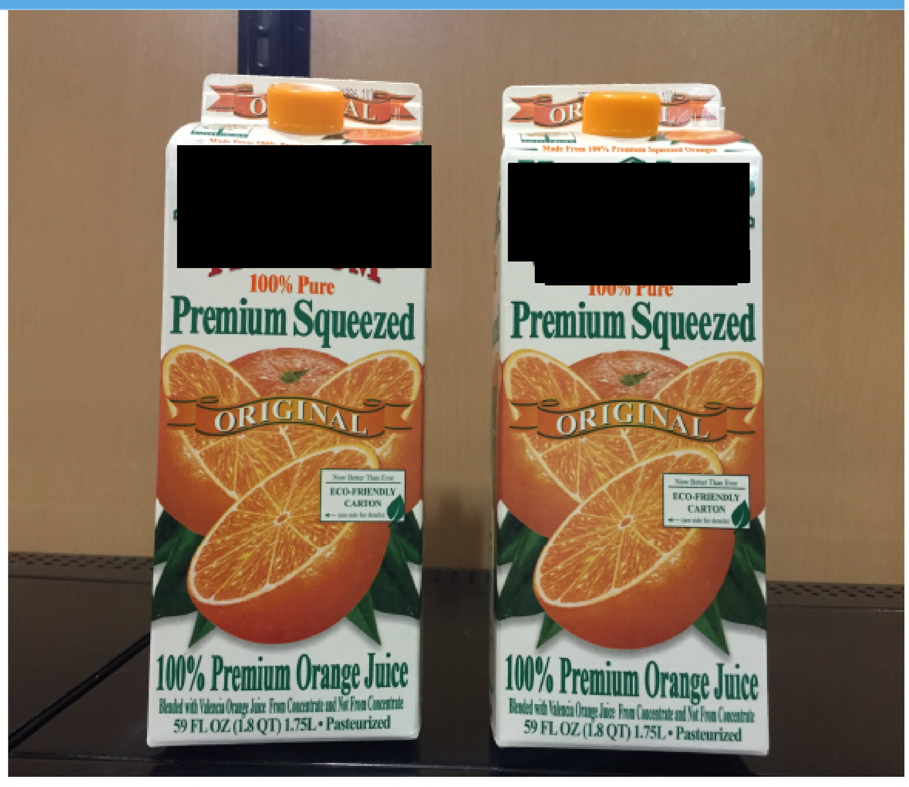

Appendix 1

EARN 9% CASHBACK ON SELECTED ITEMS!

*APPLY TO ALL THE ITEMS WITH BLUE PRICE LABELS!

EARN 9% CASHBACK

MULTIGRAIN BREAD
$2.29

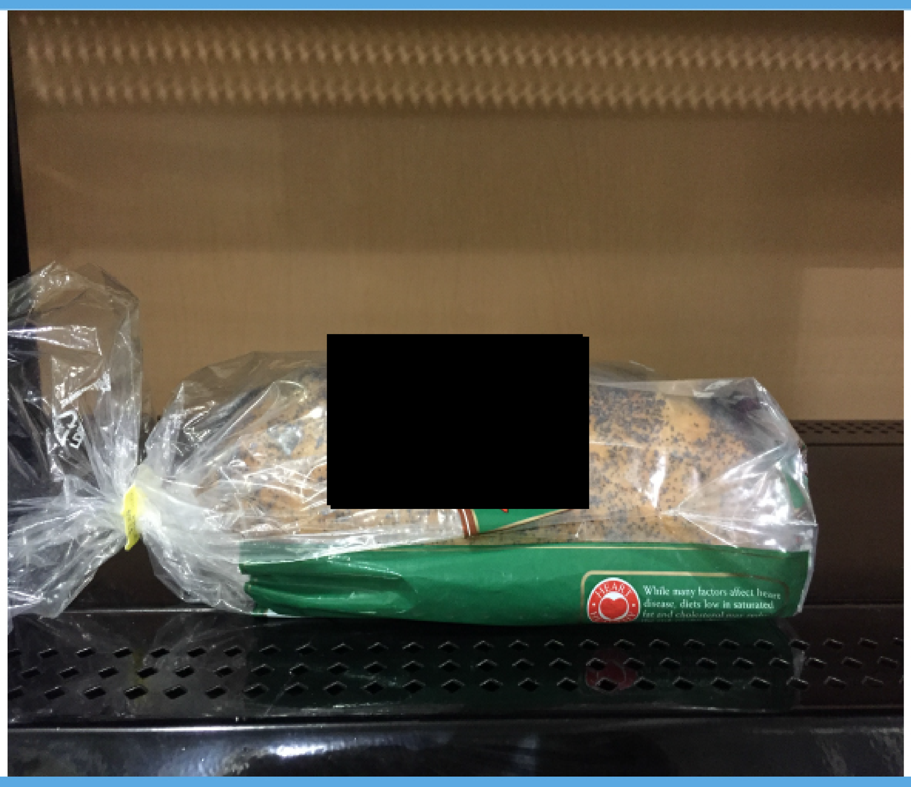

EARN 9% CASHBACK

BROWN RICE
$1.99

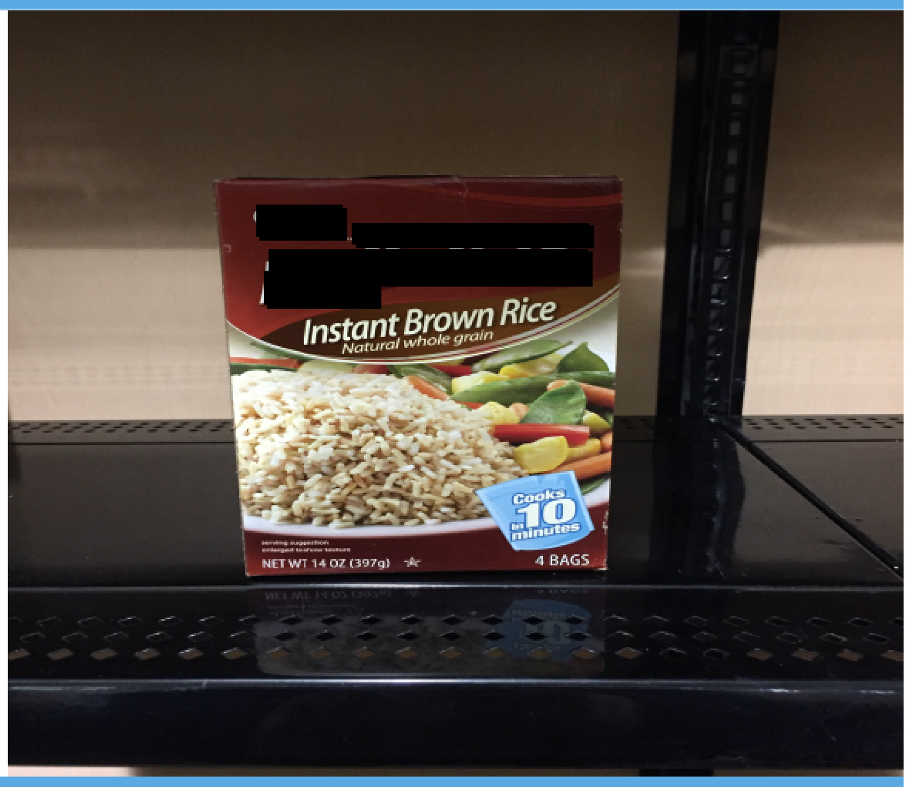

EARN 9% CASHBACK

WHOLE GRAIN OATMEAL
$3.25

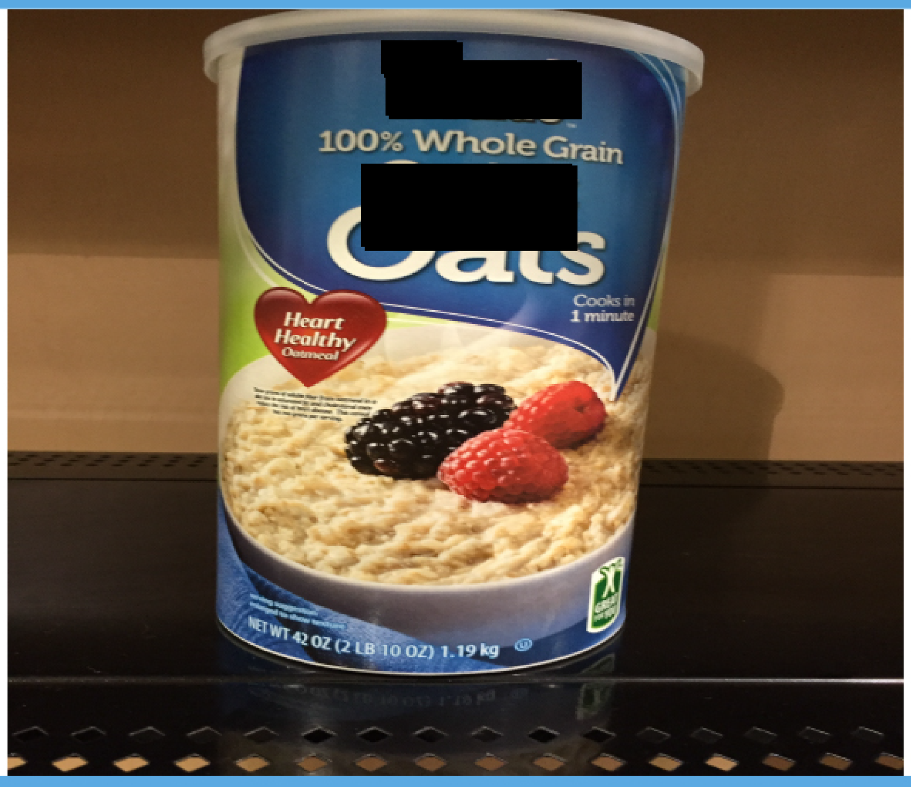

EARN 9% CASHBACK

WHOLE GRAIN CHEERIOS
$4.70

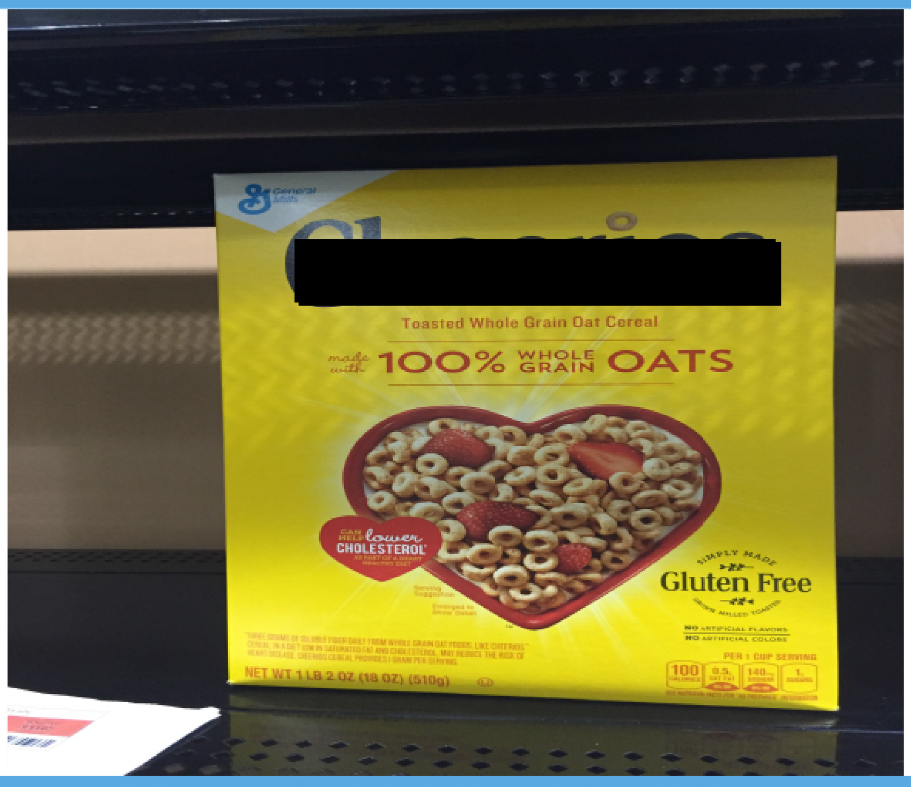

EARN 9% CASHBACK

WHEAT CRACKERS
$2.60

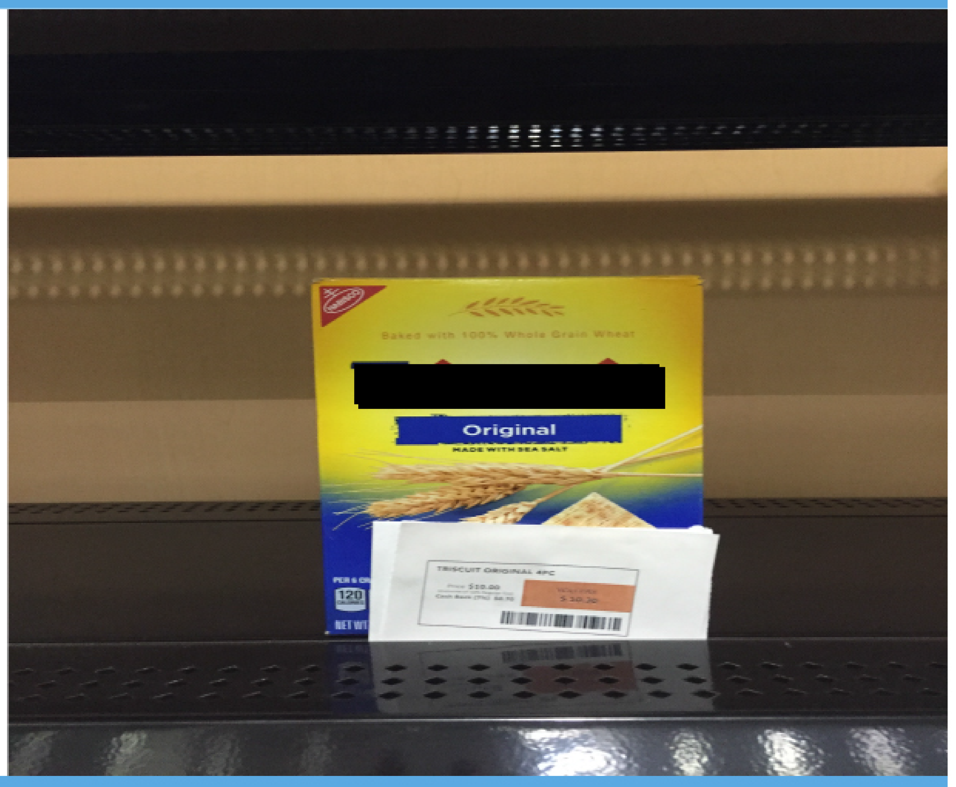

EARN 9% CASHBACK

BUTTERLESS POPCORN
$5.60

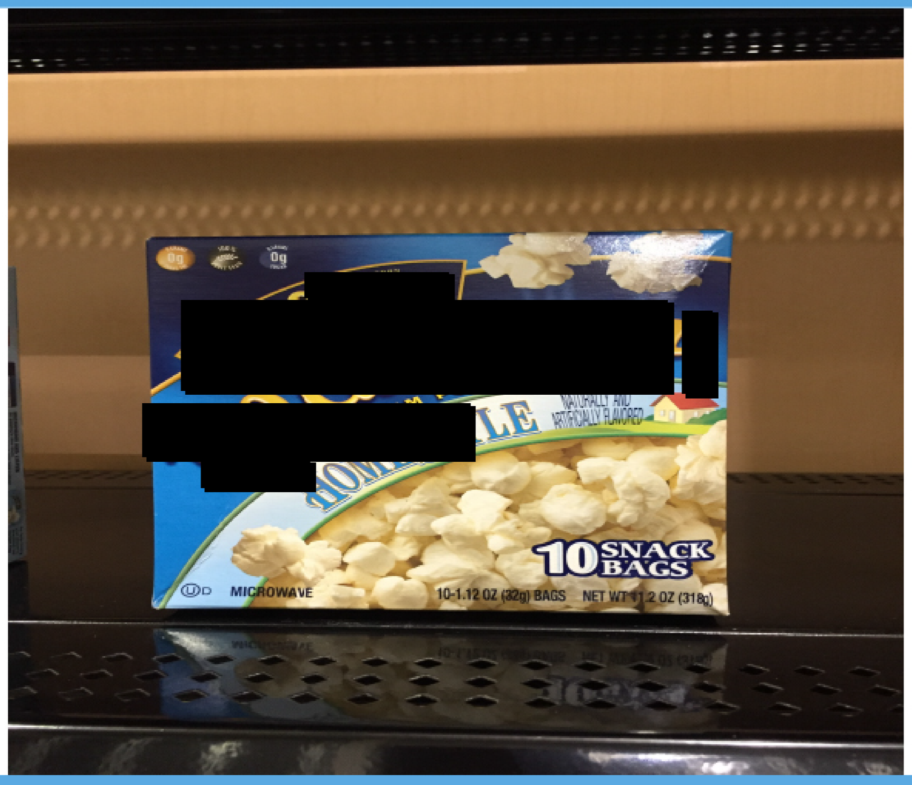

EARN 9% CASHBACK

LOW FAT BAKED CHIPS
$2.98

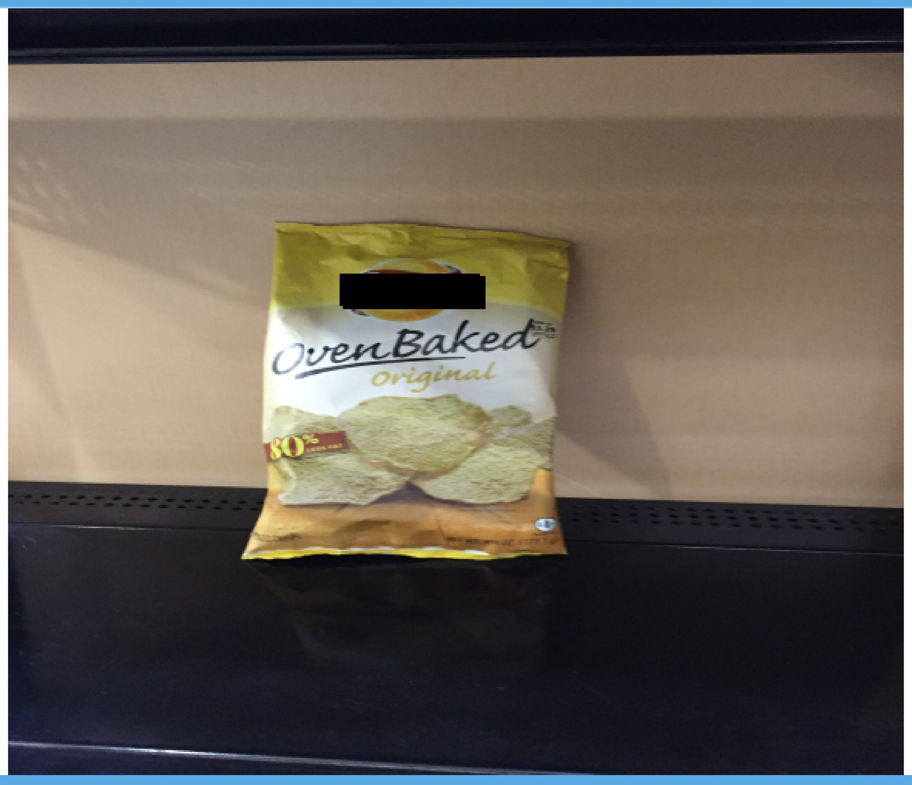

EARN 9% CASHBACK

MILK 2% FAT
$3.69

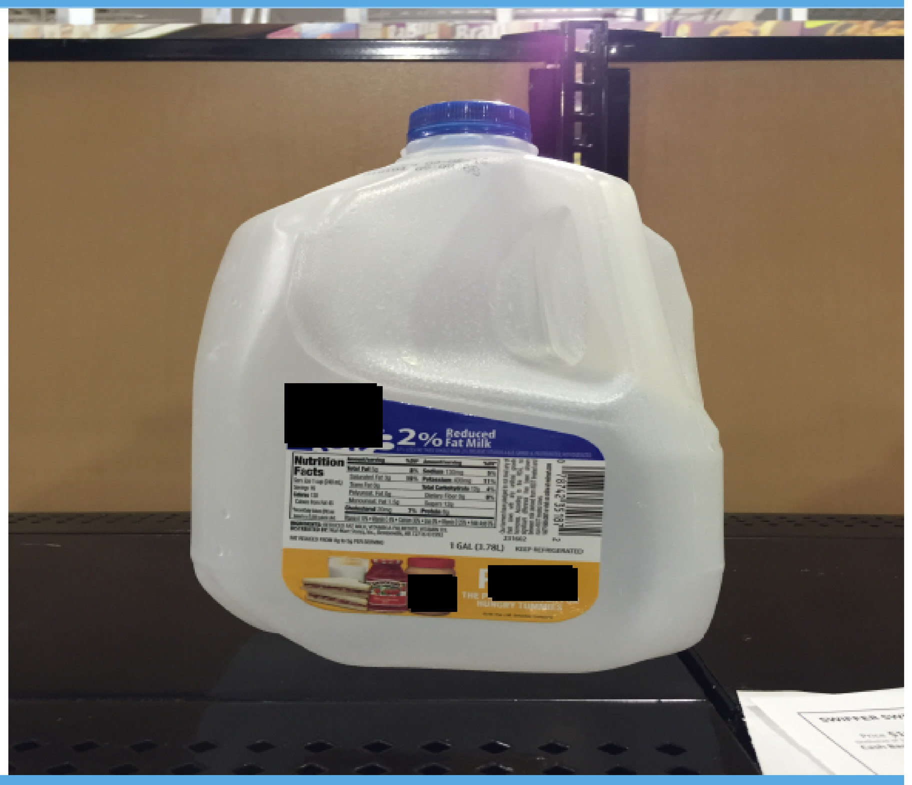

EARN 9% CASHBACK

0 FAT YOGURT
$2.99

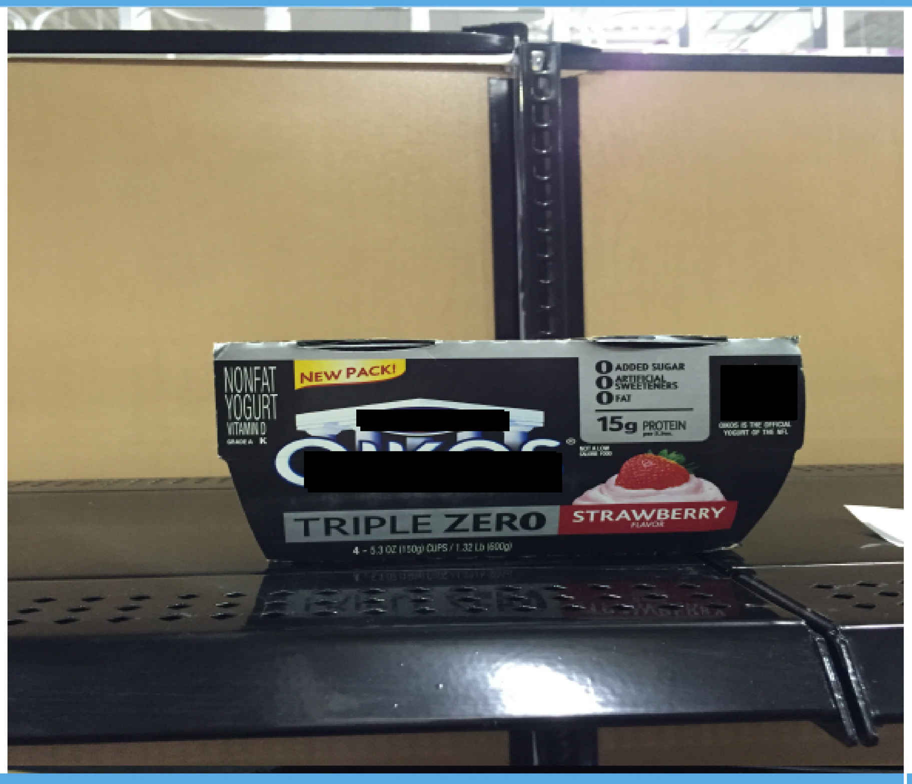

EARN 9% CASHBACK

EGGS
$3.29

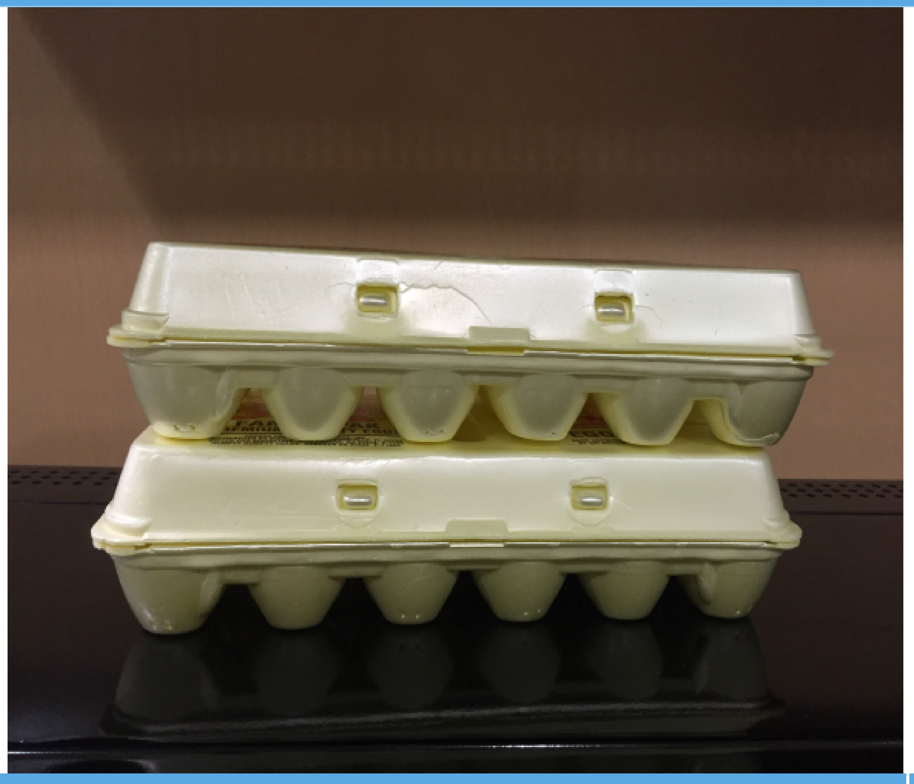

EARN 9% CASHBACK

DARK CHOCOLATE
$3.50

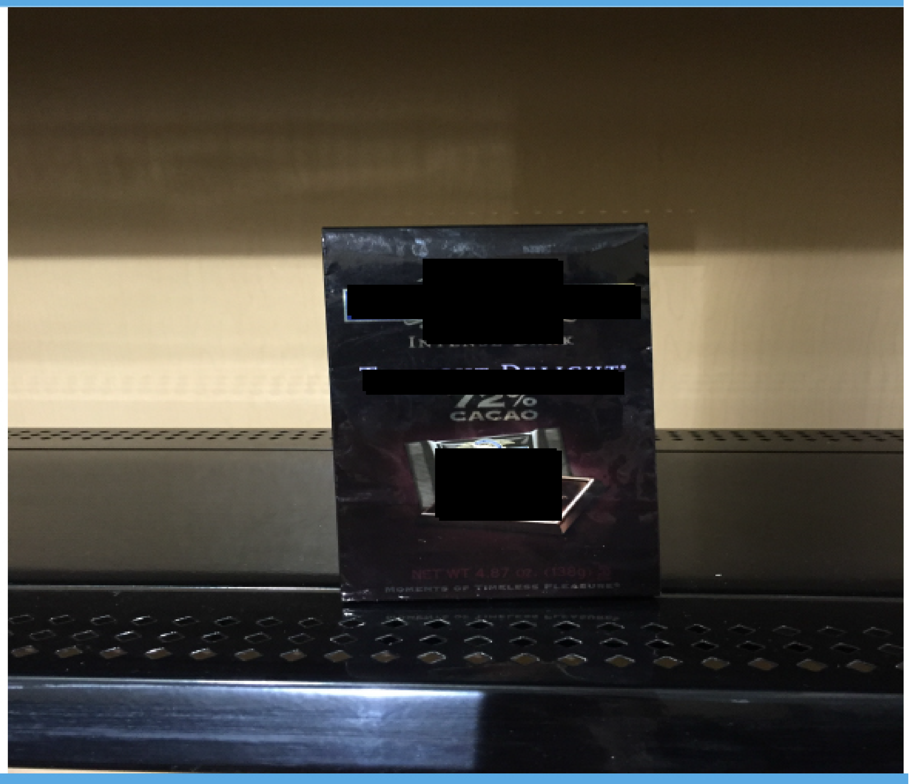

EARN 9% CASHBACK

LEAN BEEF
$3.88 /LB

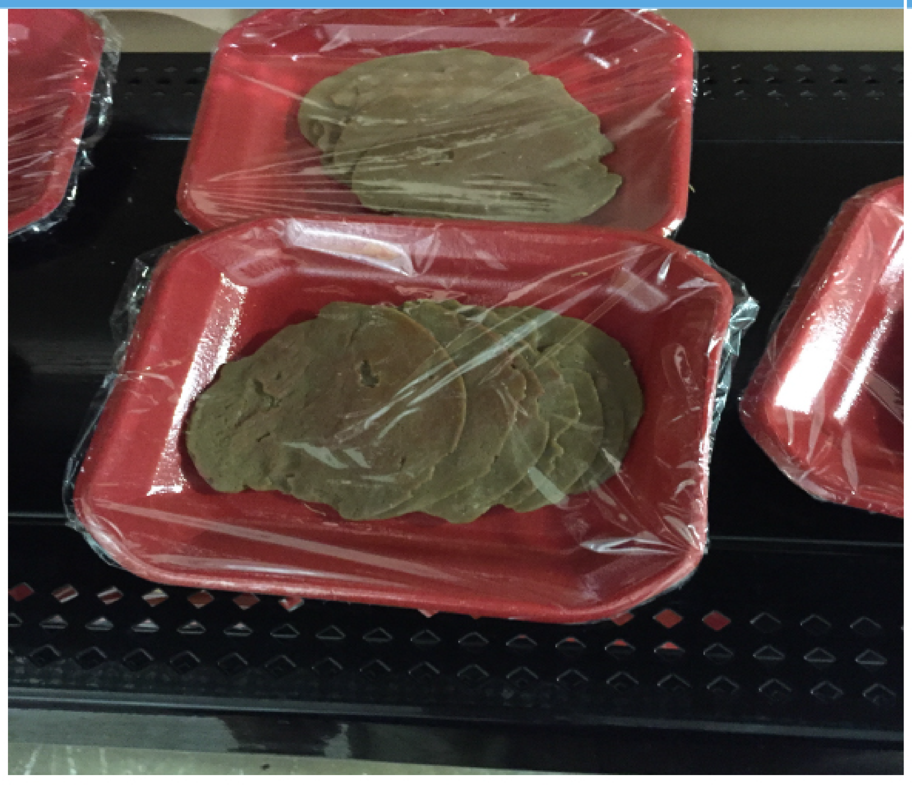

EARN 9% CASHBACK

CHICKEN BREAST
$3.24 /LB

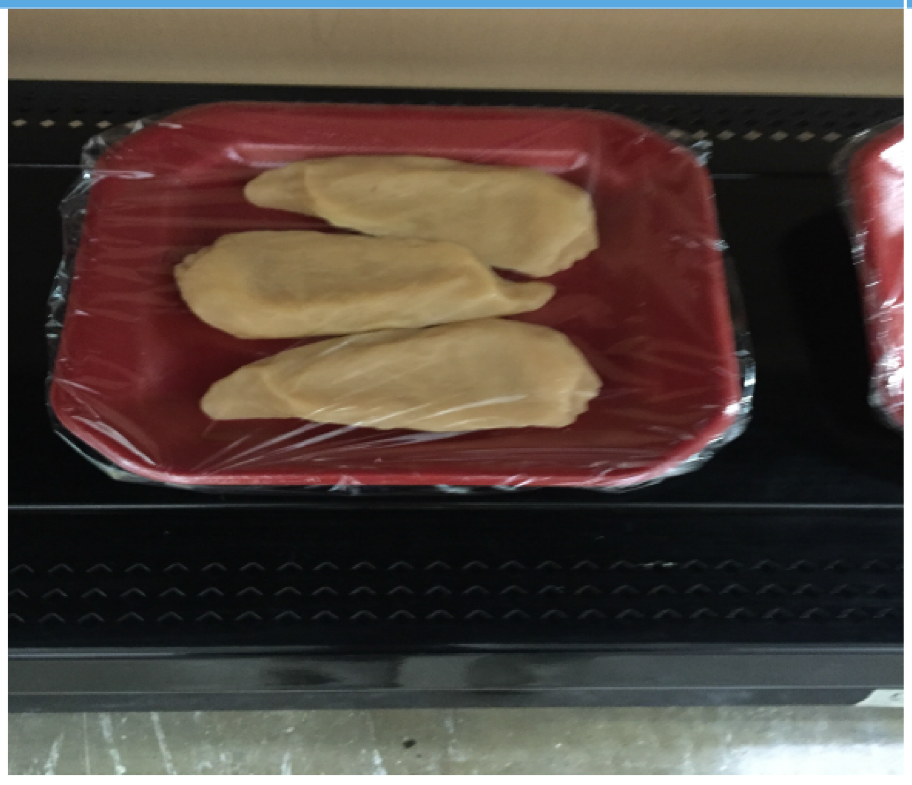

EARN 9% CASHBACK

LOW FAT PEANUT BUTTER
$2.73

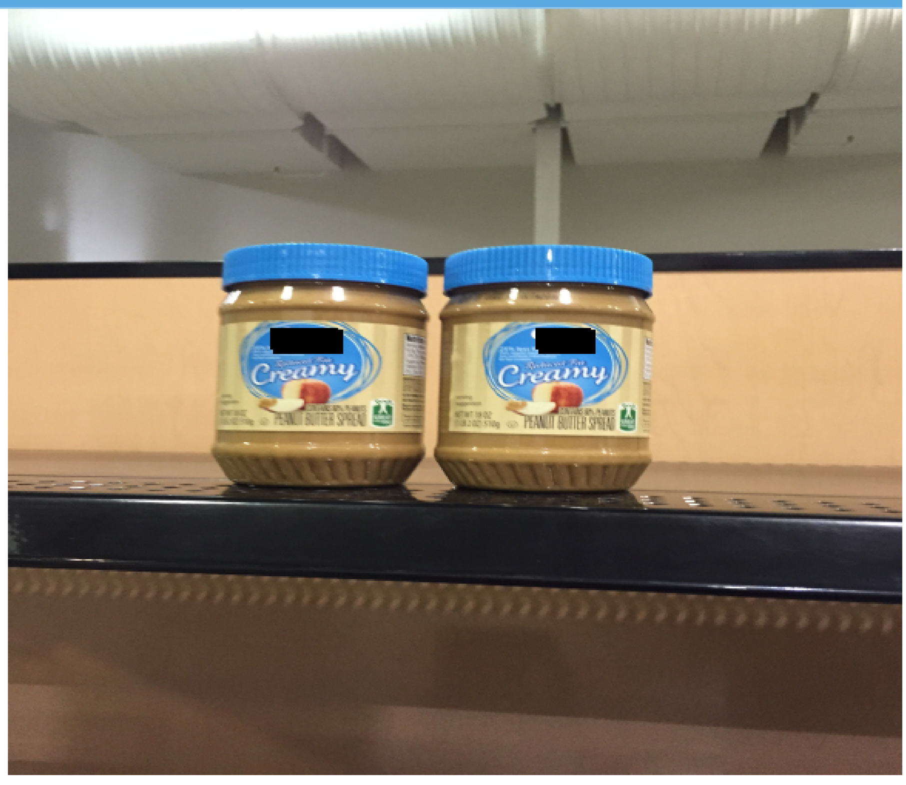

Appendix 2

Condition #:__ Participant #: _____________

PART I: DEMOGRAPHIC INFORMATION

1. Did you experience any price promotion at the grocery store you just shopped?
   ______Yes
   ______No (please proceed to question #3)

2. If you answered YES to question #1, what type of price promotion did you experience at the grocery store you just shopped?

   ____ 1% TAX ______9% CASH BACK

3. What is your **gender?**

   ______Male
   ______Female

4. What is your **age?** _________ years old

5. What is **the highest level of education** you have completed?

   ______8th grade or less
   ______Some high school
   ______High school degree
   ______Some college or technical school
   ______College degree (4 years)
   ______Some graduate school
   ______Graduate degree (master's, doctorate, etc.)

6. Which of the following **ethnic groups** do you consider yourself to be a member of?

   ______Non-hispanic white (caucasian american)
   ______Non-hispanic black (african american)
   ______Hispanic
   ______Asian/pacific islander
   ______American indian/alaskan native
   ______Other (Please specify: ________________________________)

Appendix 2

7. Which of the following ranges includes your **total annual household income** from all sources?

________	Under $5,000	________	$5,000 to $9,999
________	$10,000 to $14,999	________	$15,000 to $19,999
________	$20,000 to $24,999	________	$25,000 to $29,999
________	$30,000 to $39,999	________	$40,000 to $49,999
________	$50,000 to $59,999	________	$60,000 to $69,999
________	$70,000 to $79,999	________	$80,000 to $89,999
________	$90,000 to $99,999	________	$100,000 to $124,999
________	$125,000 to $149,999	________	$150,000 to $199,999
________	$200,000 to $249,999	________	$250,000 or over

8. Which of the following describes your current **marital status**?

______Single and never married
______Married
______Separated
______Divorced
______Widowed

9. What is the total **number of members in your household**? ______________

10. How many **children** in your household? ___________________

11. What is the **age of the children** in your household? ________________

12. Are you **currently employed**?

______ Yes
______ No

Appendix 2

13. Which of the following best describes your **current occupation**?

________ Professional or technical (for example, accountant, artist, computer specialist, engineer, nurse, doctor, teacher)
________ Manager or administrator (non-farm)
________ Sales worker (for example, insurance salesperson, real estate salesperson, sales clerk, stockbroker)
________ Clerical worker (for example, bank teller, bookkeeping, office clerk, postal worker, secretary, teacher's aide)
________ Craftsworker (for example, baker, carpenter, electrician, foreman, jeweler, mechanic, plumber, tailor)
________ Machine operator or laborer (for example, bus driver, conductor, factory worker, truck driver)
________ Farmer, farm manager, or farm laborer
________ Service worker or private household worker (for example, barber, bartender, cook, firefighter, police officer, waiter)
________ Military
________ Homemaker
________ Unable to work
________ Student
________ Other (Please specify: ______________________________)

Appendix 2

Condition #:__ Participant #: _____________

PART II: FOOD PREFERENCE AND SHOPPING EXPERIENCE

1. Did you experience any price promotion at the grocery store you just shopped?
   ______Yes
   ______No (please proceed to question #3)

2. If you answered YES to question #1, what type of price promotion did you experience at the grocery store you just shopped?

   ____ 1% TAX ______9% CASH BACK

3. How do you usually commute to the grocery store?
   ______Car
   ______Bus
   ______Walk
   ______Other (please list) _________________________

4. Who shops for grocery items for your family?
   ______Myself alone
   ______Me with my family
   ______Another family member

5. Who usually makes grocery shopping decisions?
   ______I usually make grocery shopping decisions
   ______My family and I usually decide jointly

6. How long ago did you last consume any food (Not drink)?

   ____ less than 1 hour
   ____ between 1 and 2 hours
   ____ between 2 and 3 hours
   ____ between 3 and 4 hours
   ____ more than 5 hours

7. How would you rate how hungry you currently feel? Please circle the number that best matches **your level of perception of hunger** using a 5-point scale with 1 for "*Not hungry at all*" and 5 for "*Very hungry.*"

Not hungry at all	1	2	3	4	5	Very hungry

Appendix 2

8. How many times in a month/week do you/your family typically have a meal in a restaurant?
    ______ Never
    ______ Once a month
    ______ 2-3 times a month
    ______ Once a week
    ______ 2-3 times a week
    ______ more than 3 times a week

9. How many times in a month/week do you eat fast food?
    ______ Never
    ______ Once a month
    ______ 2-3 times a month
    ______ Once a week
    ______ 2-3 times a week
    ______ more than 3 times a week

10. What kind of milk do you usually buy for your family's daily consumption?
    ______ Vitamin d/full fat milk
    ______ 2% low fat milk
    ______ 1% low fat milk
    ______ Both full fat and low fat milk
    ______ No milk

Appendix 2

11. **DIRECTION**: Please circle the number that best matches **your level of agreement with each of the following statements** using a 5-point scale [71] with 1 for "*Strongly Disagree*" and 5 for "*Strongly Agree.*"

	**Strongly Disagree**	**Disagree**	**Neutral**	**Agree**	**Strongly Agree**
I am very concerned about low prices, but I am equally concerned about product quality	1	2	3	4	5
When grocery shopping, I compare the prices of different brands to be sure that I get the best value for money.	1	2	3	4	5
When purchasing a product, I always try to maximize the quality I get for the money I spend.	1	2	3	4	5
When I buy products, I like to be sure that I am getting my money's worth.	1	2	3	4	5
I generally shop around for lower prices on products, but they still must meet certain quality requirements before I will buy them.	1	2	3	4	5
When I shop, I usually compare the "price per ounce" information for brands I normally buy.	1	2	3	4	5
I always check prices at the grocery store to be sure I get the best value for the money I spend.	1	2	3	4	5

Appendix 2

Condition #:__   Participant #: ______________

PART III: EXIT QUESTIONAIRE

1. Did you experience any price promotion at the grocery store you just shopped?
   ______Yes
   ______No (please proceed to question #3)

2. If you answered YES to question #1, what type of price promotion did you experience at the grocery store you just shopped?

   ____ 1% TAX   ______9% CASH BACK

DIRECTION: Please circle the number that best matches **your level of agreement with each of the following statements** using a 5-point scale with 1 for "*Strongly Disagree*" and 5 for "*Strongly Agree.*"

3. How do you shop for your family?

	**Strongly Disagree**	**Disagree**	**Neutral**	**Agree**	**Strongly Agree**
While grocery shopping for my family, food price is very important to me.	1	2	3	4	5
When I see a change in price for a food item, I always check the price of a similar food on the shelf.	1	2	3	4	5
It is very important for me and my family to have fresh fruits in our daily lunch and dinner menus.	1	2	3	4	5
It is very important for me and my family to have fresh vegetables in our daily lunch and dinner menus.	1	2	3	4	5

4. How did lower **TAX (1% TAX)** affect your grocery shopping behavior?

	**Strongly Disagree**	**Disagree**	**Neutral**	**Agree**	**Strongly Agree**
I mostly bought more of those foods which had lower TAX.	1	2	3	4	5
I was encouraged to buy healthy foods which had lower TAX.	1	2	3	4	5
I did not understand the lower TAX promotion.	1	2	3	4	5
I understood but ignored the lower TAX promotion.	1	2	3	4	5

Appendix 2

5. How do you feel about the lower **TAX (1% TAX)** promotion?

	**Strongly Disagree**	**Disagree**	**Neutral**	**Agree**	**Strongly Agree**
I felt happy to save.	1	2	3	4	5
I felt excited about the lower TAX promotion.	1	2	3	4	5
I did not care about the lower TAX promotion.	1	2	3	4	5

6. How did lower **TAX** impact your food choices?

	**Strongly Disagree**	**Disagree**	**Neutral**	**Agree**	**Strongly Agree**
At the grocery store that I just shopped, lower TAXES encouraged me to buy more healthy food.	1	2	3	4	5

7. How did **CASH BACK (9% CASH BACK)** affect your grocery shopping?

	**Strongly Disagree**	**Disagree**	**Neutral**	**Agree**	**Strongly Agree**
I mostly bought more of those food which had CASH BACK.	1	2	3	4	5
I was encouraged to buy healthy foods which had CASH BACK.	1	2	3	4	5
I did not understand the CASH BACK promotion..	1	2	3	4	5
I understood but ignored the CASH BACK promotion	1	2	3	4	5

8. How do you feel about the **9% CASH BACK** promotion?

	**Strongly Disagree**	**Disagree**	**Neutral**	**Agree**	**Strongly Agree**
I felt happy to save.	1	2	3	4	5
I felt excited about the CASH BACK promotion.	1	2	3	4	5
I did not care about the CASH BACK promotion.	1	2	3	4	5

9. How did **CASH BACK** impact your food choices?

	**Strongly Disagree**	**Disagree**	**Neutral**	**Agree**	**Strongly Agree**
At the grocery store that I just shopped, 9% CASH BACK encouraged me to buy more healthy food.	1	2	3	4	5

Appendix 2

10. Between 1% tax and 9% cash back promotions, I like

______1% TAX **_______9% CASH BACK**

11. Please rank the **1% TAX** and **9% CASH BACK** promotions.

	**Strongly Disagree**	**Disagree**	**Neutral**	**Agree**	**Strongly Agree**
I prefer 1% TAX more than 9% CASH BACK	1	2	3	4	5
I prefer 9% CASH BACK more than 1% TAX.	1	2	3	4	5
I like both 1% TAX and 9% CASH BACK equally	1	2	3	4	5

12. The research study was about

____
